# Does progestin-only contraceptive use after pregnancy affect recovery from pelvic girdle pain? A prospective population study

**DOI:** 10.1371/journal.pone.0184071

**Published:** 2017-09-11

**Authors:** Elisabeth Krefting Bjelland, Katrine Mari Owe, Hedvig Marie Egeland Nordeng, Bo Lars Engdahl, Per Kristiansson, Siri Vangen, Malin Eberhard-Gran

**Affiliations:** 1 Health Services Research Unit, Akershus University Hospital, Lørenskog, Norway; 2 Department of Health and Aging, Norwegian Institute of Public Health, Oslo, Norway; 3 Department of Obstetrics and Gynecology, Akershus University Hospital, Lørenskog, Norway; 4 Norwegian National Advisory Unit on Women’s Health, Oslo University Hospital, Rikshospitalet, Oslo, Norway; 5 Department of Child Health, Norwegian Institute of Public Health, Oslo, Norway; 6 PharmacoEpidemiology and Drug Safety Research Group, School of Pharmacy, University of Oslo, Oslo, Norway; 7 Department of Public Health and Caring Sciences, Uppsala University, Uppsala, Sweden; 8 Institute of Clinical Medicine, Campus Ahus, University of Oslo, Lørenskog, Norway; Universite Clermont Auvergne, FRANCE

## Abstract

**Objective:**

To estimate associations of progestin-only contraceptives with persistent pelvic girdle pain 18 months after delivery.

**Methods:**

Prospective population based cohort study during the years 2003–2011. We included 20,493 women enrolled in the Norwegian Mother and Child Cohort Study who reported pelvic girdle pain in pregnancy week 30. Data were obtained by 3 self-administered questionnaires and the exposure was obtained by linkage to the Prescription Database of Norway. The outcome was pelvic girdle pain 18 months after delivery.

**Results:**

Pelvic girdle pain 18 months after delivery was reported by 9.7% (957/9830) of women with dispense of a progestin-only contraceptive and by 10.5% (1114/10,663) of women without dispense (adjusted odds ratio 0.93; 95% CI 0.84–1.02). In sub-analyses, long duration of exposure to a progestin intrauterine device or progestin-only oral contraceptives was associated with reduced odds of persistent pelvic girdle pain (*P*_trend_ = 0.021 and *P*_trend_ = 0.005). Conversely, long duration of exposure to progestin injections and/or a progestin implant was associated with modest increased odds of persistent pelvic girdle pain (*P*_trend_ = 0.046). Early timing of progestin-only contraceptive dispense following delivery (≤3 months) was not significantly associated with persistent pelvic girdle pain.

**Conclusions:**

Our findings suggest a small beneficial effect of progestin intrauterine devices and progestin-only oral contraceptives on recovery from pelvic girdle pain. We cannot completely rule out an opposing adverse effect of exposure to progestin injections and/or progestin implants. However, the modest increased odds of persistent pelvic girdle pain among these users could be a result of unmeasured confounding.

## Introduction

Pelvic girdle pain is commonly reported during pregnancy [1] and diminishes shortly after delivery in most women [2]. Nevertheless, approximately 2–3% of all women continue to experience pain and disability that can impact work participation, activities of daily living and quality of life for years [3]. Despite possibly substantial consequences, modifiable factors that may influence recovery of pelvic girdle pain have been insufficiently studied.

Although the underlying mechanisms of pelvic girdle pain likely are multidimensional [4], both endogenous [5–7] and exogenous [8–10] hormonal factors may play a role. Two large observational studies have reported an inverse association between age at menarche and pelvic girdle pain [6, 7], suggesting an influence of endogenous hormonal factors. Recently, we observed that the use of a progestin intrauterine device during the last year before pregnancy and long-term exposure to progestin-only contraceptive pills were associated with the development of pelvic girdle pain during pregnancy [10]. Exacerbation of symptoms following the insertion of a progestin intrauterine device has been observed in the clinic [11, 12] although effects of hormonal contraceptives on recovery from pelvic girdle pain have barely been studied. One Swedish cross-sectional study 6 months after delivery observed no association of oral contraceptives with pelvic girdle pain; however, this study included only 639 women and the type of oral contraceptive was not specified [13]. Consequently, large prospective studies with long follow-up time and detailed information about hormonal contraceptives are needed to determine potential impact on recovery.

Because of possible negative effects on breast milk production and quality, progestin-only contraceptives are currently first-line choice during lactation, rather than combined estrogen/progestin contraceptives [14, 15]. The aim of this study was to investigate associations between dispensed progestin-only contraceptives and persistent pelvic girdle pain 18 months after delivery among 20,493 women with pelvic girdle pain in pregnancy.

## Materials and methods

### Study design, study population and follow up

During the years 1999–2008, the Norwegian Institute of Public Health sought to recruit all pregnant women scheduled to give birth in Norway into the Norwegian Mother and Child Cohort Study (www.fhi.no) [16], which allows linkage to several Norwegian health registries. The women were invited in connection with the routine ultrasound examination that takes place during pregnancy weeks 17–18. The study had no exclusion criteria, and 40.6% of the invited women agreed to participate. Each woman could participate with more than 1 pregnancy. The current study is based on version 8 of the quality-assured data files released for research in 2014.

Data were obtained through 3 self-administered questionnaires that were sent and returned by mail. The first and second questionnaires, which were completed during the second trimester of pregnancy [mean 17.1 weeks, standard deviation (SD) 2.0 weeks] and the third trimester (mean 30.5 weeks, SD 1.3 weeks), included questions about sociodemographic factors, general health, reproductive history, and maternal health status during pregnancy. The respondents completed the third questionnaire 18 months (mean 18.5 months, SD 0.4 months) after delivery. The questionnaires in pregnancy week 30 and 18 months after delivery included detailed questions about pain in the pelvis. By using the 11-digit unique ID number of Norwegian citizens, we linked questionnaire data in the Norwegian Mother and Child Cohort Study to information about dispense of hormonal contraceptives up to 18 months after delivery as registered in the Prescription Database of Norway (NorPD) [17].

**Fig 1:**
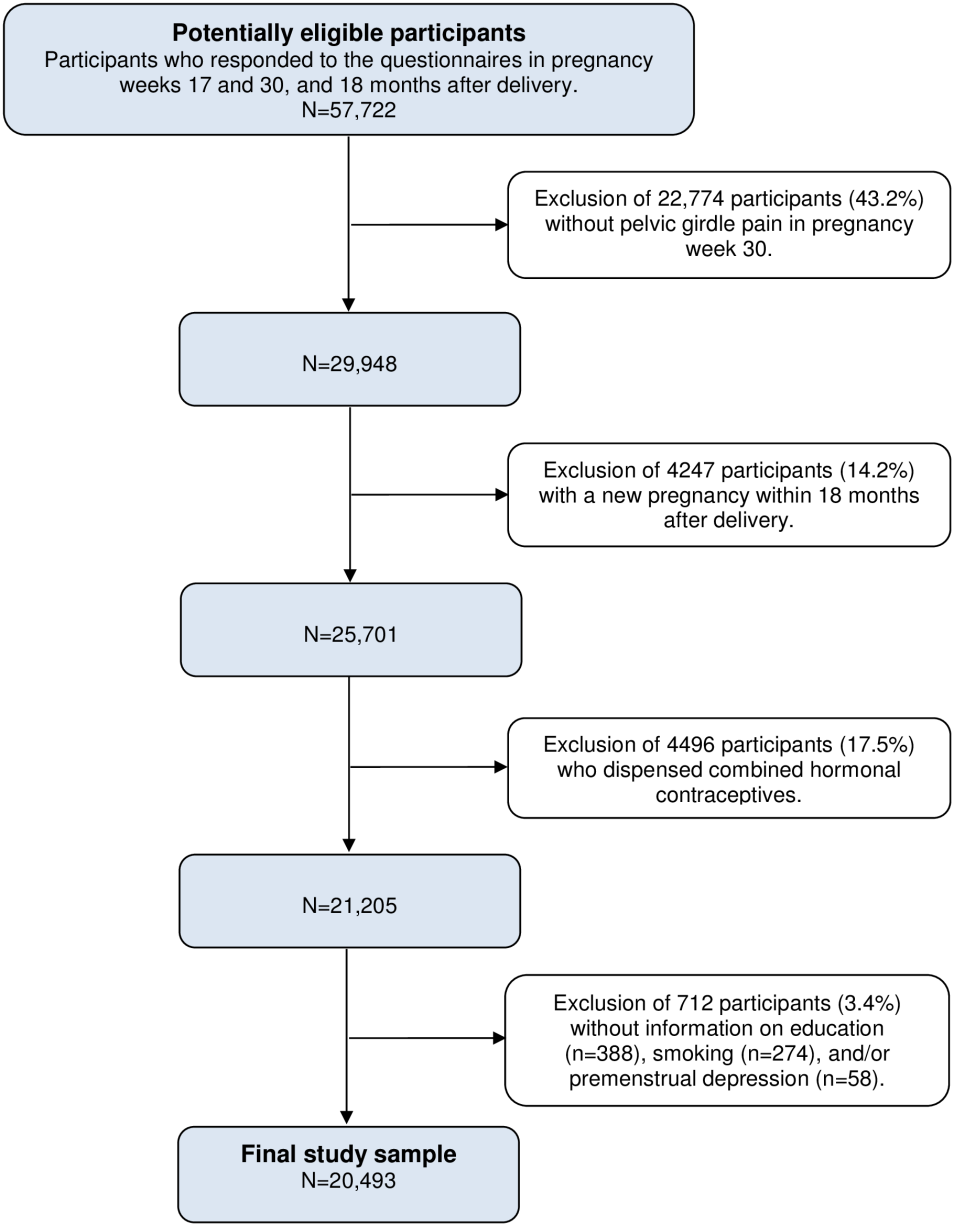
Flow chart of the study sample (2003–2011).

The overall response rate in the Norwegian Mother and Child Cohort Study was 91.0% in pregnancy week 30 and declined to 72.5% 18 months after delivery. We included 29,948 women with pelvic girdle pain in pregnancy week 30 who responded to all 3 questionnaires (Fig 1). We excluded 4247 women with a new pregnancy within 18 months following delivery and 4496 women who dispensed combined hormonal contraceptives. Finally, we excluded 712 women without information on education, smoking status, and/or premenstrual depressive symptoms, leaving 20,493 women in our study sample.

### Outcome variable

Pain in the pelvis was reported by questionnaires in pregnancy week 30 and 18 months after delivery. Our outcome variable was pelvic girdle pain 18 months after delivery and it was classified on the basis of answers to the following questions: “Did you have pain in the pelvis during the past 12 months?” (yes/no) and “If you had pain in the pelvis, where was the pain located?” One or more locations could be specified by checking a box: over the pubic symphysis the frontal part of the pelvis, and over one or both sacroiliac joints in the rear part of the pelvis. We defined pelvic girdle pain as having reported pain at any of these pelvic sites. In addition, the women had to respond positively to one of the following questions: “Do you currently wake up at night because of pelvic girdle pain?” (yes/no) and “Do you currently use crutches because of pelvic girdle pain?” (yes/no). These criteria were chosen to ensure that clinically important pelvic girdle pain was present 18 months after delivery.

### Exposure variables

The NorPD was established in 2004 for surveillance and research purposes and holds information about all dispensed prescribed medication, including hormonal contraceptives [17]. All pharmacies in Norway record and transfer information about dispensed medication electronically through Statistics Norway to the Norwegian Institute of Public Health, which is the holder of the NorPD. We used information about anatomical therapeutic chemical classification system (ATC) codes and the time of dispense relative to the current delivery to define 1) subgroups of progestin-only contraceptives users, 2) the time period of first dispense of a progestin-only contraceptive, and 3) the duration of exposure to a progestin-only contraceptive.

For each of the 18 months subsequent to delivery, we searched the NorPD for dispense of hormonal contraceptives using the ATC codes G02B (local use) and G03A (systemic use). Combined hormonal contraceptives (estrogen and progestin) may influence breast milk production and quality, and they are normally not prescribed in Norway during lactation. Hence, we excluded all participants with dispense of combined hormonal contraceptives (estrogen and progestin) with the following ATC codes were excluded from our study: G02BB01 (vaginal ring), G03AA07, G03AA09, G03AA12, G03AA13, G03AA14 (patch), G03AB03, G03AB04, and/or G03AB08.

We classified any progestin-only contraceptive during the study period by collapsing dispense of any type of the following ATC codes: G02BA03, G03AC01, G03AC02, G03AC03, G03AC06, G03AC08, and/or G03AC09 (coded as no dispense / ≥1 dispense). Further, we classified dispense of any progestin-only contraceptives according to time period of first dispense (no dispense / ≤3 months after delivery / >3 months after delivery) and according to duration of exposure (none / 1–6 months / 7–12 months / 13–18 months). Based on the same classification strategy, we identified subgroups of progestin-only contraceptives: progestin intrauterine devices (G02BA03), progestin-only oral contraceptives (G03AC01, G03AC02, G03AC03 or G03AC09), progestin injections (G03AC06) and progestin implants (G03AC08).

If a woman had dispensed a progestin intrauterine device (levonorgestrel) or a progestin implant (etonogestrel) and no other progestin-only contraceptives later than that time point, she was, respectively, defined as a progestin intrauterine device user or as a progestin implant user from that time point and up to 18 months after delivery. Progestin injections (depot medroxyprogesterone acetate) and progestin-only oral contraceptives (norethisterone, lynestrenol, levonorgestrel or desogestrel) were dispensed in packages for 3 months use. Given that no other type of progestin-only contraceptives was later dispensed, we counted the number of packages to calculate duration of exposure (months).

### Other study factors

Based on a literature review and assumed possible underlying mechanisms, we used directed acyclic graphs to assess factors that could be a common cause of progestin-only contraceptive use and pelvic girdle pain [18]. Information about maternal age (years), parity (0 or ≥1), education (≤12 years, 13–16 years, and ≥17 years), a history of other pain conditions (low back pain before the first pregnancy, rheumatic diseases, fibromyalgia, migraine, and endometriosis), and premenstrual depressive symptoms was collected in pregnancy week 17. Premenstrual depressive symptoms were reported on the basis of the following questions: “Are you usually depressed or irritable before your menstrual period?” and “If yes, does this feeling disappear after you get your menstruation?” and coded as: (1) no symptoms; (2) symptoms alleviated after onset of menstruation; and (3) symptoms not alleviated after onset of the menstruation. Smoking during pregnancy (yes/no) was based on reports in pregnancy weeks 17 and 30.

### Statistical methods

Characteristics according to dispense of progestin-only contraceptives during 18 months following delivery is presented as proportions (%) and means (SD). Group differences were tested using the *χ*^2^ test and one way ANOVA with Bonferroni corrections. The associations of progestin-only contraceptives with persistent pelvic girdle pain 18 months after delivery were estimated as crude and adjusted odds ratios (ORs) with 95% confidence intervals (CIs) using generalized estimating equations with logit link function and exchangeable correlations to correct for possible correlations between pregnancies (>1 pregnancy per study participant). In subgroup analyses, women with dispense of >1 type of progestin-only contraceptives were excluded. Due to low statistical power in analyses of progestin injections and progestin implants, these subgroups were collapsed (systemic long-acting progestin contraceptives). In all analyses, we used no dispense of hormonal contraceptives as the reference. Final models were adjusted for age, parity, education, other pain conditions, symptoms of premenstrual depression, and smoking status. Test for trend in the OR estimates according duration of exposure to progestin-only contraceptives was assessed by including number of exposure months as a continuous variable.

Few women dispensed systemic long-acting progestin-only contraceptives, and the statistical power in analysis of this subgroup was low. Adjustment for education and the other study factors affected the estimates substantially. This observation indicated that unmeasured confounding could exist and we conducted sensitivity analyses by “correcting” the ORs and the CIs for bias terms [19]. The bias terms were calculated by assuming the strength of an unmeasured confounder, and we assessed how our estimates were affected by varying the prevalence of the confounder among exposed and unexposed. Additionally, we repeated the analyses in sub-sample of women who dispensed combined hormonal contraceptives (estrogen/progestin). A 5% significance level was chosen for the analyses. All analyses were performed using Stata SE version 14.0 (StataCorp., Texas, USA).

### Ethical considerations

The study was approved by the Regional Committee for Medical Research Ethics (2012/2235/REK south-east D) and by the Norwegian Data Protection Authority (13/00517-2/EOL). Informed consent was obtained from all individual participants included in the study.

## Results

At inclusion, the women were on average 30.6 years old (SD 4.3 years), 35.0% were first-time mothers and 70.3% had >12 years of education (Table 1). By 18 months following delivery, 16.4% had dispensed a progestin intrauterine device, 25.4% progestin-only oral contraceptives, 0.6% progestin injections, 1.0% a progestin implant, and 4.6% had dispensed >1 progestin-only contraceptive. Eighteen months after delivery, 10.1% (2071/20,493) of the women still reported pelvic girdle pain. These women were more often multiparous, had less education, and were more likely smokers than women who recovered. They also reported more premenstrual depressive symptoms and other pain conditions.

**Table 1:** Characteristics of the study sample by dispense of progestin-only contraceptives (N = 20,493).

Characteristics	None dispensed N = 10,663	Progestin intrauterine device N = 3365	Progestin-only oral contraceptives N = 5196	Systemic long-acting progestin contraceptives N = 333	≥1 type dispensed N = 936	All women N = 20,493
	No. (%)	No. (%)	No. (%)	No. (%)	No. (%)	No. (%)
Age, years (mean, SD)	31.2 (4.4)	30.8 (4.2)	29.6 (4.2)	29.4 (4.8)	30.1 (4.1)	30.6 (4.3)
First-time mother	3709 (34.8)	601 (17.9)	2556 (49.2)	89 (26.7)	220 (23.5)	7175 (35.0)
Educational level						
*≤ 12 years*	3096 (29.0)	978 (29.1)	1518 (29.2)	183 (55.0)	314 (33.6)	6089 (29.7)
*13–16 years*	4560 (42.8)	1513 (45.0)	2364 (45.5)	121 (36.3)	406 (43.4)	8964 (43.7)
*≥ 17 years*	3007 (28.2)	874 (26.0)	1314 (25.3)	29 (8.7)	216 (23.1)	5440 (26.6)
Other pain conditions	3571 (33.5)	1121 (33.3)	1742 (33.5)	123 (36.9)	292 (31.2)	6849 (33.4)
Premenstrual depression						
*No symptoms*	3293 (30.9)	1136 (33.8)	1799 (34.6)	114 (34.2)	364 (38.9)	6706 (32.7)
*Symptoms alleviated*	6562 (61.5)	1998 (59.4)	3003 (57.8)	173 (52.0)	511 (54.6)	12,247 (59.8)
*Symptoms not alleviated*	808 (7.6)	231 (6.9)	394 (7.6)	46 (13.8)	61 (6.5)	1540 (7.5)
Smoking status	958 (9.0)	335 (10.0)	507 (9.8)	67 (20.1)	104 (11.1)	1971 (9.6)

*SD*, standard deviation.

### Any progestin-only contraceptives

Among women with dispense of any progestin-only contraceptive, 9.7% (957/9830) reported pelvic girdle pain 18 months after delivery compared to 10.5% (1114/10,663) without dispense (crude OR 0.92; 95% CI: 0.84–1.01) (Table 2). Virtually no change in the OR estimate was observed after adjustment for age, parity, education, other pain conditions, symptoms of premenstrual depression, and smoking status. Also, exposure to any progestin-only contraceptive during the first 3 months following delivery was not significantly associated with persistent pelvic girdle pain. However, exposure to any progestin-only contraceptive for >12 months was associated with reduced odds of persistent pain when compared with no exposure (adjusted OR 0.85; 95% CI: 0.76–0.96) (*P*_trend_ = 0.009).

**Table 2:** Associations of any progestin-only contraceptives and pelvic girdle pain 18 months after delivery (N = 20,493).

	Total	Pelvic girdle pain 18 months after delivery				
		No.cases (%)	Crude OR (95% CI)	*P*_trend_	Adjusted OR ^a^ (95% CI)	*P*_trend_
**Any progestin-only contraceptives**						
*No dispense*	10,663	1114 (10.5)	reference		reference	
*≥1 dispense*	9830	957 (9.7)	0.92 (0.84–1.01)		0.93 (0.84–1.02)	
Period of first dispense						
*No dispense*	10,663	1114 (10.5)	reference		reference	
*≤3 months after delivery*	5848	556 (9.5)	0.89 (0.80–0.99)		0.91 (0.82–1.02)	
*>3 months after delivery*	3982	401 (10.1)	0.96 (0.86–1.09)		0.95 (0.84–1.07)	
Duration of exposure						
*No dispense*	10,663	1114 (10.5)	reference		reference	
*1–6 months*	3046	329 (10.8)	1.02 (0.90–1.17)		1.05 (0.92–1.19)	
*7–12 months*	1994	185 (9.5)	0.90 (0.76–1.06)		0.93 (0.79–1.10)	
*13–18 months*	4840	443 (9.2)	0.86 (0.77–0.97)	0.009	0.85 (0.76–0.96)	0.008

The associations were estimated as crude and adjusted odds ratios (ORs) with 95% confidence intervals (CIs) using GEE with logit link function and exchangeable correlations. Tests for trend were conducted by including number of exposure months as a continuous variable.

^a^ Estimates adjusted for age, parity, education, other pain conditions, symptoms of premenstrual depression, and smoking status.

### Progestin intrauterine devices

Among women with dispense of a progestin intrauterine device, 9.8% reported pelvic girdle pain 18 months after delivery compared to 10.5% without dispense (adjusted OR 0.89; 95% CI: 0.78–1.01) (Table 3). Exposure to a progestin intrauterine device during the first 3 months was not associated with pelvic girdle pain. A duration of exposure of >12 months was significantly associated with reduced odds of persistent pain when compared to no exposure (adjusted OR 0.83; 95% CI: 0.72–0.96) (*P*_trend_ = 0.021).

**Table 3:** Associations of progestin intrauterine devices and pelvic girdle pain 18 months after delivery (N = 14,028).

	Total	Pelvic girdle pain 18 months after delivery				
		No.cases (%)	Crude OR (95% CI)	*P*_trend_	Adjusted OR ^a^ (95% CI)	*P*_trend_
**Progestin intrauterine devices**						
*No dispense*	10,663	1114 (10.5)	reference		reference	
*≥1 dispense*	3365	330 (9.8)	0.93 (0.82–1.06)		0.89 (0.78–1.01)	
Period of first dispense						
*No dispense*	10,663	1114 (10.5)	reference		reference	
*≤3 months after delivery*	1991	192 (9.6)	0.91 (0.77–1.07)		0.87 (0.74–1.03)	
*>3 months after delivery*	1374	138 (10.0)	0.96 (0.80–1.16)		0.91 (0.75–1.10)	
Duration of exposure						
*No dispense*	10,663	1114 (10.5)	reference		reference	
*1–6 months*	194	29 (15.0)	1.50 (1.01–2.23)		1.37 (0.91–2.05)	
*7–12 months*	372	42 (11.3)	1.11 (0.81–1.53)		1.06 (0.76–1.46)	
*13–18 months*	2799	259 (9.3)	0.87 (0.75–1.00)	0.089	0.83 (0.72–0.96)	0.021

The associations were estimated as crude and adjusted odds ratios (ORs) with 95% confidence intervals (CIs) using GEE with logit link function and exchangeable correlations. Tests for trend were conducted by including number of exposure months as a continuous variable.

^a^ Estimates adjusted for age, parity, education, other pain conditions, symptoms of premenstrual depression, and smoking status.

### Progestin-only oral contraceptives

Among women who dispensed progestin-only oral contraceptives, 8.9% reported pelvic girdle pain compared to 10.5% without dispense (crude OR 0.84; 95% CI: 0.75–0.94) (Table 4). After adjustment for confounding factors, the association was attenuated (adjusted OR 0.89; 95% CI 0.79–1.00, *P* = 0.051). However, both dispense of progestin-only oral contraceptives after the first 3 months (adjusted OR 0.82; 95% CI: 0.67–0.99) and long-term exposure (*P*_trend_ = 0.005) were associated with reduced odds of persistent pain.

**Table 4:** Associations of progestin-only oral contraceptives and pelvic girdle pain 18 months after delivery (N = 15,859).

	Total	Pelvic girdle pain 18 months after delivery				
		No.cases (%)	Crude OR (95% CI)	*P*_trend_	Adjusted OR ^a^ (95% CI)	*P*_trend_
**Progestin-only oral contraceptives**						
*No dispense*	10,663	1114 (10.5)	reference		reference	
*≥1 dispense*	5196	462 (8.9)	0.84 (0.75–0.94)		0.89 (0.79–1.00)	
Period of first dispense						
*No dispense*	10,663	1114 (10.5)	reference		reference	
*≤3 months after delivery*	3667	334 (9.1)	0.86 (0.75–0.97)		0.92 (0.81–1.05)	
*<3 months after delivery*	1529	128 (8.4)	0.78 (0.65–0.94)		0.82 (0.67–0.99)	
Duration of exposure						
*No dispense*	10,663	1114 (10.5)	reference		reference	
*1–6 months*	2442	251 (10.3)	0.98 (0.85–1.13)		1.02 (0.88–1.18)	
*7–12 months*	1212	87 (7.2)	0.65 (0.52–0.82)		0.73 (0.58–0.92)	
*13–18 months*	1542	124 (8.0)	0.77 (0.62–0.91)	<0.001	0.82 (0.67–1.00)	0.005

The associations were estimated as crude and adjusted odds ratios (ORs) with 95% confidence intervals (CIs) using GEE with logit link function and exchangeable correlations. Tests for trend were conducted by including number of exposure months as a continuous variable.

^a^ Estimates adjusted for age, parity, education, other pain conditions, symptoms of premenstrual depression, and smoking status.

### Systemic long-acting progestin contraceptives

Among 333 women who dispensed progestin injections and/or a progestin implant, 16.5% reported persistent pelvic girdle pain 18 months after delivery compared to 10.5% without dispense (crude OR 1.66; 95% CI: 1.19–2.22) (Table 5). After adjustment, the association was substantially attenuated (adjusted OR 1.33; 95% CI: 0.97–1.82). Users of systemic long-acting progestin contraceptives had lowest education, and the highest proportions of smokers and women with other pain conditions (Table 1). Additionally, exposure to systemic long-acting progestin contraceptives during the first 3 months following delivery was not significantly associated with persistent pelvic girdle pain. Nevertheless, the odds of persistent pelvic girdle pain increased by increasing months of exposure to systemic long-acting progestin contraceptives (*P*_trend_ = 0.046).

**Table 5:** Associations of systemic long-acting progestin contraceptives and pelvic girdle pain 18 months after delivery (N = 10,996).

	Total	Pelvic girdle pain 18 months after delivery				
		No.cases (%)	Crude OR (95% CI)	*P*_trend_	Adjusted OR ^a^ (95% CI)	*P*_trend_
**Systemic long-acting progestin contraceptives**						
*No dispense*	10,663	1114 (10.5)	reference		reference	
*≥1 dispense*	333	55 (16.5)	1.66 (1.19–2.22)		1.33 (0.97–1.82)	
Period of first dispense						
*No dispense*	10,663	1114 (10.5)	reference		reference	
*≤ 3months after delivery*	191	31 (16.2)	1.54 (1.01–2.35)		1.36 (0.90–2.06)	
*>3 months after delivery*	142	24 (16.9)	1.74 (1.12–2.70)		1.29 (0.83–2.02)	
Duration of exposure						
*No dispense*	10,663	1114 (10.5)	reference		reference	
*1–6 months*	99	16 (16.2)	1.50 (0.83–2.73)		1.28 (0.71–2.29)	
*7–12 months*	64	10 (15.6)	1.59 (0.81–3.13)		1.18 (0.61–2.28)	
*13–18 months*	170	29 (17.1)	1.71 (1.12–2.60)	0.002	1.42 (0.93–2.18)	0.046

The associations were estimated as crude and adjusted odds ratios (ORs) with 95% confidence intervals (CIs) using GEE with logit link function and exchangeable correlations. Tests for trend were conducted by including number of exposure months as a continuous variable.

^a^ Estimates adjusted for age, parity, education, other pain conditions, symptoms of premenstrual depression, and smoking status.

### Sensitivity analysis

Among 3693 women with ≤12 years of education, 55.0% were exposed to systemic long-acting progestin contraceptives and 29.0% did not dispense any hormonal contraceptives (Table 1). In regard to progestin intrauterine devices and progestin-only oral contraceptives, only small differences were observed between exposed and non-exposed. The bias analyses indicate that an unmeasured confounder with an OR of 2.00 and a prevalence of 70.0% among exposed and 30.0% among unexposed would completely explain away the estimated association between systemic long-acting progestin contraceptives and pelvic girdle pain (corrected adjusted OR 1.02; 95% CI: 0.74–1.39).

In additional analyses of a sub-sample of women who dispensed combined hormonal contraceptives only, no statistically significant association with persistent pelvic girdle pain was observed (data not shown).

## Discussion

In this prospective population based study with linkage to the NorPD, exposure to any progestin-only contraceptive did not adversely affect recovery. Exposure to a progestin intrauterine device or progestin-only oral contraceptives for 13–18 months was associated with 17 to 18 percent reduced odds of persistent pelvic girdle pain. Conversely, exposure to progestin injections and/or a progestin implant tended to increase the odds of persistent pelvic girdle pain.

We followed a large number of women from all over Norway 18 months following delivery and obtained information about progestin-only contraceptives by linkage to the NorPD. We acknowledge some limitations, however. The outcome pelvic girdle pain 18 months after delivery was based on self-reports, and not verified by clinical assessment. To assure presence of clinically important pelvic girdle pain, we combined reports of pain in the pelvis during the preceding 12 months with reports of current daily function in terms of the use of crutches and/or of current waking up at night because of pelvic girdle pain. This designation of pelvic girdle pain may have led to a misclassification of women who still experienced pain but were less functionally disabled. Therefore, erroneous reporting of pelvic girdle pain may have biased the estimates towards the null.

The Norwegian Mother and Child Cohort study does not provide information about progestin-only contraceptive use after pregnancy, and reliance on the NorPD records may have led to imprecise classification of the exposure. First, dispense of a progestin-only contraceptive does not necessarily imply that the woman used it, which may have led to overestimation of the exposure. Second, underestimation could have occurred if a woman started to use a progestin-only contraceptive that she had dispensed, but not used, before the present pregnancy. Third, some progestin intrauterine devices are dispensed directly by the clinician and not recorded in the NorPD. Therefore, our reference group may include some users of a progestin intrauterine device. Finally, the NorPD lack information about copper intrauterine devices that have been associated with dysmenorrhea and pelvic pain [20]. However, there is no reason to believe that misclassification of progestin-only contraceptives is systematically related to pelvic girdle pain and our estimated associations likely represent underestimates.

Women exposed to systemic long-acting progestin contraceptives tended to report more pelvic girdle pain 18 months after delivery than unexposed. Although we adjusted for many factors that may affect dispense of progestin-only contraceptives and also the outcome under study, residual confounding may remain. Users of systemic long-acting progestin contraceptives differ in many ways from users of progestin intrauterine devices or progestin-only oral contraceptives, and adverse effects could be a result of insufficient adjustment for confounding [21]. Low education was the strongest confounder of the association with an adjusted OR of 2.03 for persistent pelvic girdle pain (data not shown) and had a prevalence of 55.0% among exposed and 29.0% among unexposed (Table 1). We performed sensitivity analyses showing that the adverse effect could be completely explained away by a confounder of the strength of 2.00 and a prevalence of 70.0% among exposed and 30.0% among unexposed. We adjusted for many confounding factors and the possible existence of an unmeasured confounder with greater effect than education seems implausible. In addition, the statistical power in the analyses of systemic long-acting progestin contraceptives was low, and we cannot completely rule out an adverse effect.

We are aware of one study of 639 Swedish women with pelvic girdle pain in pregnancy that assessed the association of current oral contraceptive use with pelvic girdle pain 6 months after delivery [13]. Current oral contraceptives included combined estrogen/progestin and progestin-only oral contraceptives, though it was noted that the majority actually used progestin-only oral contraceptives because of lactation. In agreement with our results, women who recovered from pelvic girdle pain were more likely oral contraceptive users than women with recurrent and continuous pain (17.1%, 14.5% and 6.3%, respectively). Unfortunately, their data were not prospectively collected and the small sample did not allow sufficient adjustment for confounding.

The small beneficial effects of exposure to a progestin intrauterine device or progestin-only oral contraceptives could be attributable to the fact that healthy women are inclined to choose these methods. However, our data suggest little differences between users of a progestin intrauterine device, users of progestin-only oral contraceptives, and non-users of hormonal contraceptives (Table 1). Furthermore, it has been proposed that the return of menstrual cycles may negatively affect recovery of pelvic girdle pain [22]. Hormonal contraceptives that suppress ovulation may reduce cyclic and non-cyclic pelvic pain [23, 24] and a recent study reported higher pain thresholds in users of low dose progestin-releasing contraceptives compared to non-users [25]. Hence, progestin-only contraceptives may positively influence pelvic girdle pain after pregnancy.

Our findings do not support clinical reports suggesting that the use of a progestin intrauterine device adversely influence recovery [11, 12]. On the contrary, they suggest a small beneficial effect of long-term exposure to a progestin intrauterine device. This finding is in agreement with a longitudinal Swedish study that reported less dysmenorrhea among users of a progestin intrauterine device compared to non-users [26]. However, short-term exposure to a progestin intrauterine device tended to increase the odds of persistent pelvic girdle pain while long-term exposure appeared to have beneficial effect. An intrauterine device may not always be correctly inserted, which could lead to pelvic pain and subsequent discontinuation. Hence, the women with long-term exposure to a progestin intrauterine device may represent a group of women with high tolerance to the insertion and/or to pain.

Finally, our data suggest a modest adverse effect of long-term exposure to systemic long-acting progestin-only contraceptives. Exposure to progestin injections containing medroxyprogesterone acetate has been linked to low ovarian estrogen production and impaired bone mineral density [27]. Although changes in bone mineral density seem reversible following discontinuation, long-term effects of exposure during adolescence and perimenopause are unclear [28]. Also, pregnancy and lactation may impair bone mineral density [29]. Loss of bone mineral density as a result of medroxyprogesterone acetate exposure could add to loss of bone mineral density in connection with pregnancy and induce musculoskeletal disorders and pain. In this perspective, the postpartum period may represent an additional period of concern with regards to medroxyprogesterone acetate exposure and bone health. Future studies should therefore address possible interactions between exposure to progestin injections and lactation regarding musculoskeletal health.

## Conclusions

Our findings in this prospective population based cohort study suggest a small beneficial effect of long-term exposure to a progestin intrauterine device or progestin-only oral contraceptives on recovery from pelvic girdle pain. Due to few users of progestin injections and progestin implants, we cannot completely rule out an opposing adverse effect of these progestin-only contraceptives.

## Supporting information

S1 Questionnaire Questionnaire in pregnancy week 17 (in English). (PDF)

S2 Questionnaire Questionnaire in pregnancy week 30 (in English). (PDF)

S3 Questionnaire Questionnaire 18 months after delivery (in English). (PDF)

S4 Questionnaire Questionnaire in pregnancy week 17 (in Norwegian). (PDF)

S5 Questionnaire Questionnaire in pregnancy week 30 (in Norwegian). (PDF)

S6 Questionnaire Questionnaire 18 months after delivery (in Norwegian). (PDF)
