# Supplementary material for: Does progestin-only contraceptive use after pregnancy affect recovery from pelvic girdle pain? A prospective population study
Source: PLoS One. 2017 Sep 11;12(9):e0184071. doi: 10.1371/journal.pone.0184071 (PMC5593199; doi:10.1371/journal.pone.0184071)
Supplement: S1 Questionnaire — (PDF) [file pone.0184071.s001.pdf]

den norske **Mor & barn undersøkelsen**

Questionnaire 1

This questionnaire will be processed by a computer. It is therefore important that you follow these instructions:

- Please use a blue or black ballpoint pen.
- Put a cross in the box that is most relevant like this:
- Should you put a cross in the wrong box correct it by filling in the box completely like this:
- In the large green boxes write a number or a capital letter

  It is important that you only write in the white area of each box like this:

  Number: 0 1 2 3 4 5 6 7 8 9 Letter: A B C D
- When filling in a single figure in boxes containing two or more squares, please use the square to the right. Example: ☐ 5
- A number of questions in this questionnaire concern the week of pregnancy. For example, fill in week 5 for something that occurred 5 weeks after your last period.
- Specific information concerning, for example, medication or profession should be written in the boxes or on the lines provided. Please write clearly in CAPITAL LETTERS.
- Remember to provide the date when you completed the questionnaire.

Please return the completed questionnaire in the stamped addressed envelope provided.

Date on which the questionnaire was completed ☐☐ ☐☐ ☐☐☐☐ (write the year with 4 numbers, e.g. 2000)

Day Month Year

Menstruation

1. How old were you when you had your first menstrual period?

☐☐ Years

2.How many days are there usually between the first day in your menstrual period and the first day in your next menstrual period?

☐☐ Days

3. Are you usually depressed or irritable before your period?

☐ No
☐ Yes, but just slightly
☐ Yes, noticeably
☐ Yes, very much

4. If yes, does this feeling disappear after you get your period?

☐ No
☐ Yes

5. Were your periods regular the year before you became pregnant?

☐ No
☐ Yes

6. During the last year before you became pregnant, did you lose your period for more than three months?

☐ No
☐ Yes, due to an earlier pregnancy
☐ Yes, for other reasons

7. Date of first day of last menstrual period.

☐☐ ☐☐ ☐☐☐☐

Day Month Year

8. Did your last menstrual period come at the expected time?

☐ No
☐ Yes

9. Are you certain about the date of first day of last menstrual period?

☐ Certain
☐ Uncertain

10. Describe the duration, amount of bleeding and menstrual pains of your last period ?

	As usual	More than usual	Less than usual
Duration	☐	☐	☐
Amount of bleeding	☐	☐	☐
Menstrual pains	☐	☐	☐

Questionnaire 1C - MB - 40.000 - 0303

Contraception and pregnancy

11. Have you/your partner at any time during the last year used the following methods to avoid becoming pregnant? (Fill in all that apply.)

- ☐ Condom
- ☐ Diaphragm
- ☐ IUD
- ☐ Hormone IUD
- ☐ Hormone injection
- ☐ Mini pill
- ☐ Pill
- ☐ Spermicides (foam, suppositories, cream)
- ☐ Safe period
- ☐ Withdrawal
- ☐ No such methods
- ☐ Other___________________________

12. If you have used the pill/mini-pill, how long altogether have you used them?

	Pill	Mini-pill
Less than one year	☐	☐
1-3 years	☐	☐
4-6 years	☐	☐
7-9 years	☐	☐
10 years or more	☐	☐

13. If you have used the pill/mini-pill, how old were you when you first used it?

☐☐ Years old

14. Were you taking the pill/mini-pill during the last 4 months before this pregnancy?

- ☐ No
- ☐ Yes

15. If yes, how long before your last menstrual period did you stop taking the pill/mini-pill?

☐☐ Weeks

16. Was this pregnancy planned?

- ☐ No
- ☐ Yes

17. If yes, how many months did you have regular intercourse without contraception before you became pregnant?

- ☐ Less than I month
- ☐ 1-2 months
- ☐ 3 months or more

☐☐ Number of months if more than 3

18. Did you become pregnant even though you or your partner used contraceptives?

- ☐ No (proceed to question 21)
- ☐ Yes

19. If yes, which type? (Fill in all that apply.)

- ☐ Condom
- ☐ Diaphragm
- ☐ IUD
- ☐ Hormone IUD
- ☐ Hormone injection
- ☐ Mini pill
- ☐ Pill
- ☐ Spermicides (foam, suppositories, cream)
- ☐ Safe period
- ☐ Withdrawal
- ☐ Other___________________________

20. If you became pregnant while using an IUD, has it now been removed?

- ☐ No
- ☐ Yes

21. How long have you and the baby's father had a sexual relationship?

☐☐ months or ☐☐ years

22. How often did you have sexual intercourse during the four weeks before you became pregnant and during the last four weeks?

	Before	Now
Every day	☐	☐
5-6 times a week	☐	☐
3-4 times a week	☐	☐
1-2 times a week	☐	☐
1-2 times every two weeks	☐	☐
Less than 1-2 times every 2 weeks	☐	☐
Never	☐	☐

23. Have you ever been treated for infertility?

- ☐ No
- ☐ Yes

24. If yes, was it in connection with this pregnancy or an earlier pregnancy and what type of treatment did you have? (Fill in all that apply.)

	Earlier Pregnancy	This Pregnancy
Fallopian tube surgery	☐	☐
Other surgery	☐	☐
Medication for endometriosis	☐	☐
Hormone treatment	☐	☐
Insemination (injection of sperm)	☐	☐
IVF (test tube) method	☐	☐
Other	☐	☐

25. Have you been given information about having an amniocentesis performed?

- ☐ No
- ☐ Yes

26. What was your blood pressure at your first antenatal visit? (Check your medical card.)

☐☐☐ / ☐☐☐ E.g. 150 / 95

27. What did you weigh at the time you became pregnant and what do you weigh now (in kilograms)?

When I became pregnant : ☐☐ kg Now: ☐☐ kg

28. How tall are you?

☐☐ cm

29. How tall is the baby's father?

☐☐ cm

30. How much does the baby's father weigh (in kilograms)?

☐☐ kg

Previous pregnancies

31. Have you been pregnant before? (Include all pregnancies that ended in abortion, miscarriage or stillbirth as well)

☐ No (proceed to question 36)
☐ Yes

32. If yes, fill in for all earlier pregnancies. Include all pregnancies that ended in abortion, miscarriage or stillbirth as well as ectopic pregnancies. State the year the pregnancy began, how many kilos you gained during the pregnancy and the number of months you breast-fed each baby. State whether or not you smoked during earlier pregnancies.

Pregnancy Number	Year pregnancy started	Live infant born	Spontaneous abortion/ stillbirth	Termination of pregnancy	Ectopic pregnancy	Week of pregnancy for abortion/ still birth	Number of months breast feeding	Weight gain during pregnancy (in kg)	Smoked during pregnancy
1		☐	☐	☐	☐				☐
2		☐	☐	☐	☐				☐
3		☐	☐	☐	☐				☐
4		☐	☐	☐	☐				☐
5		☐	☐	☐	☐				☐
6		☐	☐	☐	☐				☐
7		☐	☐	☐	☐				☐
8		☐	☐	☐	☐				☐
9		☐	☐	☐	☐				☐
10		☐	☐	☐	☐				☐

33. Have you had any of the following problems during previous pregnancies? (Fill in all that apply.)

	No	Yes
1. Pelvic girdle pain requiring medical leave	☐	☐
2. Pelvic girdle pain requiring bed rest	☐	☐
3. Serious nausea and vomiting	☐	☐
4. Pre-eclampsia during pregnancy	☐	☐
5. Pregnancy diabetes	☐	☐
6. Sugar in urine	☐	☐
7. Problems with incontinence	☐	☐

34. If you had pelvic girdle pain in a previous pregnancy that led to bed rest or medical leave, when did the pain start?

☐☐ months after start of pregnancy

35. When did the pain stop?

☐☐ months after pregnancy

☐ still have pain

Illnesses and health problems during this pregnancy

36. Have you had bleeding from the vagina once or more during this pregnancy?

☐ No
☐ Yes

37. If yes, describe the first and last bleeding. Give the date the bleeding started, how many days the bleeding lasted and how much you bled.

	Date when bleeding started (Day / Month / Year)	No. of days variation	Amount (Enter a cross in a box indicating the amount of blood (trace blood means a few drops)
First bleeding			☐ Trace of blood ☐ More than just a trace ☐ Clots
Last bleeding			☐ Trace of blood ☐ More than just a trace ☐ Clots

If more than two episodes of bleeding write in the number of times ☐☐

38. Have you experienced any of the following illnesses or problems during this pregnancy? If you have used medication in connection with these problems give the name of the medicine, the weeks you took the medicines and how many days you took them. (Include all types of medication, both prescription and over the counter medicines in addition to alternative and herbal remedies. Do not include vitamins and dietary supplements as these are discussed elsewhere.)

Illness/health problem during this pregnancy					Use of medication during this pregnancy					
	Week of pregnancy					Week of pregnancy				Number of days taken
Illness/health problem	0-4	5-8	9-12	13+	Name of medicine taken	0-4	5-8	9-12	13+	
1 Pelvic girdle pain	☐	☐	☐	☐		☐	☐	☐	☐	
2 Abdominal pain	☐	☐	☐	☐		☐	☐	☐	☐	
3 Back pain	☐	☐	☐	☐		☐	☐	☐	☐	
4 Neck and shoulder pain	☐	☐	☐	☐		☐	☐	☐	☐	
5 Nausea	☐	☐	☐	☐		☐	☐	☐	☐	
6 Nausea with vomiting	☐	☐	☐	☐		☐	☐	☐	☐	
7 Vaginal thrush	☐	☐	☐	☐		☐	☐	☐	☐	
8 Vaginal catarrh/unusual discharge	☐	☐	☐	☐		☐	☐	☐	☐	
9 Pregnancy itch	☐	☐	☐	☐		☐	☐	☐	☐	
10 Constipation	☐	☐	☐	☐		☐	☐	☐	☐	
11 Diarrhoea/gastric flu	☐	☐	☐	☐		☐	☐	☐	☐	
12 Unusual tiredness/sleepiness	☐	☐	☐	☐		☐	☐	☐	☐	
13 Sleeping problems	☐	☐	☐	☐		☐	☐	☐	☐	
14 Heartburn/reflux	☐	☐	☐	☐		☐	☐	☐	☐	
15 Oedema	☐	☐	☐	☐		☐	☐	☐	☐	
16 Fever with rash	☐	☐	☐	☐		☐	☐	☐	☐	
17 Fever over 38.5 C	☐	☐	☐	☐		☐	☐	☐	☐	
18 Common cold	☐	☐	☐	☐		☐	☐	☐	☐	
19 Throat infection	☐	☐	☐	☐		☐	☐	☐	☐	
20 Sinusitis/ear infection	☐	☐	☐	☐		☐	☐	☐	☐	
21 Influenza	☐	☐	☐	☐		☐	☐	☐	☐	
22 Pneumonia/bronchitis	☐	☐	☐	☐		☐	☐	☐	☐	
23 Sugar in urine	☐	☐	☐	☐		☐	☐	☐	☐	
24 Protein in urine	☐	☐	☐	☐		☐	☐	☐	☐	

Previous and current illnesses and health problems

39. Do you have or have you had any of the following illnesses or health problems? If you have taken medication (tablets, mixtures, suppositories, inhalers, creams, etc.) in conjunction with the illness or health problem give the name(s) of the medication(s) and when you took them.

Illness/health problem during this pregnancy			Use of medication						
Illness/health problem	Before Pregnancy	During Pregnancy	Name of medicines	Last 6 months before pregnancy	Pregnancy week 0-4	5-8	9-12	13+	Number of days used
**Asthma/Allergy/Skin disorders**									
1 Asthma	☐	☐	______	☐	☐	☐	☐	☐	
2 Hay fever, pollen allergy	☐	☐	______	☐	☐	☐	☐	☐	
3 Animal hair allergy	☐	☐	______	☐	☐	☐	☐	☐	
4 Other allergy	☐	☐	______	☐	☐	☐	☐	☐	
5 Atopic dermatitis (childhood eczema)	☐	☐	______	☐	☐	☐	☐	☐	
6 Urticaria (hives)	☐	☐	______	☐	☐	☐	☐	☐	
7 Psoriasis	☐	☐	______	☐	☐	☐	☐	☐	
8 Other eczema	☐	☐	______	☐	☐	☐	☐	☐	
9 Cold sores (herpes)	☐	☐	______	☐	☐	☐	☐	☐	
10 Acne/pimples (serious)	☐	☐	______	☐	☐	☐	☐	☐	
**Diabetes**									
11 Diabetes treated with insulin	☐	☐	______	☐	☐	☐	☐	☐	
12 Diabetes not treated with insulin	☐	☐	______	☐	☐	☐	☐	☐	
**Heart/Blood/Metabolism/Blood vessels**									
13 Congenital heart defect	☐	☐	______	☐	☐	☐	☐	☐	
14 Other heart disease	☐	☐	______	☐	☐	☐	☐	☐	
15 High cholesterol	☐	☐	______	☐	☐	☐	☐	☐	
16 High blood pressure	☐	☐	______	☐	☐	☐	☐	☐	
17 Hypothyroidism or hyperthyroidism	☐	☐	______	☐	☐	☐	☐	☐	
18 Anaemia/low haemoglobin	☐	☐	______	☐	☐	☐	☐	☐	
19 B-12/folic acid insufficiency	☐	☐	______	☐	☐	☐	☐	☐	
**Gastrointestinal**									
20 Hepatitis/jaundice	☐	☐	______	☐	☐	☐	☐	☐	
21 Gall stones	☐	☐	______	☐	☐	☐	☐	☐	
22 Duodenal/stomach ulcer	☐	☐	______	☐	☐	☐	☐	☐	
23 Crohn's disease/ulcerative colitis	☐	☐	______	☐	☐	☐	☐	☐	
24 Celiac sprue (gluten sensitivity)	☐	☐	______	☐	☐	☐	☐	☐	
25 Other gastro-intestinal problems	☐	☐	______	☐	☐	☐	☐	☐	
**Muscle/Skeleton/Connective tissue**									
26 Arthritis (rheumatoid arthritis)/ Bechterev's reflex	☐	☐	______	☐	☐	☐	☐	☐	

Illness/health problem	Before Pregnancy	During Pregnancy	Name of medicines	Last 6 months before pregnancy	Pregnancy week 0-4	5-8	9-12	13+	Number of days used
27 Lupus (SLE)	☐	☐		☐	☐	☐	☐	☐	
28 Sciatica	☐	☐		☐	☐	☐	☐	☐	
29 Fibromyalgia	☐	☐		☐	☐	☐	☐	☐	
**Genital and urinary tract**									
30 Ovary/fallopian tube infection	☐	☐		☐	☐	☐	☐	☐	
31 Endometriosis	☐	☐		☐	☐	☐	☐	☐	
32 Uterus prolaps	☐	☐		☐	☐	☐	☐	☐	
33 Ovarian cyst	☐	☐		☐	☐	☐	☐	☐	
34 Myoma	☐	☐		☐	☐	☐	☐	☐	
35 Cervical cell changes	☐	☐		☐	☐	☐	☐	☐	
36 Herpes	☐	☐		☐	☐	☐	☐	☐	
37 Venereal warts/condyloma	☐	☐		☐	☐	☐	☐	☐	
38 Gonorrhea	☐	☐		☐	☐	☐	☐	☐	
39 Chlamydia	☐	☐		☐	☐	☐	☐	☐	
40 Kidney stones	☐	☐		☐	☐	☐	☐	☐	
41 Kidney infection/pyelonephritis	☐	☐		☐	☐	☐	☐	☐	
42 Urinary tract infections/cystitis	☐	☐		☐	☐	☐	☐	☐	
43 Incontinence	☐	☐		☐	☐	☐	☐	☐	
**Other illnesses/health problems**									
44 Anorexia/bulimia/other eating disorders	☐	☐		☐	☐	☐	☐	☐	
45 Migraine	☐	☐		☐	☐	☐	☐	☐	
46 Other headache	☐	☐		☐	☐	☐	☐	☐	
47 Epilepsy	☐	☐		☐	☐	☐	☐	☐	
48 Multiple sclerosis	☐	☐		☐	☐	☐	☐	☐	
49 Cerebral palsy	☐	☐		☐	☐	☐	☐	☐	
50 Cancer	☐	☐		☐	☐	☐	☐	☐	
51 Depression	☐	☐		☐	☐	☐	☐	☐	
52 Anxiety	☐	☐		☐	☐	☐	☐	☐	
53 Other long illness or health problems	☐	☐		☐	☐	☐	☐	☐	

Which . . . .

(Table header groups: "Illness/health problem during this pregnancy" spans Illness/health problem, Before Pregnancy, During Pregnancy; "Use of medication" spans the remaining columns.)

40. Do you have a congenital malformation/birth defect?

☐ No
☐ Yes

41. If yes, which? ________________________________

42. Do your gums bleed when you brush your teeth at present?

☐ No, rarely or never
☐ Yes, sometimes
☐ Yes, often
☐ Yes, almost always

43. If you had diabetes before you became pregnant, what was your last long-term blood sugar level (HbA1c) before you became pregnant?

☐ Less than 7.5
☐ 7.5 - 12
☐ More than 12
☐ Don't Know

Other medicines

44. Have you used other medication not previously mentioned? If yes, which and when did you take them?

Name of medication (e.g. Valium, Rohypnol, Paracetamol)	Use of medication during pregnancy weeks: Last 6 months before pregnancy	0-4	5-8	9-12	13+	Number of days used
____________________	☐	☐	☐	☐	☐	☐☐☐
____________________	☐	☐	☐	☐	☐	☐☐☐
____________________	☐	☐	☐	☐	☐	☐☐☐
____________________	☐	☐	☐	☐	☐	☐☐☐
____________________	☐	☐	☐	☐	☐	☐☐☐

Vitamins, minerals and dietary supplements

45. Do you take vitamins, minerals or other dietary supplements?

☐ No (proceed to question 49)
☐ Yes

46. If yes, fill in the table below for the vitamins and minerals found in the contents list on the vitamin package/bottle. (For instance, if you have taken cod liver oil for the last six months before becoming pregnant, enter a cross for each period under "When" (i.e. 7 crosses) and enter a cross in "Daily" under "How often").

	When did you take the supplements? Last 6 months before pregnancy			During pregnancy				In this period how often did you take this?		
	26-9 weeks	8-5 weeks	4-0 weeks	0-4 weeks	5-8 weeks	9-12 weeks	13+ weeks	Daily	4-6 times a week	1-3 times a week
1 Folate/folic acid	☐	☐	☐	☐	☐	☐	☐	☐	☐	☐
2 Vitamin B1 (Thiamine)	☐	☐	☐	☐	☐	☐	☐	☐	☐	☐
3 Vitamin B2 (Riboflavin)	☐	☐	☐	☐	☐	☐	☐	☐	☐	☐
4 Vitamin B6 (Pyridoxine)	☐	☐	☐	☐	☐	☐	☐	☐	☐	☐
5 Vitamin B12	☐	☐	☐	☐	☐	☐	☐	☐	☐	☐
6 Niacin	☐	☐	☐	☐	☐	☐	☐	☐	☐	☐
7 Pantothenic acid	☐	☐	☐	☐	☐	☐	☐	☐	☐	☐
8 Biotin	☐	☐	☐	☐	☐	☐	☐	☐	☐	☐
9 Vitamin C	☐	☐	☐	☐	☐	☐	☐	☐	☐	☐
10 Vitamin A	☐	☐	☐	☐	☐	☐	☐	☐	☐	☐
11 Vitamin D	☐	☐	☐	☐	☐	☐	☐	☐	☐	☐
12 Vitamin E	☐	☐	☐	☐	☐	☐	☐	☐	☐	☐
13 Iron	☐	☐	☐	☐	☐	☐	☐	☐	☐	☐
14 Calcium	☐	☐	☐	☐	☐	☐	☐	☐	☐	☐
15 Iodine	☐	☐	☐	☐	☐	☐	☐	☐	☐	☐
16 Zinc	☐	☐	☐	☐	☐	☐	☐	☐	☐	☐
17 Selenium	☐	☐	☐	☐	☐	☐	☐	☐	☐	☐
18 Copper	☐	☐	☐	☐	☐	☐	☐	☐	☐	☐
19 Chromium	☐	☐	☐	☐	☐	☐	☐	☐	☐	☐
20 Magnesium	☐	☐	☐	☐	☐	☐	☐	☐	☐	☐
21 Cod liver oil	☐	☐	☐	☐	☐	☐	☐	☐	☐	☐
22 Omega-3 fatty acid	☐	☐	☐	☐	☐	☐	☐	☐	☐	☐

47. Give the complete name(s) of all vitamins and dietary supplements you take. Include alternative/herbal remedies and diet products. (Write clearly in CAPITAL LETTERS.)

E.g. VITAPLEX WITH IRON

1

2

3

4

5

6

48. If you use multivitamins (with or without minerals) do these contain folic acid?

- ☐ Yes
- ☐ No
- ☐ Don't Know

Civil status and education

49. What is your civil status?

- ☐ Married
- ☐ Cohabitant
- ☐ Single
- ☐ Divorced/separated
- ☐ Widow
- ☐ Other

50. What education do you and the baby's father have? (Enter a cross indicating the highest level of education you both have completed and current studies if you are still studying.)

	You		Baby's Father	
	Completed	On-going	Completed	On-going
1 9-year secondary school	☐	☐	☐	☐
2 1-2 year high school	☐	☐	☐	☐
3 Technical high school	☐	☐	☐	☐
4 3-year high school general studies, junior college	☐	☐	☐	☐
5 Regional technical college, 4-year university degree (Bachelor's degree, nurse, teacher, engineer)	☐	☐	☐	☐
6 University, technical college, more than 4 years (Master's degree, medical doctor, PhD)	☐	☐	☐	☐
7 Other education	☐	☐	☐	☐

Work and leisure

51. What was your and the baby's father's work situation when you became pregnant? (Fill in one or several boxes for each.)

	You	Baby's Father
1 Student	☐	☐
2 At home	☐	☐
3 Intern/apprentice	☐	☐
4 Military service	☐	☐
5 Unemployed/laid off	☐	☐
6 Rehabilitation/disabled	☐	☐
7 Employed in public sector	☐	☐
8 Employed in private sector	☐	☐
9 Self-employed	☐	☐
10 Family member without steady income in family company (e.g. Farming, business)	☐	☐
11 Other	☐	☐

52. Did you have an extra job (with or without salary) when you became pregnant? (For example, accountant, hair dresser, singer in a dance band, club leader)

☐ No

☐ Yes, describe________________________________

53. Have you been absent from your usual work more than two weeks altogether during this pregnancy?

☐ No

☐ Yes

54 Are you absent from your work at the present time?

☐ No

☐ Yes

55. If yes, what is the reason for your absence? (Fill in one or several boxes.)

☐ Medical leave

☐ Leave of absence

☐ Sick child

☐ Other ________________________________

56. The usual number of paid working hours a week before you became pregnant and at present.

Before the pregnancy: ☐☐,☐ Hours

During the pregnancy : ☐☐,☐ Hours

(Questions about current work situation to be answered by anyone in paid employment, even if they are temporarily absent due to illness, being on leave or for similar reasons.)

	You	Baby's Father
**57. Describe the type of work carried out at your and the baby's father's place of work as accurately as possible.** (Write for example, hospital department for children with cancer, body shop at a garage for diesel vehicles, farming with grain and swine, work in the home.)		
**58. Occupation/title at this workplace?** (Write for example, staff nurse, mechanic, foreman, lecturer, student, cleaning assistant, housewife/at home.)		

59. Indicate the appropriate answer for each of the following questions concerning your present work situation. (Fill in only one box in each line.)

	Yes every day more than half of the working day	Yes every day less than half of the working day	Yes, periodically but not daily	Seldom or never
Do you sometimes have so much to do that your work situation becomes taxing?	☐	☐	☐	☐
Do you have to turn or bend many times in the course of an hour?	☐	☐	☐	☐
Do you work with your hands up at shoulder level or higher?	☐	☐	☐	☐
Do you work standing or walking?	☐	☐	☐	☐
Can you choose to work a little faster some days and a little slower on other days?	☐	☐	☐	☐
Are you subjected to a lot of uncomfortable background noise?	☐	☐	☐	☐
Are you subjected to a lot of background noise that makes you have to raise your voice when talking to others, even at a distance of one metre?	☐	☐	☐	☐

60. How do the following statements describe your work situation? (Fill in only one box in each line.)

	Agree	Agree mostly	Disagree mostly	Disagree completely
I have physically heavy work.	☐	☐	☐	☐
My work is very stressful	☐	☐	☐	☐
I learn a lot at work	☐	☐	☐	☐
My work is very monotonous	☐	☐	☐	☐
My work demands a lot of me.	☐	☐	☐	☐
I am able to decide how my work is to be carried out.	☐	☐	☐	☐
There is a good team spirit at my place of work.	☐	☐	☐	☐
I enjoy my work	☐	☐	☐	☐

61. When are your working hours? (Fill in one or several boxes.)

☐ Permanent day work

☐ Permanent afternoon or evening work

☐ Permanent night work

☐ Shift work or shift rotations

☐ No set times (extra help, extra shifts, temporary employment, etc.)

☐ Other

62. During your pregnancy do you lift anything that weighs more than 10 kg (10 kilos is the equivalent of a full bucket of water.)

	At Home	At Work
Seldom or never	☐	☐
Yes, less than 20 times a week	☐	☐
Yes, more than 20 times a week	☐	☐
Yes, 10-20 times a day	☐	☐
Yes, more than 20 times a day	☐	☐

63. How often have you worked with radio transmitters or radar after becoming pregnant?

- ☐ Seldom/Never
- ☐ A few times a week
- ☐ Daily
- ☐ On average more than an hour daily

64. How often do you talk on a cell phone?

- ☐ Seldom/Never
- ☐ A few times a week
- ☐ Daily
- ☐ On average more than an hour daily

65. Do your cell phone calls last more than 15 minutes?

- ☐ Never
- ☐ Seldom
- ☐ Often

66. How often have you worked with a computer monitor, laser printer or copying machine (at a distance of less than two metres) after you became pregnant?

	Computer monitor	Laser printer	Copying machine
Seldom/Never	☐	☐	☐
A few times per week	☐	☐	☐
Daily	☐	☐	☐
On average more than an hour daily	☐	☐	☐

67. How often have you worked with X-ray equipment (at a distance of less than two metres) after you became pregnant?
(This does not include treatment as a patient)

- ☐ Seldom/Never
- ☐ A few times a week
- ☐ Daily
- ☐ On average more than an hour daily

68. Have you been in contact with any of the following substances either at work or in your leisure time during the last six months? (Fill in each line.)

		No	Yes	If Yes, number of days the last 6 months (daily = 180 days)	Fill in if you have used a hood for gases or breathing protection	Fill in if you have used protective gloves
1	Lead vapours, lead dust, lead particles or lead alloys	☐	☐	☐☐☐	☐	☐
2	Chrome, arsenic, cadmium or combinations of these	☐	☐	☐☐☐	☐	☐
3	Gasoline or exhaust (does not apply to filling gasoline in your own car)	☐	☐	☐☐☐	☐	☐
4	Mercury vapours, mercury or work with amalgam fillings (does not apply to your own dental treatment)	☐	☐	☐☐☐	☐	☐
5	Disinfectants, vermin poisons	☐	☐	☐☐☐	☐	☐
6	Weed killers, insecticides, fungicides	☐	☐	☐☐☐	☐	☐
7	Oil-based paint	☐	☐	☐☐☐	☐	☐
8	Water-based or latex paint	☐	☐	☐☐☐	☐	☐
9	Paint thinner, paint-lacquer-glue remover or other solvents (e.g. lynol, turpentine, toluene, carbon tetrachloride)	☐	☐	☐☐☐	☐	☐
10	Industrial dyes or ink	☐	☐	☐☐☐	☐	☐
11	Motor oil, lubrication oil or other types of oil	☐	☐	☐☐☐	☐	☐
12	Photographic chemicals (fixatives or developers)	☐	☐	☐☐☐	☐	☐
13	Substances used in welding	☐	☐	☐☐☐	☐	☐
14	Substances used in soldering	☐	☐	☐☐☐	☐	☐
15	Formalin/formaldehyde	☐	☐	☐☐☐	☐	☐
16	Chemotherapeutic substances/chemotherapy treatment (does not apply to your own medical treatment)	☐	☐	☐☐☐	☐	☐
17	Laughing gas or other anaesthetic gases (does not apply to your own treatment as a patient)	☐	☐	☐☐☐	☐	☐
18	Other substances and conditions, describe____________________	☐	☐	☐☐☐	☐	☐

69. How often have you been to a discotheque since you became pregnant?

- ☐ 1-2 times a week
- ☐ Less often
- ☐ Never

70. Are you in contact with animals either at work or in your leisure time?

- ☐ No
- ☐ Yes

71. If yes, what sort of animals and how often are you in contact with them on a weekly basis?

	Daily	3-6 times a week	1-2 times a week	Less than 1 time a week
1 Dog	☐	☐	☐	☐
2 Cat	☐	☐	☐	☐
3 Guinea pig	☐	☐	☐	☐
4 Hamster	☐	☐	☐	☐
5 Rabbit	☐	☐	☐	☐
6 Canary or other bird	☐	☐	☐	☐
7 Aquarium fish	☐	☐	☐	☐
8 Cow	☐	☐	☐	☐
9 Pig	☐	☐	☐	☐
10 Sheep, goat	☐	☐	☐	☐
11 Horse	☐	☐	☐	☐
12 Poultry	☐	☐	☐	☐
13 Other	☐	☐	☐	☐

Housing and household

72. With whom do you live? (Fill in one or several boxes.)

☐ Spouse/partner
☐ Parents
☐ Parents-in-law
☐ Children
☐ No one
☐ Other describe________________________________

73. How many people including you live in your home?

Number of people over 18 years ☐☐
Number of people between 12 - 18 years ☐☐
Number of people between 6 - 11 years ☐☐
Number of people under 6 years ☐☐

74. How many children are at nursery school/day care?

☐ children

75. Do you or the baby's father have a mother tongue other than Norwegian?

☐ No
☐ Yes

76. If yes, which language?

	You	Baby's Father
Sámi	☐	☐
Urdu	☐	☐
English	☐	☐
Other	☐	☐

If other, which?________________________________

77. Do your parents or the baby's father's parents have a mother tongue other than Norwegian?

☐ No
☐ Yes

78. If yes, which language?

	Your Mother	Your Father	Mother of the child's father	Father of the child's father
Sámi	☐	☐	☐	☐
Urdu	☐	☐	☐	☐
English	☐	☐	☐	☐
Other	☐	☐	☐	☐

If other, which? ________________________________

79. What is your and the baby's father's yearly gross income? (Include child support, unemployment benefits and other allowances.)

Your gross income	Child's father's gross income
☐ No income	☐ No income
☐ Under 150.000 NOK	☐ Under 150.000 NOK
☐ 150-199.999 NOK	☐ 150-199.999 NOK
☐ 200-299.999 NOK	☐ 200-299.999 NOK
☐ 300-399.999 NOK	☐ 300-399.999 NOK
☐ 400-499.999 NOK	☐ 400-499.999 NOK
☐ over 500.000 NOK	☐ over 500.000 NOK
	☐ Don't know

80. Is it possible for your household to manage financially without your income?

☐ No
☐ Yes, but with difficulty
☐ Yes, without difficulty

81. What type of house do you live in?

☐ Detached house
☐ Farm
☐ Semi detached
☐ Four-flat house
☐ Maisonette
☐ Terraced flat
☐ Basement flat
☐ Apartment building
☐ Townhouse/tenement
☐ Which floor? ☐☐
☐ Other________________________________

82. Has there been damp damage, visible signs of fungus/mildew or a smell of mildew in your home in the past 3 months? (Fill in one or several boxes.)

☐ No
☐ Yes, damp damage
☐ Yes, signs of fungus and mould
☐ Yes, a smell of mildew

83. Where does your drinking water come from?

☐ Public or private water company
☐ Water from a local source (e.g. own well)

84. How many times have you moved in the last 3 years?

☐☐ times

85. Has anyone in your home had influenza, a prolonged cough, childhood disease or an illness with fever and a rash after you became pregnant?

☐ No
☐ Yes

86. If yes, which illness? (fill in one or several boxes)

☐ German measles
☐ Chicken pox
☐ Measles
☐ Roseola infantum
☐ Other fever with rash
☐ Influenza
☐ Prolonged cough
☐ Tuberculosis
☐ Hand, foot and mouth disease
☐ Other

Living habits

87. Did your mother smoke when she was pregnant with you?
- ☐ No
- ☐ Yes
- ☐ Don't Know

88. Are you exposed to passive smoking at home?
- ☐ No
- ☐ Yes

89. If yes, how many hours a day are you exposed to passive smoking?

☐☐ hours per day

90. Are you exposed to passive smoking at work?
- ☐ No
- ☐ Yes

91. If yes, how many hours a day are you exposed to passive smoking?

☐☐ hours per day

92. Did the baby's father smoke before you became pregnant?
- ☐ No
- ☐ Yes

93. Does he smoke now?
- ☐ No
- ☐ Yes

94. Have you ever smoked?
- ☐ No (proceed to question 104)
- ☐ Yes

95. Do you smoke now (after you became pregnant)?
- ☐ No
- ☐ Sometimes ☐☐ cigarettes per week
- ☐ Daily ☐☐ cigarettes per day

96. Did you smoke during the last 3 months before you became pregnant this time?
- ☐ No
- ☐ Sometimes ☐☐ cigarettes per week
- ☐ Daily ☐☐ cigarettes per day

97. How old were you when you started to smoke on a daily basis?

☐☐ Years

98. Have you stopped smoking completely?
- ☐ No
- ☐ Yes

99. If yes, how old were you when you stopped smoking?

☐☐ Years

100. If you stopped smoking after you became pregnant, in which week of pregnancy did you stop?

☐☐ week of pregnancy

101. How long after you get up in the morning until you light your first cigarette?
- ☐ 5 minutes
- ☐ 6-29 minutes
- ☐ 30-60 minutes
- ☐ More than one hour

102. Do you smoke when you are ill?
- ☐ No
- ☐ Yes

103. Do you smoke more often during the first few hours after you wake up than you do during the rest of the day?
- ☐ No
- ☐ Yes

104. If you have used other kinds of nicotine indicate which and when you used them.

	Before pregnancy	During pregnancy
Chewing tobacco/snuff	☐	☐
Nicotine chewing gum	☐	☐
Nicotine adhesive patch	☐	☐
Nicotine inhaler	☐	☐

105. What was your fluid consumption (number of cups/glasses) per day before and during pregnancy? (1 mug = 2 cups, 1 small plastic bottle (0.5 litre) = 4 cups, 1 large plastic bottle (1.5 litres) = 12 cups)

	Number of cups/glasses		
	Before pregnancy	Now	Decaffeinated (Enter a cross)
1 Filter coffee	☐☐	☐☐	☐
2 Instant coffee	☐☐	☐☐	☐
3 Boiled coffee	☐☐	☐☐	☐
4 Tea	☐☐	☐☐	☐
5 Herbal tea	☐☐	☐☐	☐
6 Coca Cola/Pepsi etc. . . .	☐☐	☐☐	☐
7 Other fizzy drinks	☐☐	☐☐	☐
8 Diet Coca Cola/Pepsi .	☐☐	☐☐	☐
9 Other diet fizzy drinks .	☐☐	☐☐	☐
10 Tap water	☐☐	☐☐	☐
11 Bottled water	☐☐	☐☐	☐

	Before pregnancy	Now	Ecological (Enter a cross)
12 Juice/squash	☐☐	☐☐	☐
13 Diet juice/squash	☐☐	☐☐	☐
14 Milk (skim, low fat, whole)	☐☐	☐☐	☐
15 Yogurt, all types	☐☐	☐☐	☐
16 Yogurt/active Lactobacillus	☐☐	☐☐	☐
17 Other type of cultured milk - Kefir	☐☐	☐☐	☐
18 Other	☐☐	☐☐	☐

106. Have you used any of the following substances?

	Never	Previously	Last month before pregnancy	During pregnancy
1 Hash	☐	☐	☐	☐
2 Amphetamine	☐	☐	☐	☐
3 Ecstasy	☐	☐	☐	☐
4 Cocaine	☐	☐	☐	☐
5 Heroin	☐	☐	☐	☐

107. Have you ever consumed alcohol?

☐ No (proceed to question 117)
☐ Yes

Alcohol units are used to compare the different types of alcoholic beverages. 1 alcohol unit (= 1.5 cl. pure alcohol) is equivalent to:

1 bottle/can energy drink or cider
1 glass (1/3 litre) of beer
1 wine glass red or white wine
1 sherry glass sherry or fortified wine
1 snaps glass spirits or liqueur

108. How often did you consume alcohol in the 3 months before you became pregnant and how often do you consume alcohol during the pregnancy?

	Last 3 months before pregnancy	During pregnancy
1 Approximately 6-7 times a week	☐	☐
2 Approximately 4-5 times a week	☐	☐
3 Approximately 2-3 times a week	☐	☐
4 Approximately once a week	☐	☐
5 Approximately 1-3 times a month	☐	☐
6 Less than once a month	☐	☐
7 Never	☐	☐

109. What type of alcohol do you usually drink? (Fill in one or several boxes.)

1 Light beer ☐
2 Beer ☐
3 Red wine ☐
4 White wine ☐
5 Low alcohol sodas ☐
6 Fortified wines (sherry, port, Madeira) ☐
7 Spirits (vodka, gin, snaps, cognac, whisky, liqueur) ☐

110. Did you drink 5 units or more at least once during the last 3 months before pregnancy or during pregnancy?

	Last 3 months before pregnancy	During pregnancy
1 Several times per week	☐	☐
2 Once a week	☐	☐
3 1-3 times a month	☐	☐
4 Less than once a month	☐	☐
5 Never	☐	☐

111. How many units of alcohol do you usually drink when you consume alcohol?

	Last 3 months before pregnancy	During pregnancy
10 or more	☐	☐
7-9	☐	☐
5-6	☐	☐
3-4	☐	☐
1-2	☐	☐
Less than 1	☐	☐

112. How many units of alcohol do you have to drink before you feel any effect?

☐☐ units

113. Have other people irritated you or hurt your feelings by criticising how much you drink?

☐ No
☐ Yes

114. Have you ever felt that you ought to drink less alcohol?

☐ No
☐ Yes

115. Have you ever drunk alcohol in the morning to calm your nerves or to get rid of a hangover?

☐ No
☐ Yes

116. Have you ever experienced any of the following problems during the last year in relation to your alcohol consumption?

	Never	Once	Several times
Argued with or had negative feelings for a family member	☐	☐	☐
Suddenly found yourself somewhere without knowing how you got there	☐	☐	☐
Been absent from work or school	☐	☐	☐
Fainted or passed out suddenly	☐	☐	☐
Had a sad period	☐	☐	☐

Weight and weight control

117. Do you think you were overweight just before this pregnancy?

☐ Yes, a lot
☐ Yes, a little
☐ No

118. Are you worried about putting on more weight than necessary during this pregnancy?

☐ Yes, very worried
☐ Somewhat worried
☐ No, not especially worried

119. Has anyone said that you were too thin while you felt that you were overweight during the last 2 years?

☐ Yes, often
☐ Yes, occasionally
☐ No

120. Have you ever felt that you lost control while eating and were not able to stop before you have eaten far too much?

	Last 6 months before this pregnancy	Now
No	☐	☐
Infrequently	☐	☐
Yes, at least once a week	☐	☐

121. Have you ever used any of the following methods to control your weight?

	Last 6 months before this pregnancy		Now	
	At least once a week	Seldom/ Never	At least once a week	Seldom/ Never
Vomiting	☐	☐	☐	☐
Laxatives	☐	☐	☐	☐
Fasting	☐	☐	☐	☐
Hard physical exercise	☐	☐	☐	☐

122. Is it important for your self-image that you maintain a certain weight?

☐ Yes, very important
☐ Yes, quite important
☐ No, not especially important

Physical activity

123. How often do you exercise? (Fill in each line for both before and during this pregnancy.)

	Last 3 months before this pregnancy: Never	1-3 times a month	1 time a week	2 times a week	3 or more times a week	During this pregnancy: Never	1-3 times a month	1 time a week	2 times a week	3 or more times a week
1 Walking	☐	☐	☐	☐	☐	☐	☐	☐	☐	☐
2 Brisk walking	☐	☐	☐	☐	☐	☐	☐	☐	☐	☐
3 Running/jogging/orienteering	☐	☐	☐	☐	☐	☐	☐	☐	☐	☐
4 Bicycling	☐	☐	☐	☐	☐	☐	☐	☐	☐	☐
5 Training studio/weight training	☐	☐	☐	☐	☐	☐	☐	☐	☐	☐
6 Special gymnastics/aerobics for pregnant women	☐	☐	☐	☐	☐	☐	☐	☐	☐	☐
7 Aerobics/gymnastics/dance without running and jumping	☐	☐	☐	☐	☐	☐	☐	☐	☐	☐
8 Aerobics/gymnastics/dance with running and jumping	☐	☐	☐	☐	☐	☐	☐	☐	☐	☐
9 Dancing (swing/rock/folk)	☐	☐	☐	☐	☐	☐	☐	☐	☐	☐
10 Skiing	☐	☐	☐	☐	☐	☐	☐	☐	☐	☐
11 Ball sports	☐	☐	☐	☐	☐	☐	☐	☐	☐	☐
12 Swimming	☐	☐	☐	☐	☐	☐	☐	☐	☐	☐
13 Riding	☐	☐	☐	☐	☐	☐	☐	☐	☐	☐
14 Other	☐	☐	☐	☐	☐	☐	☐	☐	☐	☐

124. How often do you do exercises for the following muscle groups? (Fill in each line for both before and during this pregnancy.)

	Last 3 months before pregnancy: Never	1-3 times a month	1 time a week	2 times a week	3 or more times a week	During pregnancy: Never	1-3 times a month	1 time a week	2 times a week	3 or more times a week
Abdominal muscles	☐	☐	☐	☐	☐	☐	☐	☐	☐	☐
Back muscles	☐	☐	☐	☐	☐	☐	☐	☐	☐	☐
Pelvic floor muscles (muscles around the vagina, urethra, anus)	☐	☐	☐	☐	☐	☐	☐	☐	☐	☐

125. How often are you so physically active in your leisure and/or at work that you get out of breath or sweat?

	Last 3 months before this pregnancy: Leisure	At work	During this pregnancy: Leisure	At work
Never	☐	☐	☐	☐
Less than once a week	☐	☐	☐	☐
Once a week	☐	☐	☐	☐
2 times a week	☐	☐	☐	☐
3-4 times a week	☐	☐	☐	☐
5 times a week or more	☐	☐	☐	☐

A little more about yourself and how you are keeping now

126. Do you agree or disagree with the following statements? (Fill in only one box in each line.)

	Disagree completely	Disagree	Disagree somewhat	Don't agree or disagree	Agree somewhat	Agree	Agree completely
My life is largely what I wanted it to be	☐	☐	☐	☐	☐	☐	☐
My life is very good	☐	☐	☐	☐	☐	☐	☐
I am satisfied with my life	☐	☐	☐	☐	☐	☐	☐
To date, I have achieved what is important for me in my life	☐	☐	☐	☐	☐	☐	☐
If I could start all over, there is very little I would do differently	☐	☐	☐	☐	☐	☐	☐

127. How do these statements describe your relationship? (Only answer if you have a partner.) (Fill in only one box in each line.)

	Agree Completely	Agree	Agree somewhat	Disagree somewhat	Disagree	Disagree completely
My husband/partner and I have a close relationship	☐	☐	☐	☐	☐	☐
My partner and I have problems in our relationship	☐	☐	☐	☐	☐	☐
I am very happy in my relationship	☐	☐	☐	☐	☐	☐
My partner is usually understanding	☐	☐	☐	☐	☐	☐
I often think about ending our relationship	☐	☐	☐	☐	☐	☐
I am satisfied with my relationship with my partner	☐	☐	☐	☐	☐	☐
We often disagree about important decisions	☐	☐	☐	☐	☐	☐
I have been lucky in my choice of a partner	☐	☐	☐	☐	☐	☐
We agree about how children should be raised	☐	☐	☐	☐	☐	☐
I think my partner is satisfied with our relationship	☐	☐	☐	☐	☐	☐

128. Do you have anyone other than your husband/partner you can ask for advice in a difficult situation?

- ☐ No
- ☐ Yes 1-2 people
- ☐ Yes more than 2 people

129. How often do you meet or talk on the telephone with your family (other than those you live with) or close friends?

- ☐ Once a month or less
- ☐ 2-8 times a month
- ☐ More than twice a week

130. Do you often feel lonely?

- ☐ Almost never
- ☐ Seldom
- ☐ Sometimes
- ☐ Usually
- ☐ Almost always

131. Have you been bothered by any of the following during the last two weeks? (Enter a cross for each line.)

	Not bothered	A little bothered	Quite bothered	Very bothered
Feeling fearful	☐	☐	☐	☐
Nervousness or shakeiness inside	☐	☐	☐	☐
Feeling hopeless about the future	☐	☐	☐	☐
Feeling blue	☐	☐	☐	☐
Worrying too much about things	☐	☐	☐	☐

132. Have you ever in your adult life been slapped, hit, kicked or bothered in any way physically? (fill in one or several boxes)

	During this pregnancy	Last 6 months before pregnancy	Earlier
No	☐	☐	☐
Yes	☐	☐	☐
Don't remember	☐	☐	☐

133. Have you ever been pressured or forced to have sexual intercourse? (Fill in one or several boxes.)

	During this pregnancy	Last 6 months before pregnancy	Earlier
No, never	☐	☐	☐
Yes, pressured	☐	☐	☐
Yes, forced with violence	☐	☐	☐
Yes, raped	☐	☐	☐

134. How do you feel about yourself? (Enter a cross for each line.)

	Agree completely	Agree	Disagree	Disagree completely
I have a positive attitude toward myself	☐	☐	☐	☐
I feel completely useless at times	☐	☐	☐	☐
I feel that I do not have much to be proud about	☐	☐	☐	☐
I feel that I am a valuable person, as good as anyone else	☐	☐	☐	☐

135. Have you ever experienced the following for a continuous period of 2 weeks or more? (Fill in each line.)

	No	Yes
Felt depressed, sad	☐	☐
Had problems with appetite or eaten too much	☐	☐
Been bothered by feeling weaker or a lack of energy . .	☐	☐
Really blamed yourself and felt worthless	☐	☐
Had problems with concentration or had problems making decisions	☐	☐
Had at least 3 of the problems named above simultaneously	☐	☐

136. If you have had 3 or more of these problems at the same time, how many weeks did the longest period last?

☐☐ weeks

137. Was there a particular reason for this?

- ☐ No, no particular reason
- ☐ Yes (e.g. death, divorce, miscarriage, accident)

We would be grateful if you would write anything else you would like to tell us about this pregnancy or previous births/pregnancies that are not addressed in this questionnaire on the next page.

Comments

Have you remembered to fill in the date on which you completed the questionnaire on page 1?

Thank you very much for your help!

Please return the completed questionnaire in the stamped addressed envelope provided.

Avd. for medisinsk fødselsregister
Kalfarveien 31
5018 Bergen

Bording
