# Supplementary material for: Does progestin-only contraceptive use after pregnancy affect recovery from pelvic girdle pain? A prospective population study
Source: PLoS One. 2017 Sep 11;12(9):e0184071. doi: 10.1371/journal.pone.0184071 (PMC5593199; doi:10.1371/journal.pone.0184071)
Supplement: S4 Questionnaire — (PDF) [file pone.0184071.s004.pdf]

den norske Mor & barn undersøkelsen

Spørreskjema 1

Skjemaet skal leses av en maskin. Det er derfor viktig at du legger vekt på følgende ved utfyllingen:

- Bruk blå eller sort kulepenn.
- I de små avkrysningsboksene setter du *ett kryss* for det svaret som du mener passer best, slik: ☒
- Hvis du mener at du har satt kryss i feil boks, kan du rette det ved å fylle boksen helt, slik ■
- I de store, grønne boksene skriver du *tall* eller *store bokstaver.*

Det er viktig at du bare skriver i det hvite feltet i boksene, slik:

Tall: 0 1 2 3 4 5 6 7 8 9 Bokstaver: A B C D

- Tallboksene har to eller flere ruter. Når du skriver et ett-sifret tall bruker du den høyre ruten. Eksempel: 5 skrives slik [ ][5]
- Flere steder i skjemaet ber vi om at du angir svaret i forhold til antall svangerskapsuker. *Eksempel: Hvis du skal angi noe som skjedde 5 uker etter siste menstruasjon, krysser du av for uke 5.*
- Spesielle opplysninger som *f.eks. medikamenter og yrke* skriver du fritt inne i boksene eller på de åpne linjene. *Vennligst skriv tydelig med STORE BOKSTAVER.*
- Husk å fylle ut dato for utfylling av skjemaet

Så snart du har fylt ut dette skjemaet, ber vi om at du sender det tilbake til oss i den vedlagte, frankerte svarkonvolutten.

Oppgi dag, måned og år for utfyllingen av skjemaet [ ][ ] dag [ ][ ] måned [ ][ ][ ][ ] år *(skriv årstall med 4 tall f.eks. 2000)*

Menstruasjon

1. Hvor gammel var du da du fikk din første menstruasjon?

[ ][ ] år

2. Hvor lang tid går det vanligvis mellom to menstruasjoner, dvs. fra første dag i en menstruasjon til første dag i den neste?

[ ][ ] dager

3. Pleier du å være nedtrykt (deprimert) eller irritabel før menstruasjonen?

☐ Nei
☐ Ja, men ubetydelig
☐ Ja, merkbart
☐ Ja, plagsomt mye

4. Hvis ja, forsvinner denne følelsen etter at menstruasjonen er kommet i gang?

☐ Nei
☐ Ja

5. Hadde du regelmessige menstruasjoner det siste året før du ble gravid?

☐ Nei
☐ Ja

6. Har du i løpet av det siste året før du ble gravid mistet menstruasjonen i mer enn tre måneder?

☐ Nei
☐ Ja, på grunn av tidligere svangerskap
☐ Ja, på grunn av andre forhold

7. Oppgi datoen for første blødningsdag i din siste menstruasjon.

[ ][ ] dag [ ][ ] måned [ ][ ][ ][ ] år

8. Kom din siste menstruasjon til ventet tid?

☐ Nei
☐ Ja

9. Er du sikker eller usikker på datoen for første blødningsdag i din siste menstruasjon?

☐ Sikker
☐ Usikker

10. Hvordan var varighet, blødningsmengde og smerter i din siste menstruasjon?

	Som vanlig	Mer enn vanlig	Mindre enn vanlig
Varighet	☐	☐	☐
Blødningsmengde	☐	☐	☐
Smerter	☐	☐	☐

Spørreskjema 1E - MB - 30.000 - 1106

Prevensjon og graviditet

11. Har du/dere noen gang det siste året brukt følgende metoder for å unngå graviditet? *(Sett eventuelt flere kryss.)*

- ☐ Kondom
- ☐ Pessar
- ☐ Kobberspiral
- ☐ Hormonspiral
- ☐ Hormonsprøyte
- ☐ Mini-piller
- ☐ P-piller
- ☐ Skum, stikkpille, krem
- ☐ Sikre perioder
- ☐ Avbrutt samleie
- ☐ Ingen slike metoder
- ☐ Annet______________________________

12. Hvis du har brukt p-piller/mini-piller, hvor lenge til sammen har du brukt dem?

	P-piller	Mini-piller
Mindre enn 1 år	☐	☐
1-3 år	☐	☐
4-6 år	☐	☐
7-9 år	☐	☐
10 år eller mer	☐	☐

13. Hvis du har brukt p-piller/mini-piller, hvor gammel var du da du første gang brukte disse?

☐☐ år

14. Brukte du p-piller/mini-piller de siste 4 månedene før du ble gravid denne gangen?

- ☐ Nei
- ☐ Ja

15. Hvis ja, hvor lang tid før siste menstruasjon sluttet du med p-piller/mini-piller?

☐☐ uker

16. Var dette svangerskapet planlagt?

- ☐ Nei
- ☐ Ja

17. Hvis ja, hvor mange måneder hadde dere regelmessig samleie uten prevensjon før du ble gravid?

- ☐ mindre enn 1 måned
- ☐ 1-2 måneder
- ☐ 3 måneder eller mer

☐☐ måneder hvis mer enn 3 måneder

18. Ble du gravid selv om du eller din partner brukte prevensjon?

- ☐ Nei *(Gå til spørsmål 21.)*
- ☐ Ja

19. Hvis ja, hvilken type? *(Sett eventuelt flere kryss.)*

- ☐ Kondom
- ☐ Pessar
- ☐ Kobberspiral
- ☐ Hormonspiral
- ☐ Hormonsprøyte
- ☐ Mini-piller
- ☐ P-piller
- ☐ Skum, stikkpille, krem
- ☐ Sikre perioder
- ☐ Avbrutt samleie
- ☐ Annet______________________________

20. Hvis du hadde spiral da du ble gravid, er den fjernet nå?

- ☐ Nei
- ☐ Ja

21. Hvor lenge har du og barnets far hatt et seksuelt forhold?

☐☐ måneder eller ☐☐ år

22. Hvor ofte har du hatt samleie i løpet av de siste fire ukene før du ble gravid og i de siste fire ukene nå?

	Før svangerskap	Nå
Daglig	☐	☐
5–6 ganger i uken	☐	☐
3–4 ganger i uken	☐	☐
1–2 ganger i uken	☐	☐
1–2 ganger hver 14. dag	☐	☐
Sjeldnere	☐	☐
Ingen ganger	☐	☐

23. Har du noen gang vært behandlet for ufrivillig barnløshet?

- ☐ Nei
- ☐ Ja

24. Hvis ja, var det i forbindelse med dette svangerskapet eller tidligere svangerskap og hva slags behandling var det?
(Sett eventuelt flere kryss.)

	Tidligere svanger-skap	Dette svanger-skap
Operasjon på eggledere	☐	☐
Annen form for operasjon	☐	☐
Medisiner mot endometriose	☐	☐
Hormonbehandling	☐	☐
Inseminasjon (innsprøyting av sæd)	☐	☐
Prøverørsmetoden	☐	☐
Annet	☐	☐

25. Har du fått informasjon om muligheten for å få utført fostervannsprøve?

- ☐ Nei
- ☐ Ja

26. Hva var blodtrykket ditt ved første svangerskapskontroll?
(Se i helsekortet ditt.)

☐☐☐ / ☐☐☐ *Eks.* 1 5 0 / 9 5

27. Hvor mye veide du da du ble gravid, og hvor mye veier du nå (i hele kg)?

Da jeg ble gravid: ☐☐☐ kg Nå: ☐☐☐ kg

28. Hvor høy er du?

☐☐☐ cm

29. Hvor høy (ca.) er barnets far?

☐☐☐ cm

30. Hvor mye (ca.) veier barnets far (i hele kg)?

☐☐☐ kg

Tidligere svangerskap

31. Har du vært gravid tidligere? *(Dette gjelder også svangerskap som endte med abort eller dødfødsel.)*

☐ Nei *(Gå til spørsmål 36.)*
☐ Ja

32. Hvis ja, kryss av for alle tidligere svangerskap. Ta også med svangerskap som endte med abort eller dødfødsel, eller der svangerskapet var utenfor livmoren. Oppgi årstall for svangerskapsstart, hvor mange kilo du la på deg i løpet av svangerskapet og antall måneder du ammet hvert barn. Kryss også av om du røykte i tidligere svangerskap.

Svanger-skaps-nummer	Årstall for svanger skaps-start	Levende født barn	Spontan-aborter/ dødfødsler	Frem-kalt abort	Svanger-skap utenfor livmoren	Svangerskaps-uke for aborten/ dødfødselen	Antall måneder med amming	Vektøkning i svanger-skapet (i hele kg)	Røykte i svanger-skapet
1		☐	☐	☐	☐				☐
2		☐	☐	☐	☐				☐
3		☐	☐	☐	☐				☐
4		☐	☐	☐	☐				☐
5		☐	☐	☐	☐				☐
6		☐	☐	☐	☐				☐
7		☐	☐	☐	☐				☐
8		☐	☐	☐	☐				☐
9		☐	☐	☐	☐				☐
10		☐	☐	☐	☐				☐

33. Har du hatt noen av følgende plager i tidligere svangerskap?
(Kryss av for hver linje.)

		Nei	Ja
1	Bekkenløsning som førte til sykemelding	☐	☐
2	Bekkenløsning som gjorde det nødvendig med sengeleie	☐	☐
3	Mye plaget av kvalme og oppkast	☐	☐
4	Svangerskapsforgiftning	☐	☐
5	Svangerskapsdiabetes	☐	☐
6	Sukker i urinen	☐	☐
7	Mye plaget av ufrivillig urinlekkasje	☐	☐

34. Hvis du hadde bekkenløsning i tidligere svangerskap som gjorde det nødvendig med sengeleie eller sykemelding, når begynte plagene?

☐ måneder etter påbegynt svangerskap

35. Når sluttet plagene?

☐☐ måneder etter fødselen

☐ har hatt vedvarende plager

Sykdommer og helseplager i dette svangerskapet

36. Har du hatt en eller flere blødninger fra skjeden i løpet av dette svangerskapet?

☐ Nei
☐ Ja

37. Hvis ja, beskriv den første og den siste blødningen. Angi dato da blødningen startet, hvor mange dager det varte og hvor mye du blødde?

	Dato da blødningen startet (dag / måned / år)	Blødningen varte i antall dager	Kryss av for blødningsmengde *(sporblødning betyr noen dråper)*
Første blødning	☐☐ ☐☐ ☐☐☐☐	☐☐	☐ Sporblødning ☐ Mer enn sporblødning ☐ Klumper
Siste blødning	☐☐ ☐☐ ☐☐☐☐	☐☐	☐ Sporblødning ☐ Mer enn sporblødning ☐ Klumper

Hvis mer enn to blødningsepisoder angi antall ☐☐

38. Har du opplevd noen av følgende sykdommer eller helseplager i dette svangerskapet? Hvis du har brukt medisiner i forbindelse med disse plagene, oppgi navn på medisin, i hvilke svangerskapsuker du brukte medisiner og antall dager du brukte dem. *(Dette gjelder alle typer medikamenter, både faste og ikke-faste og naturmedisiner. Ikke før inn vitaminer og kosttilskudd - disse spør vi om senere i skjemaet.)*

Sykdommer / helseplager i dette svangerskapet					Bruk av medisiner i dette svangerskap					
	I svangerskapsuker					I svangerskapsuker				Antall dager brukt
Sykdom/helseplage	0-4	5-8	9-12	13+	Navn på medisiner du brukte	0-4	5-8	9-12	13+	
1 Bekkenløsning	☐	☐	☐	☐		☐	☐	☐	☐	
2 Magesmerter	☐	☐	☐	☐		☐	☐	☐	☐	
3 Vondt i ryggen	☐	☐	☐	☐		☐	☐	☐	☐	
4 Nakke-/skuldersmerter	☐	☐	☐	☐		☐	☐	☐	☐	
5 Kvalme	☐	☐	☐	☐		☐	☐	☐	☐	
6 Kvalme med brekninger/oppkast	☐	☐	☐	☐		☐	☐	☐	☐	
7 Soppinfeksjon i skjeden	☐	☐	☐	☐		☐	☐	☐	☐	
8 Skjedekatarr/uvanlig utflod	☐	☐	☐	☐		☐	☐	☐	☐	
9 Svangerskapskløe	☐	☐	☐	☐		☐	☐	☐	☐	
10 Treg mage/forstoppelse	☐	☐	☐	☐		☐	☐	☐	☐	
11 Diaré/omgangssyke	☐	☐	☐	☐		☐	☐	☐	☐	
12 Uvanlig tretthet/søvnighet	☐	☐	☐	☐		☐	☐	☐	☐	
13 Søvnproblemer	☐	☐	☐	☐		☐	☐	☐	☐	
14 Halsbrann/sure oppstøt	☐	☐	☐	☐		☐	☐	☐	☐	
15 Hevelse i kroppen (ødem)	☐	☐	☐	☐		☐	☐	☐	☐	
16 Feber med utslett	☐	☐	☐	☐		☐	☐	☐	☐	
17 Feber over 38,5oC	☐	☐	☐	☐		☐	☐	☐	☐	
18 Forkjølelse	☐	☐	☐	☐		☐	☐	☐	☐	
19 Halsbetennelse	☐	☐	☐	☐		☐	☐	☐	☐	
20 Bihule-/ørebetennelse	☐	☐	☐	☐		☐	☐	☐	☐	
21 Influensa	☐	☐	☐	☐		☐	☐	☐	☐	
22 Lungebetennelse/bronkitt	☐	☐	☐	☐		☐	☐	☐	☐	
23 Sukker i urin	☐	☐	☐	☐		☐	☐	☐	☐	
24 Eggehvite (protein) i urin	☐	☐	☐	☐		☐	☐	☐	☐	

Tidligere og nåværende sykdommer og helseplager

39. Kryss av hvis du har eller har hatt noen av følgende sykdommer eller helseplager. Hvis du har brukt tabletter, miksturer, stikkpiller, inhalasjoner, salver osv. i forbindelse med sykdommen eller helseplager, oppgi navnet på medisinen(e) og når du brukte disse.

Sykdommer / helseplager			Bruk av medisiner						
Sykdom / helseplage	Før svanger-skapet	I svanger-skapet	Navn på medisiner	Siste 6 mnd. før svanger-skapet	I svangerskapsuke 0-4	5-8	9-12	13+	Antall dager brukt
**Astma / Allergi / Hud**									
1 Astma	☐	☐		☐	☐	☐	☐	☐	
2 Høysnue, pollenallergi	☐	☐		☐	☐	☐	☐	☐	
3 Dyrehårsallergi	☐	☐		☐	☐	☐	☐	☐	
4 Annen allergi	☐	☐		☐	☐	☐	☐	☐	
5 Atopisk eksem *(ofte kalt barneeksem)*	☐	☐		☐	☐	☐	☐	☐	
6 Elveblest *(urticaria)*	☐	☐		☐	☐	☐	☐	☐	
7 Psoriasis	☐	☐		☐	☐	☐	☐	☐	
8 Annen eksem	☐	☐		☐	☐	☐	☐	☐	
9 Munnsår *(herpes)*	☐	☐		☐	☐	☐	☐	☐	
10 Akne/kviser *(alvorlig)*	☐	☐		☐	☐	☐	☐	☐	
**Diabetes / Sukkersyke**									
11 Diabetes behandlet med insulin	☐	☐		☐	☐	☐	☐	☐	
12 Diabetes ikke behandlet med insulin	☐	☐		☐	☐	☐	☐	☐	
**Hjerte / Blod / Stoffskifte / Blodkar**									
13 Medfødt hjertefeil	☐	☐		☐	☐	☐	☐	☐	
14 Annen hjerte-/karsykdom	☐	☐		☐	☐	☐	☐	☐	
15 Forhøyet kolesterol	☐	☐		☐	☐	☐	☐	☐	
16 For høyt blodtrykk	☐	☐		☐	☐	☐	☐	☐	
17 For høyt eller for lavt stoffskifte	☐	☐		☐	☐	☐	☐	☐	
18 Anemi/lav blodprosent	☐	☐		☐	☐	☐	☐	☐	
19 B-12-/folat/folsyremangel	☐	☐		☐	☐	☐	☐	☐	
**Mage / Tarm**									
20 Hepatitt/leverbetennelse	☐	☐		☐	☐	☐	☐	☐	
21 Gallestein	☐	☐		☐	☐	☐	☐	☐	
22 Magesår	☐	☐		☐	☐	☐	☐	☐	
23 Crohns sykdom / Ulcerøs colitt	☐	☐		☐	☐	☐	☐	☐	
24 Cøliaki	☐	☐		☐	☐	☐	☐	☐	
25 Annen mage-/tarmplager	☐	☐		☐	☐	☐	☐	☐	
**Muskel / Skjelett / Bindevev**									
26 Leddgikt *(revmatoid artritt)*, Bekhterevs sykdom	☐	☐		☐	☐	☐	☐	☐	

Sykdommer / helseplager			Bruk av medisiner						
Sykdom / helseplage	Før svanger-skapet	I svanger-skapet	Navn på medisiner	Siste 6 mnd. før svanger-skapet	I svangerskapsuke 0-4	5-8	9-12	13+	Antall dager brukt
27 Lupus *(SLE)*	☐	☐		☐	☐	☐	☐	☐	
28 Isjias	☐	☐		☐	☐	☐	☐	☐	
29 Fibromyalgi	☐	☐		☐	☐	☐	☐	☐	
**Underlivet / Urinveier**									
30 Betennelse i eggstokker/ledere	☐	☐		☐	☐	☐	☐	☐	
31 Endometriose	☐	☐		☐	☐	☐	☐	☐	
32 Nedfall av livmor	☐	☐		☐	☐	☐	☐	☐	
33 Cyste på eggstokk	☐	☐		☐	☐	☐	☐	☐	
34 Muskelknuter på livmor	☐	☐		☐	☐	☐	☐	☐	
35 Celleforandringer på livmorhals	☐	☐		☐	☐	☐	☐	☐	
36 Herpes	☐	☐		☐	☐	☐	☐	☐	
37 Kjønnsvorter/kondylomer	☐	☐		☐	☐	☐	☐	☐	
38 Gonoré	☐	☐		☐	☐	☐	☐	☐	
39 Chlamydia	☐	☐		☐	☐	☐	☐	☐	
40 Nyrestein	☐	☐		☐	☐	☐	☐	☐	
41 Nyrebekkenbetennelse	☐	☐		☐	☐	☐	☐	☐	
42 Urinveisinfeksjon *(blærekatarr)*	☐	☐		☐	☐	☐	☐	☐	
43 Urinlekkasje	☐	☐		☐	☐	☐	☐	☐	
**Andre sykdommer eller helseplager**									
44 Anorexi/bulemi/spiseforstyrrelser	☐	☐		☐	☐	☐	☐	☐	
45 Migrene	☐	☐		☐	☐	☐	☐	☐	
46 Annen hodepine	☐	☐		☐	☐	☐	☐	☐	
47 Epilepsi	☐	☐		☐	☐	☐	☐	☐	
48 Multippel sklerose	☐	☐		☐	☐	☐	☐	☐	
49 Cerebral parese	☐	☐		☐	☐	☐	☐	☐	
50 Kreft	☐	☐		☐	☐	☐	☐	☐	
51 Depresjon	☐	☐		☐	☐	☐	☐	☐	
52 Angst	☐	☐		☐	☐	☐	☐	☐	
53 Annen sykdom eller helseplage	☐	☐		☐	☐	☐	☐	☐	

Hvilken: ____________________

40. Har du selv en medfødt misdannelse/fosterskade?

☐ Nei
☐ Ja

41. Hvis ja, hvilken?______________________________

42. Blør du for tiden fra tannkjøttet når du pusser tennene?

☐ Nei, sjelden eller aldri
☐ Ja, av og til
☐ Ja, ofte
☐ Ja, nesten alltid

43. Hvis du hadde diabetes/sukkersyke før du ble gravid, hva var måleresultatet for ditt langtids blodsukker (HbA1c) ved siste måling før dette svangerskapet?

☐ Mindre enn 7,5
☐ 7,5 - 12
☐ Mer enn 12
☐ Vet ikke

Andre medisiner

44. Har du brukt andre medisiner som du ikke har nevnt tidligere? Hvis ja, oppgi navn og når du har tatt disse i tabellen nedenfor.

Navn på medisiner *(f.eks. Valium, Rohypnol, Paracet)*	Bruk av medisiner: Siste 6 mnd. før svangerskapet	I svangerskapsuker 0-4	5-8	9-12	13+	Antall dager brukt
__________	☐	☐	☐	☐	☐	
__________	☐	☐	☐	☐	☐	
__________	☐	☐	☐	☐	☐	
__________	☐	☐	☐	☐	☐	
__________	☐	☐	☐	☐	☐	

Vitaminer, mineraler og kosttilskudd

45. Har du brukt vitaminer, mineraler eller annet kosttilskudd i svangerskapet eller siste halvår før svangerskapet?

☐ Nei, *(Gå til spørsmål 49.)*
☐ Ja

46. Hvis ja, vennligst finn frem eske/glass og bruk innholdslisten til å fylle ut tabellen nedenfor.

(F.eks. hvis du har tatt tran hver dag det siste halvåret før svangerskapet, skal du sette ett kryss for hver periode under «Når» (dvs. 7 kryss) og ett kryss for «daglig» under «Hvor ofte»).

	Når har du brukt tilskudd? Siste halvår før svangerskapet			I svangerskapet				I den perioden du brukte tilskudd, omtrent hvor ofte har du brukt dette?		
	26-9 uke	8-5 uke	4-0 uke	0-4 uke	5-8 uke	9-12 uke	13+ uke	Daglig	4-6 ganger pr. uke	1-3 ganger pr. uke
1 Folat /folsyre	☐	☐	☐	☐	☐	☐	☐	☐	☐	☐
2 Vitamin B1 (Thiamin)	☐	☐	☐	☐	☐	☐	☐	☐	☐	☐
3 Vitamin B2 (Riboflavin)	☐	☐	☐	☐	☐	☐	☐	☐	☐	☐
4 Vitamin B6 (Pyridoksin)	☐	☐	☐	☐	☐	☐	☐	☐	☐	☐
5 Vitamin B12 (Cyanokobalamin)	☐	☐	☐	☐	☐	☐	☐	☐	☐	☐
6 Niacin	☐	☐	☐	☐	☐	☐	☐	☐	☐	☐
7 Pantotensyre (pantotenat)	☐	☐	☐	☐	☐	☐	☐	☐	☐	☐
8 Biotin	☐	☐	☐	☐	☐	☐	☐	☐	☐	☐
9 Vitamin C	☐	☐	☐	☐	☐	☐	☐	☐	☐	☐
10 Vitamin A	☐	☐	☐	☐	☐	☐	☐	☐	☐	☐
11 Vitamin D	☐	☐	☐	☐	☐	☐	☐	☐	☐	☐
12 Vitamin E	☐	☐	☐	☐	☐	☐	☐	☐	☐	☐
13 Jern	☐	☐	☐	☐	☐	☐	☐	☐	☐	☐
14 Kalk/kalsium	☐	☐	☐	☐	☐	☐	☐	☐	☐	☐
15 Jod	☐	☐	☐	☐	☐	☐	☐	☐	☐	☐
16 Sink	☐	☐	☐	☐	☐	☐	☐	☐	☐	☐
17 Selen	☐	☐	☐	☐	☐	☐	☐	☐	☐	☐
18 Kobber	☐	☐	☐	☐	☐	☐	☐	☐	☐	☐
19 Krom	☐	☐	☐	☐	☐	☐	☐	☐	☐	☐
20 Magnesium	☐	☐	☐	☐	☐	☐	☐	☐	☐	☐
21 Tran	☐	☐	☐	☐	☐	☐	☐	☐	☐	☐
22 Omega-3 fettsyre	☐	☐	☐	☐	☐	☐	☐	☐	☐	☐

47. Oppgi fullstendig produktnavn på alle de vitaminer eller kosttilskudd du bruker. Ta også med naturmedisiner og slankepulver.
(Skriv tydelig med blokkbokstaver da det skal leses maskinelt.)

F.eks V I T A P L E X M E D J E R N

1

2

3

4

5

6

48. Hvis du bruker multivitaminer (med eller uten mineraler), inneholder disse folat/folsyre?
- ☐ Ja
- ☐ Nei
- ☐ Vet ikke

Sivilstand og utdannelse

49. Hvilken sivilstand har du nå?
- ☐ Gift
- ☐ Samboer
- ☐ Enslig
- ☐ Skilt/separert
- ☐ Enke
- ☐ Annet

50. Hvilken utdannelse har du og barnets far? *(Sett kun ett kryss for den høyeste utdannelsen dere har fullført. Kryss også av for den utdannelsen dere eventuelt holder på med.)*

	Deg		Barnets far	
	Fullført	Holder på med	Fullført	Holder på med
1 9-årig grunnskole	☐	☐	☐	☐
2 1-2-årig videregående	☐	☐	☐	☐
3 Videregående yrkesfaglig	☐	☐	☐	☐
4 3-årig videregående allmennfaglig, gymnas	☐	☐	☐	☐
5 Distriktshøyskole, universitet inntil 4 år *(cand. mag., sykepleier, lærer, ingeniør)*	☐	☐	☐	☐
6 Universitet, høyskole, mer enn 4 år *(hovedfag, embetseksamen)*	☐	☐	☐	☐
7 Annen utdannelse	☐	☐	☐	☐

Arbeid og fritid

51. Hva var arbeidssituasjonen for deg og barnets far da du ble gravid? *(Sett eventuelt flere kryss.)*

	Deg	Barnets far
1 Skoleelev/student	☐	☐
2 Hjemmeværende	☐	☐
3 Yrkespraksis/lærling	☐	☐
4 Militærtjeneste	☐	☐
5 Arbeidssøkende/permittert	☐	☐
6 Attføring/ufør	☐	☐
7 Ansatt i offentlig virksomhet	☐	☐
8 Ansatt i privat virksomhet	☐	☐
9 Selvstendig næringsdrivende	☐	☐
10 Familiemedlem uten fast lønn i familiebedrift *(f.eks. gårdsbruk, forretning)*	☐	☐
11 Annet	☐	☐

52. Hadde du en ekstrajobb (lønnet eller ulønnet) da du ble gravid?
(f.eks. regnskapsfører, frisør, vokalist i danseband, fritidsleder)

- ☐ Nei
- ☐ Ja, beskriv: ______________________

53. Har du hatt fravær fra ditt vanlige arbeid i til sammen mer enn to uker i løpet av dette svangerskapet?

- ☐ Nei
- ☐ Ja

54. Er du fraværende fra ditt vanlige arbeid nå?

- ☐ Nei
- ☐ Ja

55. Hvis ja, hva er årsaken til fraværet? *(Sett eventuelt flere kryss.)*

- ☐ Sykemelding
- ☐ Permisjon
- ☐ Sykt barn
- ☐ Annet ______________________

56. Antall timer lønnet med arbeid vanligvis pr. uke før du ble gravid og nå?

Før svangerskapet: ☐☐,☐ timer

I svangerskapet: ☐☐,☐ timer

Spørsmål om nåværende arbeidssituasjon besvares av alle som har inntektsgivende arbeid, selv om de midlertidig er fraværende fra dette pga. sykdom, permisjon eller lignende.

	Deg	Barnets far
**57. Beskriv virksomheten på nåværende arbeidsplass eller tjenestested så nøyaktig som mulig.** *(Skriv f.eks. sykehusavdeling for barn med kreft, karosseriavdeling på verksted for dieselbiler, gårdsbruk med korn og gris, hjemmearbeid.)*		
**58. Yrke/tittel på dette arbeidsstedet?** *(Skriv f.eks. avdelingssykepleier, bilopprettter, formann, adjunkt, elev, spesialarbeider, renholdsassistent, hjemmeværende.)*		

59. Kryss av for følgende spørsmål som gjelder nåværende arbeid. *(Sett kun ett kryss for hver linje.)*

	Ja, daglig mer enn halve arbeidstiden	Ja, daglig mindre enn halve arbeidstiden	Ja, i perioder, men ikke daglig	Sjelden eller aldri
Hender det at du har så mye å gjøre at arbeidssituasjonen blir oppjaget og masete?	☐	☐	☐	☐
Må du vri eller bøye deg mange ganger i timen?	☐	☐	☐	☐
Arbeider du med hendene løftet i skulderhøyde eller høyere?	☐	☐	☐	☐
Arbeider du stående eller gående?	☐	☐	☐	☐
Kan du velge å arbeide litt raskere visse dager og litt roligere andre dager?	☐	☐	☐	☐
Er du utsatt for så mye støy eller lyder at du synes det er ubehagelig?	☐	☐	☐	☐
Er du utsatt for så mye støy eller lyder at du må heve stemmen for å snakke med andre, selv på en meters avstand?	☐	☐	☐	☐

60. Hvordan stemmer følgende beskrivelser av din arbeidssituasjon. *(Sett kun ett kryss for hver linje.)*

	Stemmer	Stemmer ganske bra	Stemmer ikke særlig bra	Stemmer ikke i det hele tatt
Jeg har fysisk tungt arbeid.	☐	☐	☐	☐
Jeg har et stressende eller masete arbeid.	☐	☐	☐	☐
Jeg lærer mye i arbeidet mitt.	☐	☐	☐	☐
Arbeidet innebærer at jeg gjør de samme tingene om og om igjen.	☐	☐	☐	☐
Arbeidet mitt krever stor arbeidsinnsats.	☐	☐	☐	☐
Jeg har muligheten til selv å bestemme hvordan arbeidet skal utføres.	☐	☐	☐	☐
Det er godt samhold på arbeidsplassen.	☐	☐	☐	☐
Jeg trives i arbeidet mitt.	☐	☐	☐	☐

61. Hvilken arbeidstidsordning har du nå? *(Sett eventuelt flere kryss.)*

- ☐ Fast dagarbeid
- ☐ Fast ettermiddags- eller kveldsarbeid
- ☐ Fast nattarbeid
- ☐ Skiftarbeid eller turnusordning
- ☐ Ingen fast ordning *(ekstrahjelp, ekstravakt, vikar o.l.)*
- ☐ Annen ordning

62. Løfter du nå når du er gravid noe som veier mer enn 10 kg?
(10 kg tilsvarer vekten av en full vannbøtte.)

	Hjemme	På arbeid
Sjelden eller aldri	☐	☐
Ja, mindre enn 20 ganger ukentlig	☐	☐
Ja, mer enn 20 ganger ukentlig	☐	☐
Ja, 10 til 20 ganger daglig	☐	☐
Ja, mer enn 20 ganger daglig	☐	☐

63. Hvor ofte har du arbeidet ved radiosender eller radar etter at du ble gravid?

- ☐ Sjelden/Aldri
- ☐ Noen ganger i uken
- ☐ Daglig
- ☐ I gjennomsnitt mer enn 1 time daglig

64. Hvor ofte snakker du i mobiltelefon?

- ☐ Sjelden/Aldri
- ☐ Noen ganger i uken
- ☐ Daglig
- ☐ I gjennomsnitt til sammen mer enn 1 time daglig

65. Varer en enkel mobiltelefonsamtale mer enn 15 minutter?

- ☐ Aldri
- ☐ Sjelden
- ☐ Ofte

66. Hvor ofte har du arbeidet ved dataskjerm, laserprinter eller kopieringsmaskin (mindre enn 2 meters avstand) etter at du ble gravid?

	Dataskjerm	Laserprinter	Kopierings-maskin
Sjelden/Aldri	☐	☐	☐
Noen ganger i uken	☐	☐	☐
Daglig	☐	☐	☐
I gjennomsnitt mer enn 1 time daglig	☐	☐	☐

67. Hvor ofte har du arbeidet ved røntgenapparat (mindre enn 2 meters avstand) etter at du ble gravid?
(Ta ikke med behandling som pasient.)

- ☐ Sjelden/Aldri
- ☐ Noen ganger i uken
- ☐ Daglig
- ☐ I gjennomsnitt mer enn 1 time daglig

68. Har du vært i kontakt med noe av følgende i fritid eller arbeid i løpet av det siste halve året? *(Kryss av for hver linje.)*

		Nei	Ja	Hvis ja, antall dager siste 1/2 året (daglig = 180 dager)	Kryss av hvis du har brukt avtrekk eller åndedrettsvern	Kryss av om du har brukt hansker
1	Blydunster, blystøv, blypartikler eller blylegeringer	☐	☐	☐☐☐	☐	☐
2	Krom, arsenikk, kadmium eller sammensetninger av disse	☐	☐	☐☐☐	☐	☐
3	Bensin eller eksos *(gjelder ikke fylling av bensin til egen bil)*	☐	☐	☐☐☐	☐	☐
4	Kvikksølvdamp, kvikksølv eller arbeid med amalgam-fyllinger *(ta ikke med beh. som pasient)*	☐	☐	☐☐☐	☐	☐
5	Desinfeksjonsmidler, midler mot skadedyr	☐	☐	☐☐☐	☐	☐
6	Plantevernmidler *(ugressmidler, insektmidler, soppmidler)*	☐	☐	☐☐☐	☐	☐
7	Oljebasert maling	☐	☐	☐☐☐	☐	☐
8	Vannbasert eller latex maling	☐	☐	☐☐☐	☐	☐
9	Malingtynnere, maling-, lakk- eller limfjerner eller andre løsemidler *(f.eks lynol, white spirit, toluen, karbontetraklorid)*	☐	☐	☐☐☐	☐	☐
10	Fargestoffer eller trykksverte i industri	☐	☐	☐☐☐	☐	☐
11	Motorolje, smøreolje eller andre typer olje	☐	☐	☐☐☐	☐	☐
12	Fotokjemikalier *(fiks eller fremkaller)*	☐	☐	☐☐☐	☐	☐
13	Stoffer i forbindelse med sveising	☐	☐	☐☐☐	☐	☐
14	Stoffer i forbindelse med lodding	☐	☐	☐☐☐	☐	☐
15	Formalin/formaldehyd	☐	☐	☐☐☐	☐	☐
16	Kjemoterapeutiske stoffer/cellegiftbehandling *(ta ikke med behandling som pasient)*	☐	☐	☐☐☐	☐	☐
17	Lystgass eller andre narkosegasser *(ta ikke med behandling som pasient)*	☐	☐	☐☐☐	☐	☐
18	Andre stoffer og forhold, beskriv ____________________	☐	☐	☐☐☐	☐	☐

69. Hvor ofte har du gått på diskotek etter at du ble gravid?

- ☐ 1-2 ganger i uken
- ☐ Sjeldnere
- ☐ Aldri

70. Har du kontakt med dyr i ditt arbeid eller din fritid?

- ☐ Nei
- ☐ Ja

71. Hvis ja, hva slags dyr og hvor ofte pr. uke er du i kontakt med dyr?

	Daglig	3-6 ganger pr. uke	1-2 ganger pr. uke	Mindre enn 1 gang pr. uke
1 Hund	☐	☐	☐	☐
2 Katt	☐	☐	☐	☐
3 Marsvin	☐	☐	☐	☐
4 Hamster	☐	☐	☐	☐
5 Kanin	☐	☐	☐	☐
6 Undulat o.l.	☐	☐	☐	☐
7 Akvariefisk	☐	☐	☐	☐
8 Ku	☐	☐	☐	☐
9 Gris	☐	☐	☐	☐
10 Sau, geit	☐	☐	☐	☐
11 Hest	☐	☐	☐	☐
12 Fjærkre	☐	☐	☐	☐
13 Annet	☐	☐	☐	☐

Bolig og husholdning

72. Hvem deler du husholdning med? *(Sett eventuelt flere kryss.)*

☐ Ektefelle/samboer
☐ Foreldre
☐ Svigerforeldre
☐ Barn
☐ Ingen
☐ Andre, beskriv____________________

73. Hvor mange personer er det i husholdningen.
(Tell med deg selv.)

Antall personer over 18 år . . . . ☐☐
Antall personer 12-18 år . . . . ☐☐
Antall personer 6-11 år . . . . ☐☐
Antall personer under 6 år . . . . ☐☐

74. Hvor mange av barna går regelmessig i barnehage?

☐ barn

75. Har du eller barnets far et annet morsmål enn norsk?

☐ Nei
☐ Ja

76. Hvis ja, hvilket morsmål?

	Deg	Barnets far
Samisk	☐	☐
Urdu	☐	☐
Engelsk	☐	☐
Annet	☐	☐

Hvis annet, hvilket?____________________

77. Har dine foreldre eller foreldre til barnets far et annet morsmål enn norsk?

☐ Nei
☐ Ja

78. Hvis ja, hvilket morsmål?

	Din egen mor	Din egen far	Mor til barnets far	Far til barnets far
Samisk	☐	☐	☐	☐
Urdu	☐	☐	☐	☐
Engelsk	☐	☐	☐	☐
Annet	☐	☐	☐	☐

Hvis annet, hvilket ____________________

79. Hva var brutto årsinntekt (før skatt) det siste året for deg og barnets far? *(Inkl. barnebidrag, arbeidsledighetstrygd, kontantstøtte, osv.)*

Din brutto årsinntekt	Brutto årsinntekt til barnets far
☐ Ingen inntekt	☐ Ingen inntekt
☐ Under 150.000 kr.	☐ Under 150.000 kr.
☐ 150-199.999 kr.	☐ 150-199.999 kr.
☐ 200-299.999 kr.	☐ 200-299.999 kr.
☐ 300-399.999 kr.	☐ 300-399.999 kr.
☐ 400-499.999 kr.	☐ 400-499.999 kr.
☐ over 500.000 kr.	☐ over 500.000 kr.
	☐ Vet ikke

80. Kan din husholdning klare seg økonomisk uten at du har inntekt?

☐ Nei
☐ Ja, men med problemer
☐ Ja, uten problemer

81. Hvilken type bolig bor du i?

☐ Enebolig
☐ Gårdsbruk
☐ Tomannsbolig
☐ Firemannsbolig
☐ Rekkehus
☐ Terrasseleilighet
☐ Kjellerleilighet/hageleilighet
☐ Blokk
☐ Bygård/leiegård. Hvilken etasje? ☐☐ etg.
☐ Annet____________________

82. Har det vært fuktskader, synlig sopp-/muggvekst eller mugglukt i boligen din i løpet av de siste 3 månedene? *(Sett eventuelt flere kryss.)*

☐ Nei
☐ Ja, fuktskader
☐ Ja, synlig sopp- /muggvekst
☐ Ja, mugglukt

83. Hva slags drikkevann er det der du bor?

☐ Vann fra offentlig eller privat vannverk
☐ Vann fra egen vannforsyning *(f.eks. egen brønn)*

84. Hvor mange ganger har du flyttet i løpet av de siste 3 årene?

☐☐ ganger

85. Har noen du bor sammen med hatt influensa, langvarig hoste, barnesykdom eller feber med utslett etter at du ble gravid?

☐ Nei
☐ Ja

86. Hvis ja, kryss av for hvilken sykdom. *(Sett eventuelt flere kryss.)*

☐ Røde hunder
☐ Vannkopper
☐ Meslinger
☐ 4. Barnesykdom
☐ Annen feber med utslett
☐ Influensa
☐ Langvarig hoste
☐ Tuberkulose
☐ Munn-hånd- og fot sykdom
☐ Annet

Levevaner

87. Røykte din mor da hun var gravid med deg?
- ☐ Nei
- ☐ Ja
- ☐ Vet ikke

88. Er du utsatt for passiv røyking hjemme?
- ☐ Nei
- ☐ Ja

89. Hvis ja, hvor mange timer pr. dag?

☐☐ timer pr. dag

90. Er du utsatt for passiv røyking på arbeid?
- ☐ Nei
- ☐ Ja

91. Hvis ja, hvor mange timer pr. dag?

☐☐ timer pr. dag

92. Røykte barnets far før du ble gravid?
- ☐ Nei
- ☐ Ja

93. Røyker han nå?
- ☐ Nei
- ☐ Ja

94. Har du noen gang røykt?
- ☐ Nei
- ☐ Ja

95. Røyker du nå (etter at du ble gravid)?
- ☐ Nei
- ☐ Av og til ☐☐ Sigaretter pr. uke
- ☐ Daglig ☐☐ Sigaretter pr. dag

96. Røykte du de siste 3 månedene før du ble gravid denne gangen?
- ☐ Nei
- ☐ Av og til ☐☐ Sigaretter pr. uke
- ☐ Daglig ☐☐ Sigaretter pr. dag

97. Hvor gammel var du da du begynte å røyke daglig?

☐☐ år

98. Har du helt sluttet å røyke?
- ☐ Nei
- ☐ Ja

99. Hvis ja, hvor gammel var du da du sluttet?

☐☐ år

100. Dersom du har sluttet å røyke etter at du ble gravid, i hvilken svangerskapsuke sluttet du å røyke?

☐☐ svangerskapsuke

101. Hvor lang tid går det fra du står opp om morgenen til du røyker din første sigarett?
- ☐ 5 minutter
- ☐ 6-29 minutter
- ☐ 30-60 minutter
- ☐ Mer enn en time

102. Røyker du når du er syk?
- ☐ Nei
- ☐ Ja

103. Røyker du oftere de første timene etter at du har våknet enn du gjør resten av dagen?
- ☐ Nei
- ☐ Ja

104. Hvis du har brukt andre former for nikotin, kryss av for hvilken type og når du har brukt den.

	Før svangerskapet	I svangerskapet
Skrå/tyggetobakk/snus	☐	☐
Nikotintyggegummi	☐	☐
Nikotinplaster	☐	☐
Nikotininhalator	☐	☐

105. Oppgi drikkemengde (antall kopper/glass) hver dag, både før du ble gravid og nå *(1 krus = 2 kopper, 1 liten plastflaske (0,5l) = 4 kopper, 1 stor plastflaske (1,5l) = 12 kopper)*

	Antall kopper/glass		Koffein-fritt *(kryss av)*
	Før svangerskapet	Nå	
1 Filterkaffe	☐☐	☐☐	☐
2 Pulverkaffe	☐☐	☐☐	☐
3 Kokekaffe	☐☐	☐☐	☐
4 Te	☐☐	☐☐	☐
5 Urtete	☐☐	☐☐	☐
6 Coca Cola, Pepsi e.l.	☐☐	☐☐	☐
7 Annen brus	☐☐	☐☐	☐
8 Coca Cola-/Pepsi-light	☐☐	☐☐	☐
9 Annen light-brus	☐☐	☐☐	☐
10 Springvann	☐☐	☐☐	
11 Flaskevann *(Farris, Olden)*	☐☐	☐☐	

	Før svangerskapet	Nå	Økologisk *(kryss av)*
12 Saft/juice	☐☐	☐☐	☐
13 Saft/juice (light)	☐☐	☐☐	☐
14 Søt skummet, lett- helmelk	☐☐	☐☐	☐
15 Cultura, alle typer	☐☐	☐☐	☐
16 Biola, alle typer	☐☐	☐☐	☐
17 Annen surmelk *(kefir)*	☐☐	☐☐	☐
18 Annet	☐☐	☐☐	☐

106. Har du noen gang brukt noen av de følgende stoffene?

	Aldri	Tidligere	I siste måned før svangerskapet	I svangerskapet
Hasj	☐	☐	☐	☐
Amfetamin	☐	☐	☐	☐
Ecstasy	☐	☐	☐	☐
Kokain	☐	☐	☐	☐
Heroin	☐	☐	☐	☐

107. Har du noen gang drukket alkohol?

☐ Nei *(gå til spørsmål 117.)*
☐ Ja

Enheter alkohol
For å sammenligne ulike typer alkohol spør vi etter det vi kaller alkohol-enheter (= 1,5 cl ren alkohol). En alkoholenhet tilsvarer:

1 flaske rusbrus / cider
1 glass (1/3 liter) øl
1 vinglass rød eller hvitvin
1 hetvinsglass, sherry eller annen hetvin
1 drammeglass brennevin eller likør

108. Hvor ofte drakk du alkohol de siste 3 månedene før svangerskapet og hvor ofte drikker du i svangerskapet?

(Sett ett kryss for hver periode)	Siste 3 måneder før svangerskapet	I svangerskapet
Omtrent 6-7 ganger pr uke	☐	☐
Omtrent 4-5 ganger pr uke	☐	☐
Omtrent 2-3 ganger pr uke	☐	☐
Omtrent 1 gang pr uke	☐	☐
Omtrent 1-3 ganger pr måned	☐	☐
Sjeldnere enn 1 gang pr måned	☐	☐
Aldri	☐	☐

109. Hvilken type alkohol drikker du vanligvis? *(Sett eventuelt flere kryss.)*

Lettøl ☐
Øl ☐
Rødvin ☐
Hvitvin ☐
Rusbrus ☐
Hetvin *(sherry, portvin, madeira)* ☐
Brennevin *(vodka, gin, akevitt, cognac, whisky, likør)* ☐

110. Har du drukket 5 alkoholenheter eller mer ved minst en anledning de siste 3 måneder før svangerskapet eller i svangerskapet?

	Siste 3 måneder før svangerskapet	I svangerskapet
Flere ganger i uken	☐	☐
1 gang i uken	☐	☐
1-3 ganger pr måned	☐	☐
Mindre enn 1 gang pr. måned	☐	☐
Aldri	☐	☐

111. Hvor mange enheter drikker du vanligvis når du nyter alkohol?

(Sett ett kryss for hver periode)	Siste 3 måneder før svangerskapet	I svangerskapet
10 eller flere	☐	☐
7-9	☐	☐
5-6	☐	☐
3-4	☐	☐
1-2	☐	☐
Færre enn 1	☐	☐

112. Hvor mange alkoholenheter kan du drikke før du merker det?

☐☐ enheter

113. Har andre irritert eller såret deg ved å kritisere hvor mye du drikker?

☐ Nei
☐ Ja

114. Har du noen ganger følt at du burde redusere alkohol forbruken din?

☐ Nei
☐ Ja

115. Har du noen ganger drukket alkohol om morgenen for å roe nervene eller bli kvitt «dagen-derpå-hodepine»?

☐ Nei
☐ Ja

116. Har du opplevd følgende problemer i det siste året i forbindelse med egen bruk av alkohol.

	Aldri	En gang	Flere ganger
Kranglet eller fått negative følelser overfor en i familien	☐	☐	☐
Plutselig befunnet deg på et sted og ikke husket hvordan du kom dit	☐	☐	☐
Vært borte fra arbeid eller skole	☐	☐	☐
Besvimt eller sluknet helt plutselig	☐	☐	☐
Hatt en trist periode	☐	☐	☐

Vekt og vektkontroll

117. Synes du selv at du var for tykk i tiden like før du ble gravid denne gangen?

☐ Ja, en god del
☐ Ja, litt
☐ Nei

118. Er du engstelig for å legge på deg mer enn nødvendig under dette svangerskapet?

☐ Ja, veldig engstelig
☐ Nokså engstelig
☐ Nei, ikke særlig engstelig

119. Har det hendt i løpet av de siste to årene at andre be-merket at du var for tynn, mens du selv syntes du var for tykk?

☐ Ja, ofte
☐ Ja, noen få ganger
☐ Nei

120. Har det hendt at du følte at du mistet kontrollen mens du spiste og ikke klarte å stoppe før du hadde spist altfor mye?

	Siste 6 måneder før svangerskapet	Nå
Nei	☐	☐
Sjelden	☐	☐
Ja, minst en gang i uken	☐	☐

121. Har du brukt noen av de følgende måtene for å kontrollere vekten?

	Siste 6 måneder før svangerskapet		Nå	
	Minst 1 gang i uken	Sjelden/ aldri	Minst 1 gang i uken	Sjelden/ aldri
Oppkast	☐	☐	☐	☐
Avføringsmidler	☐	☐	☐	☐
Fastekurer	☐	☐	☐	☐
Hard fysisk trening	☐	☐	☐	☐

122. Er det viktig for synet du har på deg selv, at du holder en bestemt vekt?

☐ Ja, svært viktig
☐ Ja, nokså viktig
☐ Nei, ikke særlig viktig

Vannlating

123. Har du lekkasje av urin i forbindelse med hosting, nysing, latter eller tunge løft?

☐ Ja
☐ Nei

124. Har du lekkasje av urin i forbindelse med plutselig og sterk vannlatingstrang?

☐ Ja
☐ Nei

125. Hvor ofte har du urinlekkasje?

☐ Aldri
☐ Sjeldnere enn en gang pr. måned.
☐ En eller flere ganger pr. måned
☐ En eller flere ganger pr. uke
☐ Hver dag og/eller hver natt

126. Hvor mye urin lekker du vanligvis hver gang?

☐ Lekker aldri
☐ Dråper eller lite
☐ Små skvetter
☐ Større mengder

127. Har du hatt urinlekkasje før dette svangerskapet?

☐ Ja
☐ Nei

128. Hvis ja;
Hadde du urinlekkasje de siste månedene før dette svangerskapet?

☐ Ja
☐ Nei

129. Oppsto urinlekkasjen første gang under et tidligere svangerskap?

☐ Ja
☐ Nei

Fysisk aktivitet

130. Hvor ofte er du fysisk aktiv? *(Kryss av for hver linje, både før og i dette svangerskap.)*

	Siste 3 måneder før dette svangerskapet					I dette svangerskapet				
	Aldri	1-3 ganger pr. måned	1 gang pr.uke	2 ganger pr.uke	3 ganger eller mer pr. uke	Aldri	1-3 ganger pr. måned	1 gang pr. uke	2 ganger pr. uke	3 ganger eller mer pr. uke
1 Rolig gange/spasertur	☐	☐	☐	☐	☐	☐	☐	☐	☐	☐
2 Rask gange/turgang	☐	☐	☐	☐	☐	☐	☐	☐	☐	☐
3 Løping/jogging/orientering	☐	☐	☐	☐	☐	☐	☐	☐	☐	☐
4 Sykling	☐	☐	☐	☐	☐	☐	☐	☐	☐	☐
5 Helsestudio/styrketrening	☐	☐	☐	☐	☐	☐	☐	☐	☐	☐
6 Spesiell gymnastikk/aerobics for gravide	☐	☐	☐	☐	☐	☐	☐	☐	☐	☐
7 Aerobics/gymnastikk/dans uten løp og hopp	☐	☐	☐	☐	☐	☐	☐	☐	☐	☐
8 Aeobics/gymnastikk/dans med løp og hopp	☐	☐	☐	☐	☐	☐	☐	☐	☐	☐
9 Dansing (swing, rock, folkedans)	☐	☐	☐	☐	☐	☐	☐	☐	☐	☐
10 Skigåing	☐	☐	☐	☐	☐	☐	☐	☐	☐	☐
11 Ballspill/nettballspill	☐	☐	☐	☐	☐	☐	☐	☐	☐	☐
12 Svømming	☐	☐	☐	☐	☐	☐	☐	☐	☐	☐
13 Riding	☐	☐	☐	☐	☐	☐	☐	☐	☐	☐
14 Annet	☐	☐	☐	☐	☐	☐	☐	☐	☐	☐

131. Hvor ofte gjør du øvelser for disse muskelgruppene? *(Kryss av for hver linje både før og i dette svangerskap.)*

	Siste 3 måneder før dette svangerskapet					I dette svangerskapet				
	Aldri	1-3 ganger pr. måned	1 gang pr.uke	2 ganger pr.uke	3 ganger eller mer pr. uke	Aldri	1-3 ganger pr. måned	1 gang pr. uke	2 ganger pr. uke	3 ganger eller mer pr. uke
Magemuskler	☐	☐	☐	☐	☐	☐	☐	☐	☐	☐
Ryggmuskler	☐	☐	☐	☐	☐	☐	☐	☐	☐	☐
Bekkenbunnsmuskler *(muskler rundt skjede, urinrør, endetarm)*	☐	☐	☐	☐	☐	☐	☐	☐	☐	☐

132. Hvor ofte er du så fysisk aktiv (i fritid eller på arbeid) at du blir andpusten eller svett?

	Siste 3 måneder før dette svangerskapet		I dette svangerskapet	
	I fritiden	På arbeid	I fritiden	På arbeid
Aldri	☐	☐	☐	☐
Mindre enn en gang pr. uke	☐	☐	☐	☐
1 gang pr. uke	☐	☐	☐	☐
2 ganger pr. uke	☐	☐	☐	☐
3-4 ganger pr. uke	☐	☐	☐	☐
5 ganger pr. uke eller mer	☐	☐	☐	☐

Litt mer om deg selv og hvordan du har det nå

133. Kryss av om du er enig eller uenig i de følgende påstandene. *(Sett kun ett kryss for hver linje.)*

	Helt uenig	Uenig	Litt uenig	Verken eller	Litt enig	Enig	Helt enig
På de fleste måter er livet mitt nær idealet mitt	☐	☐	☐	☐	☐	☐	☐
Livsbetingelsene mine er svært gode	☐	☐	☐	☐	☐	☐	☐
Jeg er fornøyd med livet mitt	☐	☐	☐	☐	☐	☐	☐
Så langt har jeg oppnådd det som er viktig for meg i livet	☐	☐	☐	☐	☐	☐	☐
Hadde jeg kunnet leve livet på nytt, ville jeg nesten ikke forandret noe	☐	☐	☐	☐	☐	☐	☐

134. Hvor enig er du i disse beskrivelsene av ditt parforhold? *(Besvares bare dersom du er i et parforhold.)*
(Sett kun ett kryss for hver linje.)

	Svært enig	Enig	Litt enig	Litt uenig	Uenig	Svært uenig
Det er et nært samhold mellom meg og min ektefelle/samboer/partner	☐	☐	☐	☐	☐	☐
Min partner og jeg har problemer i parforholdet	☐	☐	☐	☐	☐	☐
Jeg er svært lykkelig i mitt parforhold	☐	☐	☐	☐	☐	☐
Min partner er generelt forståelsesfull	☐	☐	☐	☐	☐	☐
Jeg tenker ofte på å avslutte vårt parforhold	☐	☐	☐	☐	☐	☐
Jeg er fornøyd med forholdet til min partner	☐	☐	☐	☐	☐	☐
Vi er ofte uenige om viktige avgjørelser	☐	☐	☐	☐	☐	☐
Jeg har vært heldig med valg av partner	☐	☐	☐	☐	☐	☐
Vi er enige om hvordan barn bør oppdras	☐	☐	☐	☐	☐	☐
Jeg tror min partner er fornøyd med forholdet	☐	☐	☐	☐	☐	☐

135. Har du noen utenom din ektefelle/samboer/partner som du kan søke råd hos i en vanskelig situasjon?

- ☐ Nei
- ☐ Ja 1-2 personer
- ☐ Ja flere enn to personer

136. Hvor ofte treffer du eller snakker i telefonen med familie (utenom husholdningen) eller nære venner?

- ☐ 1 gang i måneden eller sjeldnere
- ☐ 2-8 ganger i måneden
- ☐ Mer enn 2 ganger i uken

137. Føler du deg ofte ensom?

- ☐ Nesten aldri
- ☐ Sjelden
- ☐ Av og til
- ☐ Som regel
- ☐ Nesten alltid

138. Har du i løpet av de to siste ukene vært plaget med noe av det følgende? *(Kryss av for hver linje.)*

	Ikke plaget	Litt plaget	Ganske mye plaget	Veldig mye plaget
Stadig redd eller engstelig	☐	☐	☐	☐
Nervøsitet, indre uro	☐	☐	☐	☐
Følelse av håpløshet med hensyn til fremtiden	☐	☐	☐	☐
Nedtrykt, tungsindig	☐	☐	☐	☐
Mye bekymret eller urolig	☐	☐	☐	☐

139. Har du noen gang som voksen opplevd at noen har gitt deg en ørefik, slått eller sparket deg eller plaget deg fysisk på annen måte? *(Sett eventuelt flere kryss.)*

	I dette svangerskap	Siste 6 mnd før svangerskap	Tidligere
Nei	☐	☐	☐
Ja	☐	☐	☐
Husker ikke	☐	☐	☐

140. Har du noen gang opplevd at du ble presset eller tvunget til seksuell omgang?*(Sett eventuelt flere kryss.)*

	I dette svangerskap	Siste 6 mnd før svangerskap	Tidligere
Nei, aldri	☐	☐	☐
Ja, presset	☐	☐	☐
Ja, utøvd makt	☐	☐	☐
Ja, voldtatt	☐	☐	☐

141. Hva slags oppfatning har du av deg selv?*(Kryss av for hver linje.)*

	Svært enig	Enig	Uenig	Svært uenig
Jeg har en positiv holdning til meg selv	☐	☐	☐	☐
Jeg føler meg virkelig ubrukelig til tider	☐	☐	☐	☐
Jeg føler at jeg ikke har mye å være stolt av	☐	☐	☐	☐
Jeg føler at jeg er en verdifull person i alle fall på lik linje med andre	☐	☐	☐	☐

142. Har du noen ganger tidligere i livet i en sammenhengende periode på 2 uker eller mer: *(Kryss av for hver linje.)*

	Nei	Ja
Følt deg deprimert, trist, nedfor	☐	☐
Hatt problemer med matlysten eller spist for mye	☐	☐
Vært plaget av kraftløshet eller mangel på overskudd	☐	☐
Virkelig bebreidet deg selv og følt deg verdiløs	☐	☐
Hatt problemer med å konsentrere deg eller hatt vanskeligheter for å ta beslutninger	☐	☐
Hatt minst 3 av de problemene som er nevnt ovenfor samtidig	☐	☐

143. Hvis du har hatt 3 eller flere av disse problemene samtidig, hvor mange uker varte den lengste perioden?

☐☐ uker

144. Var det en spesiell grunn til dette?

- ☐ Nei, ingen spesiell grunn
- ☐ Ja *(f.eks. dødsfall, skilsmisse, abort, ulykke)*

Kommentarer

Har du husket å fylle ut dato for utfylling av skjema på side 1?

Tusen takk for hjelpen!

Legg det utfylte skjemaet i den frankerte returkonvolutten

Bording AS
