# Supplementary material for: Does progestin-only contraceptive use after pregnancy affect recovery from pelvic girdle pain? A prospective population study
Source: PLoS One. 2017 Sep 11;12(9):e0184071. doi: 10.1371/journal.pone.0184071 (PMC5593199; doi:10.1371/journal.pone.0184071)
Supplement: S5 Questionnaire — (PDF) [file pone.0184071.s005.pdf]

den norske Mor & barn undersøkelsen

Spørreskjema 3 – ca. svangerskapsuke 30

Dette spørreskjemaet gjelder for det meste tiden etter svangerskapsuke 12. Vi stiller deg en del spørsmål som du kanskje vil kjenne igjen fra det første spørreskjemaet. Vi gjør dette fordi vi ønsker å følge din og barnets utvikling videre. Det vil være en fordel om du finner frem ditt Helsekort for gravide før du begynner å besvare spørsmålene, slik at du kan benytte opplysningene som står der under utfyllingen av spørreskjemaet. Hvis du synes at et spørsmål er for ubehagelige eller vanskelig å svare på kan du la være å svare på det spørsmålet og gå videre til det neste.

Skjemaet skal leses av en maskin. Det er derfor viktig at du legger vekt på følgende ved utfyllingen:

- Bruk blå eller sort kulepenn.
- I de små avkrysningsboksene setter du *et kryss* for det svaret som du mener passer best, slik: ☒
- Hvis du mener at du har satt kryss i feil boks, kan du rette det ved å fylle boksen helt, slik: ■
- I de store, grønne boksene skriver du *tall* eller *store blokkbokstaver.*

Det er viktig at du bare skriver i det hvite feltet i boksene, slik:

Tall: 1 2 3 4 5 6 7 8 9 0   Bokstaver: A B C D

- Tallboksene har to eller flere ruter. Når du skriver et ett-sifret tall bruker du den høyre ruten. *Eksempel: 5 skrives slik* [ ][5]
- Flere steder i skjemaet ber vi om at du angir svaret i forhold til antall svangerskapsuker. *Eksempel: Hvis du skal angi noe som skjedde 14 uker etter siste menstruasjon, krysser du av for uke 13-16.*
- Spesielle opplysninger som *f.eks. medikamenter* skriver du fritt inne i boksene eller på de åpne linjene. *Vennligst skriv tydelig med STORE BOKSTAVER.*
- Husk å fylle ut dato for utfylling av skjemaet

Så snart du har fylt ut skjemaet, sender du det tilbake til oss i den vedlagte frankerte svarkonvolutten.

Oppgi dag, måned og år for utfylling av skjemaet [ ][ ] dag [ ][ ] måned [ ][ ][ ][ ] år *(skriv årstall med 4 tall, f.eks. 2001)*

Svangerskapskontroll og helse

1. Hvor har du gått til svangerskapskontroll? *(Du kan sette flere kryss).* **Oppgi hvor mange ganger på hvert sted.**

- ☐ Helsestasjon [ ][ ] ganger
- ☐ Legekontor/legesenter [ ][ ] ganger
- ☐ Sykehusets poliklinikk [ ][ ] ganger

2. Hvem har du gått til svangerskapskontroll hos? *(Du kan sette flere kryss).* **Oppgi hvor mange ganger.**

- ☐ Jordmor [ ][ ] ganger
- ☐ Allmennpraktiserende lege [ ][ ] ganger
- ☐ Gynekolog [ ][ ] ganger
- ☐ Helsesøster [ ][ ] ganger

3. Er legen du har gått til mann eller kvinne? Hvor mange ganger har du gått til ham/henne?

Allmennpraktiserende lege	☐ Kvinne	[ ][ ]	ganger
	☐ Mann	[ ][ ]	ganger
Gynekolog	☐ Kvinne	[ ][ ]	ganger
	☐ Mann	[ ][ ]	ganger

4. Hvis du går eller har gått til svangerskapskontroll hos privatpraktiserende gynekolog eller på poliklinikk, hva er eller var grunnen?

- ☐ Henvist på grunn av komplikasjoner i dette svangerskapet
- ☐ Henvist på grunn av tidligere sykdom eller komplikasjoner i tidligere svangerskap
- ☐ På eget initiativ uten henvisning
- ☐ Henvist av annen grunn

Spørreskjema 3E M&B. 20.000. 1006

5. Hvor enig er du i følgende påstander om svangerskaps-kontrollene du har gått til?

	Svært enig	Enig	Litt enig	Litt uenig	Uenig	Svært uenig
Jeg har fått tilstrekkelig råd og informasjon	☐	☐	☐	☐	☐	☐
Jeg har blitt godt ivaretatt som person	☐	☐	☐	☐	☐	☐
Jeg har ikke fått nok tid under kontrollene	☐	☐	☐	☐	☐	☐
Jeg har følt meg trygg under kontrollene	☐	☐	☐	☐	☐	☐
Jeg kunne snakke om det jeg har hatt behov for	☐	☐	☐	☐	☐	☐
Totalt sett er jeg fornøyd med helsevesenets oppfølging av meg	☐	☐	☐	☐	☐	☐

6. Har du kontaktet jordmor eller lege utenom de vanlige kontrollene?

	Nei	Ja
Jordmor	☐	☐
Lege	☐	☐

7. Hvis ja, var det vanskelig å komme til?

	Jordmor	Lege
Ikke vanskelig	☐	☐
Litt vanskelig	☐	☐
Svært vanskelig	☐	☐

8. Er det blitt gjort gynekologisk undersøkelse (innvendig undersøkelse av underlivet) av deg mens du har vært gravid? Hvis ja, hvor mange ganger?

☐ Nei

☐ Ja ☐☐ ganger

9. Hvor mange ganger i svangerskapet har du fått utført ultralydundersøkelse?

Utenpå magen ☐☐ ganger

I skjeden ☐☐ ganger

10. Hvor mange barn venter du? ☐

11. Har du fått tilbud om fostervanns- eller morkakeprøve?

☐ Nei (gå til spørsmål 16)

☐ Ja

12. Hvis ja, ble prøven(e) utført, og hva viste den/de.

	Ble prøven(e) utført? Ja	Nei	Viste prøven(e) normale forhold? Ja	Nei
Fostervannsprøve	☐	☐	☐	☐
Morkakeprøve	☐	☐	☐	☐

Dersom prøven(e) ikke viste normale forhold beskriv:

____

13. Hvis det ble utført fostervannsprøve/morkakeprøve, hva var grunnen?

☐ På grunn av min alder (vanligvis 38 år eller eldre ved termin)

☐ Tidligere barn med kromosomsykdom

☐ Tidligere barn med nevralrørsdefekt (ryggmargsbrokk)

☐ Epilepsi (medisiner mot epilepsi)

☐ Funn ved ultralyd

☐ Annet

14. Oppsto det komplikasjoner de nærmeste 2 ukene etter forstervannsprøven ble tatt?

☐ Nei

☐ Ja

15. Hvis ja, hvilke komplikasjoner?

☐ Blødning fra skjeden

☐ Fostervannslekkasje

☐ Magesmerter (menstruasjonsliknende eller sterkere)

☐ Annet ____

16. Er det blitt tatt røntgenbilder av deg under svangerskapet?

☐ Nei

☐ Ja

17. Hvis ja, hva ble det tatt bilde av? Oppgi hvor mange ganger og i hvilke svangerskapsuker *(Du kan sette flere kryss.)*

	I svangerskapsuker 0-12	13-16	17-20	21-24	25-28	29+	Antall ganger
Tenner.................	☐	☐	☐	☐	☐	☐	☐
Lunger..................	☐	☐	☐	☐	☐	☐	☐
Armer og bein........	☐	☐	☐	☐	☐	☐	☐
Bekken/mage/rygg	☐	☐	☐	☐	☐	☐	☐
Annet	☐	☐	☐	☐	☐	☐	☐

18. Har du fått behandling for å stoppe for tidlig (prematur) fødsel i dette svangerskapet? *(Du kan sette flere kryss.)*

☐ Nei

☐ Ja, måtte ta det med ro eller ligge

☐ Ja, fikk medisiner

Hvilke medisiner?____

19. Har du blitt vaksinert i dette svangerskapet?

☐ Nei

☐ Ja

Hvilken vaksine?____

20. Har jordmor eller lege sagt på svangeskapskontroll at du har/har hatt høyt blodtrykk i dette svangerskapet?

☐ Nei

☐ Ja

21. Hvis ja, hvor høyt var det på det høyeste i dette svangerskapet? *(Med høyt blodtrykk menes ett eller begge tall over 140/90) (Se i Helsekortet ditt.)*

☐☐☐ / ☐☐☐ *Eks.* 150/ 95

☐ Vet ikke

22. Har du hatt høyt blodtrykk uten å være gravid?

☐ Nei

☐ Ja

☐ Vet ikke

23. Hvis ja, hvor høyt var det på det høyeste utenom svangerskapet?

☐☐☐ / ☐☐☐ *Eks.* 150/ 95

☐ Vet ikke

24. Hva har dine blodprosent/hemoglobinverdier (Hb) vært i dette svangerskapet? *(Se i Helsekortet ditt. Noter verdi ved siste kontroll, samt høyeste og laveste verdi.)*

	Hemoglobin (Hb)	Svanger-skapsuke
Ved siste kontroll i svangerskapet	☐☐,☐	☐☐
Høyeste verdi i svangerskapet	☐☐,☐	☐☐
Laveste verdi i svangerskapet	☐☐,☐	☐☐

☐ Vet ikke

25. Hvor mye veide du ved siste kontroll, og når var det? *(Se i Helsekortet ditt.)*

Vekt ☐☐☐,☐ kg

Dato for siste kontroll ☐☐ dag ☐☐ måned ☐☐☐☐ år

26. Har du vært innlagt på sykehus etter at du ble gravid?

☐ Nei
☐ Ja, hvilke(t)sykehus____

27. Hvis ja, hva var årsaken til innleggelsen og når var det? *(Du kan sette flere kryss.)*

I hvilke svangerskapsuker var du innlagt?

	0–4	5–8	9–12	13–16	17–20	21–24	25–28	29+
☐ Langvarig kvalme/ brekninger	☐	☐	☐	☐	☐	☐	☐	☐
☐ Blødning	☐	☐	☐	☐	☐	☐	☐	☐
☐ Fostervannslekkasje	☐	☐	☐	☐	☐	☐	☐	☐
☐ Truende fødsel	☐	☐	☐	☐	☐	☐	☐	☐
☐ Høyt blodtrykk	☐	☐	☐	☐	☐	☐	☐	☐
☐ Svangerskapsforgiftning	☐	☐	☐	☐	☐	☐	☐	☐
☐ Annet ________________	☐	☐	☐	☐	☐	☐	☐	☐

28. Har du lekkasje av urin i forbindelse med hosting, nysing, latter eller tunge løft?

☐ Ja
☐ Nei

29. Har du lekkasje av urin i forbindelse med plutselig og sterk vannlatingstrang?

☐ Ja
☐ Nei

30. Hvor ofte har du urinlekkasje?

☐ Aldri
☐ Sjeldnere enn en gang pr. måned.
☐ En eller flere ganger pr. måned
☐ En eller flere ganger pr. uke
☐ Hver dag og/eller hver natt

31. Hvor mye urin lekker du vanligvis hver gang?

☐ Lekker aldri
☐ Dråper eller lite
☐ Små skvetter
☐ Større mengder

32. Har du eller har du noen gang hatt smerter i noen av de følgende kroppsdeler? Sett kryss for hvor og når det var. *(Du kan sette flere kryss.)*

		I dette svangerskap		I tidligere svangerskap		Mellom svangeskap		Før ditt første svangerskap	
	Nei	Endel plaget	Sterkt plaget	Endel plaget	Sterkt plaget	Endel plaget	Sterkt plaget	Endel plaget	Sterkt plaget
I korsryggen	☐	☐	☐	☐	☐	☐	☐	☐	☐
Over det ene bekkenleddet bak	☐	☐	☐	☐	☐	☐	☐	☐	☐
Over begge bekkenleddene bak	☐	☐	☐	☐	☐	☐	☐	☐	☐
Over halebeinet	☐	☐	☐	☐	☐	☐	☐	☐	☐
I setebalIene	☐	☐	☐	☐	☐	☐	☐	☐	☐
Foran, over kjønnsbeinet	☐	☐	☐	☐	☐	☐	☐	☐	☐
I lysken	☐	☐	☐	☐	☐	☐	☐	☐	☐
Andre ryggsmerter	☐	☐	☐	☐	☐	☐	☐	☐	☐

33. Våkner du om natten på grunn av bekkensmerter?

- ☐ Ja, ofte
- ☐ Ja, en sjelden gang
- ☐ Nei, aldri

34. Har du så store vansker med å gå på grunn av bekkensmerter at du må bruke stokk eller krykker?

- ☐ Nei, aldri
- ☐ Ja, men ikke hver dag, smertene varierer fra dag til dag
- ☐ Ja, må bruke stokk eller krykker hver dag

35. Har du fått bedøvelse i forbindelsen med operasjon eller tannlegebehandling i løpet av dette svangerskapet?

- ☐ Nei
- ☐ Ja

36. Hvis ja, hvilken type bedøvelse fikk du? *(Du kan sette flere kryss.)*

- ☐ Generell (full) narkose
- ☐ Spinal bedøvelse (i ryggmargen)
- ☐ Lokal bedøvelse
- ☐ Vet ikke

37. Har du vært hos tannlege i løpet av dette svangerskapet?

- ☐ Nei
- ☐ Ja

38. Hvis ja, har tannlegen utført noen av følgende behandlinger i dette svangerskapet? *(Du kan sette flere kryss.)*

	Ja	Nei
Satt inn nye amalgamfyllinger (sølvfyllinger) .	☐	☐
Fjernet/skiftet ut amalgamfyllinger	☐	☐
Satt inn nye hvite fyllinger	☐	☐

39. Hvor mange tenner har du totalt, og omtrent hvor mange av tennene har fyllinger? *(Se i speilet og tell dem.)*

Totalt antall tenner: ☐☐

Antall tenner med amalgamfyllinger: ☐☐

Antall tenner med andre typer fyllinger: ☐☐

40. Blør du for tiden fra tannkjøttet når du pusser tennene?

- ☐ Nei, sjelden eller aldri
- ☐ Ja, av og til
- ☐ Ja, ofte
- ☐ Ja, nesten alltid

41. Har du fått utført tatovering eller piercing inkludert ekstra hull i ørene? *(Ta ikke med vanlig hull i ørene, dvs. ett hull i hvert øre)*

- ☐ Nei
- ☐ Ja

42. Hvis ja, hvor ble det utført og når var det? *(Du kan sette flere kryss)*

	Tatovering	Piercing
Før dette svangerskapet:		
I Norge	☐	☐
I utlandet	☐	☐
I dette svangerskapet:		
I Norge	☐	☐
I utlandet	☐	☐

43. Har du noen gang fått blodoverføring? Hvis ja oppgi antall ganger.

- ☐ Nei
- ☐ Ja, i dette svangerskapet ☐ ganger
- ☐ Ja, før dette svangerskapet ☐ ganger

44. Hvis ja, i hvilke land og hvilket år?
(Oppgi de to siste gangene)

	**År**
Land: ______________________	☐☐☐☐
Land: ______________________	☐☐☐☐

45. Har du noen gang vært operert i brystene?

- ☐ Nei
- ☐ Ja

46. Hvis ja, var det:

- ☐ Brystforstørrelse
- ☐ Brystreduksjon
- ☐ Kreft/kreftprøve
- ☐ Annet beskriv: ______________________

47. Har du hatt celleforandringer på livmorhalsen?

- ☐ Nei
- ☐ Ja Årstall da det ble påvist første gang ☐☐☐☐

48. Har du blitt operert på livmorhalsen?

- ☐ Nei
- ☐ Ja Årstall da du ble operert ☐☐☐☐

49. Har du noen gang fått sprøyte med gammaglobulin?
(Brukes for å forebygge gulsott (hepatitt A) oftest i forbindelse med utenlandsreiser.)

- ☐ Nei
- ☐ Ja

Hvis ja, i hvilket år? ☐☐☐☐

Hvordan har du hatt det siden sist?

Nå følger noen spørsmål som handler om tiden etter 13. svangerskapsuke.

50. Har du hatt en eller flere blødninger fra skjeden etter 13. svangerskapsuke?

- ☐ Nei
- ☐ Ja

51. Hvis ja, kryss av for hvor mye du blødde, i hvilke svangerskapsuker og hvor mange dager blødningen varte. *(Hvis mer enn 2 blødninger, beskriv de 2 siste.)*

	Kryss av for blødningsmengde *(sporblødning betyr noen dråper)*			I hvilke svangerskapsuker hadde du blødningen? 13–16	17–20	21–24	25–28	29+	Blødningen varte i antall dager
1.	☐ Sporblødning	☐ Mer enn sporblødning	☐ Større mengder	☐	☐	☐	☐	☐	☐☐
2.	☐ Sporblødning	☐ Mer enn sporblødning	☐ Større mengder	☐	☐	☐	☐	☐	☐☐

☐ Hvis mer enn 2 blødningsepisoder, angi antall ☐☐

52. Kjenner du årsaken til at du blødde?

- ☐ Nei
- ☐ Ja

53. Hvis ja, hva var årsaken(e)? *(Du kan sette flere kryss.)*

- ☐ Morkaken ligger for langt nede (vanskelig plassert/ placenta prævia)
- ☐ For tidlig løsning av morkaken (abruptio/ablatio placenta)
- ☐ Truende abort/for tidlig fødsel
- ☐ Sår på livmorhalsen, slimhinneblødning
- ☐ Etter samleie
- ☐ Annen årsak

54. Har du vært plaget av kynnere / forveer?

- ☐ Nei
- ☐ Ja, litt plaget
- ☐ Ja, mye plaget

55. Har du eller har du hatt noen av de følgende sykdommene eller helseplagene etter 13. svangerskapsuke? Hvis du har brukt tabletter, miksturer, stikkpiller, inhalasjoner, salver osv. i forbindelse med sykdommen eller helseplage, oppgi navnet på medisinen(e) og når du brukte dem og hvor lenge. *(Du kan sette flere kryss.) (Dette gjelder alle typer medikamenter, både faste og ikke-faste og naturmedisiner. Ikke før inn vitaminer og kost-tilskudd - disse spør vi om senere i skjemaet.)*

	I hvilke svangerskapsuker var du plaget? 13-16	17-20	21-24	25-28	29+	Navn på medisiner du brukte	I hvilke svangerskapsuker brukte du medisiner? 13-16	17-20	21-24	25-28	29+	Antall dager brukt
1 Bekkenløsning	☐	☐	☐	☐	☐	________	☐	☐	☐	☐	☐	☐☐☐
2 Ryggsmerter	☐	☐	☐	☐	☐	________	☐	☐	☐	☐	☐	☐☐☐
3 Andre smerter i muskler/ledd	☐	☐	☐	☐	☐	________	☐	☐	☐	☐	☐	☐☐☐
4 Kvalme	☐	☐	☐	☐	☐	________	☐	☐	☐	☐	☐	☐☐☐
5 Langvarig kvalme med brekninger/oppkast .	☐	☐	☐	☐	☐	________	☐	☐	☐	☐	☐	☐☐☐
6 Soppinfeksjon i skjeden	☐	☐	☐	☐	☐	________	☐	☐	☐	☐	☐	☐☐☐
7 Annen skjedekatarr/ uvanlig utflod	☐	☐	☐	☐	☐	________	☐	☐	☐	☐	☐	☐☐☐
8 Svangerskapskløe . .	☐	☐	☐	☐	☐	________	☐	☐	☐	☐	☐	☐☐☐
9 Treg mage	☐	☐	☐	☐	☐	________	☐	☐	☐	☐	☐	☐☐☐
10 Diaré/omgangssyke .	☐	☐	☐	☐	☐	________	☐	☐	☐	☐	☐	☐☐☐

Fortsettelse...

	I hvilke svangerskapsuker var du plaget? 13-16	17-20	21-24	25-28	29+	Navn på medisiner du brukte	I hvilke svangerskapsuker brukte du medisiner? 13-16	17-20	21-24	25-28	29+	Antall dager brukt
11 Uvanlig tretthet/-søvnighet . .	☐	☐	☐	☐	☐	______	☐	☐	☐	☐	☐	
12 Halsbrann/sure oppstøt	☐	☐	☐	☐	☐	______	☐	☐	☐	☐	☐	
13 Hevelse i kroppen (ødem)	☐	☐	☐	☐	☐	______	☐	☐	☐	☐	☐	
14 Forkjølelse	☐	☐	☐	☐	☐	______	☐	☐	☐	☐	☐	
15 Halsbetennelse	☐	☐	☐	☐	☐	______	☐	☐	☐	☐	☐	
16 Bihule/-ørebetennelse	☐	☐	☐	☐	☐	______	☐	☐	☐	☐	☐	
17 Influensa	☐	☐	☐	☐	☐	______	☐	☐	☐	☐	☐	
18 Lungebetennelse/ bronkitt	☐	☐	☐	☐	☐	______	☐	☐	☐	☐	☐	
19 Annen hoste	☐	☐	☐	☐	☐	______	☐	☐	☐	☐	☐	
20 Sukker i urinen	☐	☐	☐	☐	☐	______	☐	☐	☐	☐	☐	
21 Eggehvite (protein) i urin	☐	☐	☐	☐	☐	______	☐	☐	☐	☐	☐	
22 Blærekatarr	☐	☐	☐	☐	☐	______	☐	☐	☐	☐	☐	
23 Urinlekkasje	☐	☐	☐	☐	☐	______	☐	☐	☐	☐	☐	
24 Høyt blodtrykk	☐	☐	☐	☐	☐	______	☐	☐	☐	☐	☐	
25 Leggkramper	☐	☐	☐	☐	☐	______	☐	☐	☐	☐	☐	
26 Astma	☐	☐	☐	☐	☐	______	☐	☐	☐	☐	☐	
27 Høysnue/annen allergi	☐	☐	☐	☐	☐	______	☐	☐	☐	☐	☐	
28 Hodepine/migrene . .	☐	☐	☐	☐	☐	______	☐	☐	☐	☐	☐	
29 Depresjon	☐	☐	☐	☐	☐	______	☐	☐	☐	☐	☐	
30 Andre psykiske plager	☐	☐	☐	☐	☐	______	☐	☐	☐	☐	☐	
31 Annet	☐	☐	☐	☐	☐	______	☐	☐	☐	☐	☐	

56. Dersom du har hatt feber en eller flere ganger etter 13. svangerskapsuke, angi i hvilke svangerskapsuker, navn på febernedsettende medisiner du har brukt og høyeste målte temperatur. *(Hvis mer enn 3 ganger, kryss av for de 3 siste.)*

	I hvilke svangerskapsuker hadde du feber? 13–16	17–20	21–24	25–28	29+	Hvis du brukte febernedsettende medisiner, hvilke medisiner brukte du?	Høyeste målte temperatur *(f.eks. 38,9° C)*	Måle ikke temperatur
1. gang	☐	☐	☐	☐	☐	______	__ __ , __ °C	☐
2. gang	☐	☐	☐	☐	☐	______	__ __ , __ °C	☐
3. gang	☐	☐	☐	☐	☐	______	__ __ , __ °C	☐

☐ Feber mer enn 3 ganger

57. Har du brukt andre medisiner etter 13. svangerskapsuke som du ikke har nevnt tidligere, for eksempel sovemidler eller beroligende midler? Oppgi navn, når du har tatt disse og hvor mange dager tilsammen. *(Ta med alle typer medisiner, både faste og ikke-faste og naturmedisiner, men ikke vitaminer og kosttilskudd – det kommer i neste spørsmål.)*

**Navn på medisiner** *(f.eks. Valium, Rohypnol, Paracet)*	Bruk av medisiner i svangerskapsuker 13–16	17–20	21–24	25–28	29+	Antall dager brukt
	☐	☐	☐	☐	☐	
	☐	☐	☐	☐	☐	
	☐	☐	☐	☐	☐	
	☐	☐	☐	☐	☐	

58. Har du i løpet av dette svangerskapet vært utsatt for en ulykke eller skade *(f.eks. trafikkulykke, fall, slag mot magen)?*

☐ Nei
☐ Ja

59. Hvis ja, i hvilken svangerskapsuke?

Vitaminer, mineraler og kosttilskudd

60. Har du brukt vitaminer, mineraler eller annet kosttilskudd etter 13. svangeskapsuke?

☐ Nei *(gå til spørsmål 61)*
☐ Ja

61. Hvis ja, vennligst finn frem eske/glass og bruk innholdslisten til å fylle ut tabellen nedenfor: *(F.eks. hvis du har tatt tran hver dag fra svangerskapsuke 13, skal du sette ett kryss for hver periode under "I hvilke svangerskapsuke" (dvs. 5 kryss) og ett kryss for "Daglig" under "Hvor ofte".)*

	I hvilke svangerskapsuker brukte du tilskudd? 13–16	17–20	21–24	25–28	29+	Omtrent hvor ofte brukte du dette? Daglig	4-6 ganger pr. uke	1-3 ganger pr. uke
1 Folat/folsyre	☐	☐	☐	☐	☐	☐	☐	☐
2 Vitamin B1 (Thiamin)	☐	☐	☐	☐	☐	☐	☐	☐
3 Vitamin B2 (Riboflavin)	☐	☐	☐	☐	☐	☐	☐	☐
4 Vitamin B6 (Pyrioksin)	☐	☐	☐	☐	☐	☐	☐	☐
5 Vitamin B12 (Cyanokobalamin)	☐	☐	☐	☐	☐	☐	☐	☐
6 Niacin	☐	☐	☐	☐	☐	☐	☐	☐
7 Pantotensyre (pantotenat)	☐	☐	☐	☐	☐	☐	☐	☐
8 Biotin	☐	☐	☐	☐	☐	☐	☐	☐
9 Vitamin C	☐	☐	☐	☐	☐	☐	☐	☐
10 Vitamin A	☐	☐	☐	☐	☐	☐	☐	☐
11 Vitamin D	☐	☐	☐	☐	☐	☐	☐	☐
12 Vitamin E	☐	☐	☐	☐	☐	☐	☐	☐
13 Jern	☐	☐	☐	☐	☐	☐	☐	☐
14 Kalk/kalsium	☐	☐	☐	☐	☐	☐	☐	☐
15 Jod	☐	☐	☐	☐	☐	☐	☐	☐
16 Sink	☐	☐	☐	☐	☐	☐	☐	☐
17 Selen	☐	☐	☐	☐	☐	☐	☐	☐
18 Kobber	☐	☐	☐	☐	☐	☐	☐	☐
19 Krom	☐	☐	☐	☐	☐	☐	☐	☐
20 Magnesium	☐	☐	☐	☐	☐	☐	☐	☐
21 Tran	☐	☐	☐	☐	☐	☐	☐	☐
22 Omega-3 fettsyre	☐	☐	☐	☐	☐	☐	☐	☐

62. Oppgi fullstendig produktnavn på alle vitaminer og kosttilskudd du bruker. Ta også med naturmedisiner og slankepulver.
(Skriv tydelig med blokkbokstaver da det skal leses maskinelt.)

1 F.eks. V I T A P L E X M E D J E R N
2 ☐
3 ☐
4 ☐
5 ☐
6 ☐

63. Hvis du bruker multivitaminer (med eller uten mineraler), inneholder disse folat/folsyre?

☐ Nei
☐ Ja
☐ Vet ikke

Arbeid

64. Har du hatt inntektsgivende arbeid i dette svangerskapet?

☐ Nei *(gå til spørsmål 76)*
☐ Ja

65. Er du i samme arbeidssituasjon nå etter 13. svangerskapsuke som det du beskrev i det første spørreskjemaet?

☐ Nei
☐ Ja *(Gå til spørsmål 66)*

66. Hvis nei, i hvilken svangerskapsuke ble arbeidssituasjonen endret?

Svangerskapsuke ☐☐

67. Hvordan er arbeidssituasjonen din endret?

☐ Jeg har sluttet i arbeid
☐ Jeg har gått ned i redusert stilling
☐ Annet

68. Hvis du har sluttet, hvorfor gjorde du det?

☐ Jeg sa opp selv
☐ Arbeidet var midlertidig (sesongarbeid, engasjement e.l.)
☐ Jeg ble sagt opp
☐ Annet

69. Er arbeidsforholdene dine blitt endret i løpet av svangerskapet slik at de passer bedre for deg som gravid?

☐ Nei
☐ Ja

70. Hvis nei, hvorfor er ikke arbeidsforholdene blitt endret slik at de passer bedre for deg?

☐ Det har vært unødvendig
☐ Det er umulig eller nesten umulig
☐ Jeg har bedt om forandringer, men ikke fått det
☐ Det er vanskelig å spørre
☐ Ingen av svarene passer *(forklar gjerne nærmere)*

---

71. Hvilken arbeidstidsordning har du nå? *(Du kan sette flere kryss.)*

☐ Fast dagarbeid
☐ Fast ettermiddags- eller kveldsarbeid
☐ Fast nattarbeid
☐ Skiftarbeid eller turnusordning
☐ Ingen fast ordning (ekstrahjelp, ekstravakt, vikar og lignende)
☐ Annen ordning

72. Kryss av for følgende spørsmål som gjelder nåværende arbeid. *(Kryss av for hver linje.)*

	Ja, daglig, mer enn halve arbeidstiden	Ja, daglig, mindre enn halve arbeidstiden	Ja, i perioder, men ikke daglig	Sjelden eller aldri
Hender det at du har så mye å gjøre at arbeidssituasjonen blir oppjaget og masete? . .	☐	☐	☐	☐
Må du vri eller bøye deg mange ganger i timen?	☐	☐	☐	☐
Arbeider du med hendene løftet i skulderhøyde eller høyere?	☐	☐	☐	☐
Arbeider du stående/gående?	☐	☐	☐	☐
I noen jobber kan man selv bestemme når ulike arbeidsoppgaver skal gjøres og i hvilke tempo. Man kan for eksempel velge å arbeide litt raskere visse dager og litt roligere andre dager. Har du den muligheten?	☐	☐	☐	☐
Er du utsatt for så mye støy eller lyder at du synes det er ubehagelig?	☐	☐	☐	☐
Er du utsatt for så mye støy eller lyder at du må heve stemmen for å snakke med andre, selv på en meters avstand?	☐	☐	☐	☐

73. Hvor ofte har du arbeidet ved radiosender eller radar etter 13. svangerskapsuke?

- ☐ Sjelden/aldri
- ☐ Noen ganger i uken
- ☐ Daglig
- ☐ I gjennomsnitt mer enn 1 time daglig

74. Hvor ofte har du arbeidet ved røntgenapparat (mindre enn 2 meters avstand) etter 13. svangerskapsuke? *(Ta ikke med behandling som pasient.)*

- ☐ Sjelden/aldri
- ☐ Noen ganger i uken
- ☐ Daglig
- ☐ I gjennomsnitt mer enn 1 time daglig

75. Har du vært fraværende fra ditt vanlige arbeid i mer enn to uker etter 13. svangerskapsuke?

- ☐ Nei
- ☐ Ja, delvis
- ☐ Ja

76. Er du fraværende fra ditt vanlige arbeid nå for tiden?

- ☐ Nei
- ☐ Ja, delvis
- ☐ Ja

77. Hvis ja, hva er årsaken til fraværet nå for tiden? *(Sett kun ett kryss.)*

- ☐ Sykemelding (fravær med sykepenger)
- ☐ Fravær på grunn av sykt barn
- ☐ Permittering med dagpenger
- ☐ Fravær med svangerskapspenger pga. arbeidsmiljø
- ☐ Begynte fødselspermisjon (fravær med fødselspenger)
- ☐ Tjenestepermisjon
- ☐ Annet, beskriv:

____

____

____

78. Hvis du har vært sykemeldt etter 13. svangerskapsuke, skriv opp i tabellen årsaken til sykmelding evt. delvis sykemelding (f.eks. bekkenløsning, lungebetennelse). Kryss av i hvilke svangerskapsuker du var sykemeldt og hvor mange dager hver sykemedlingen varte. Oppgi også hvor mange prosent du var sykemeldt hver gang.
(Skriv en sykemelding per linje, se eksempel):

Årsak til sykemelding:	Var sykemeldt i svangerskapsuker 13-16	17-20	21-24	25-28	29+	Antall dager per sykemelding	% sykemeldt
Eksempel: bekkenløsning	☐	☒	☐	☐	☐	1 4	5 0
	☐	☐	☐	☐	☐		
	☐	☐	☐	☐	☐		
	☐	☐	☐	☐	☐		
	☐	☐	☐	☐	☐		

79. Løfter du nå når du er gravid noe som veier mer enn 10 kilo?
(10 kg tilsvarer vekten av en vannbøtte)

	Hjemme	Arbeid
Sjelden eller aldri	☐	☐
Ja, mindre enn 20 ganger ukentlig	☐	☐
Ja, mer enn 20 ganger ukentlig	☐	☐
Ja, 10 til 20 ganger daglig	☐	☐
Ja, mer enn 20 ganger daglig	☐	☐

80. Har andre tatt på seg mer enn vanlig av husarbeid eller omsorg for barn for å avlaste deg i dette svangerskapet?

- ☐ Ja, i stor grad
- ☐ Ja, i noen grad
- ☐ Nei, ingen har tilbudt seg
- ☐ Nei, det har ikke vært nødvendig med slik avlastning

81. Hvis du har begynt i permisjon (fødselspermisjon for dette svangerskapet), når begynte du?

Dato: ☐☐ dag ☐☐ måned ☐☐☐☐ år

Levevaner

82. Hvor ofte snakker du i mobiltelefon?

- ☐ Sjelden/aldri
- ☐ Noen ganger i uken
- ☐ Daglig
- ☐ I gjennomsnitt mer enn 1 time daglig

83. Varer en mobiltelefonsamtale i mer enn 15 minutter?

- ☐ Aldri
- ☐ Sjelden
- ☐ Ofte

84. Hvor ofte har du arbeidet ved dataskjerm, laserprinter eller kopieringsmaskin (mindre enn 2 meters avstand) etter 13. svangerskapsuke?

	Data-skjerm	Laser-printer	Kopierings-maskin
Sjelden/aldri	☐	☐	☐
Noen ganger i uken	☐	☐	☐
Daglig	☐	☐	☐
I gjennomsnitt mer enn 1 time daglig	☐	☐	☐

85. Bor du i nærheten av en høyspentledning?

- ☐ Nei
- ☐ Ja, nærmere enn 50 meter
- ☐ Ja, 50 – 100 meter
- ☐ Ja, men mer enn 100 meter fra.

86. Hvor ofte har du gått på diskotek siden du fylte ut forrige spørreskjema?

- ☐ Aldri
- ☐ Minst 1–2 ganger i uken
- ☐ Sjeldnere

87. Hvor ofte er du fysisk aktiv nå for tiden? *(Kryss av for hver linje.)*

	Aldri	1-3 ganger pr. måned	1 gang pr. uke	2 ganger pr. uke	3 ganger eller mer pr. uke
1 Rolig gange/spasertur	☐	☐	☐	☐	☐
2 Rask gange/turgang	☐	☐	☐	☐	☐
3 Løping/jogging/orientering	☐	☐	☐	☐	☐
4 Sykling	☐	☐	☐	☐	☐
5 Helsestudio/styrketrening	☐	☐	☐	☐	☐
6 Spesiell gymnastikk/aerobics for gravide	☐	☐	☐	☐	☐
7 Aerobics/gymnastikk/dans uten løp og hopp	☐	☐	☐	☐	☐
8 Aerobics/gymnastikk/dans med løp og hopp	☐	☐	☐	☐	☐
9 Dansing (swing, rock, folkedans)	☐	☐	☐	☐	☐
10 Skigåing	☐	☐	☐	☐	☐
11 Ballspill/nettballspill	☐	☐	☐	☐	☐
12 Svømming	☐	☐	☐	☐	☐
13 Riding	☐	☐	☐	☐	☐
14 Annet	☐	☐	☐	☐	☐

88. Hvor ofte gjør du øvelser for disse muskelgruppene hjemme eller på trening nå for tiden? *(Kryss av for hver linje.)*

	Aldri	1-3 ganger pr. måned	1 gang pr. uke	2 ganger pr. uke	3 ganger eller mer pr. uke
Magemuskler	☐	☐	☐	☐	☐
Ryggmuskler	☐	☐	☐	☐	☐
Bekkenbunnsmuskler *(muskler rundt skjede, urinrør, endetarm)*	☐	☐	☐	☐	☐

89. Hvor ofte er du så fysisk aktiv (i fritid eller på arbeid) nå for tiden at du blir anpusten eller svett?

	I fritiden	På arbeid
Aldri	☐	☐
Mindre enn en gang pr. uke	☐	☐
1 gang pr uke	☐	☐
2 ganger pr. uke	☐	☐
3-4 ganger pr. uke	☐	☐
5 ganger pr. uke eller mer	☐	☐

90. Hvor ofte har du hatt samleie gjennomsnittlig i løpet av den siste måneden?

- ☐ Daglig
- ☐ 5–6 ganger i uken
- ☐ 3–4 ganger i uken
- ☐ 1–2 ganger i uken
- ☐ Sjeldnere
- ☐ Ingen ganger

91. Har du vært utenlands i løpet av det siste året?

- ☐ Nei
- ☐ Ja

92. Hvis ja, i hvilke land har du vært og når?

Land	Måned	År
______	☐☐	☐☐☐☐
______	☐☐	☐☐☐☐
______	☐☐	☐☐☐☐

93. Har du i ditt arbeid eller din fritid kontakt med dyr?

- ☐ Nei
- ☐ Ja

94. Hvis ja, hva slags dyr og hvor ofte er du i kontakt med dyr?

	Daglig	3–6 ganger pr. uke	1-2 ganger pr. uke	Sjeldnere
Hund	☐	☐	☐	☐
Katt	☐	☐	☐	☐
Marsvin, hamster, kanin, rotte o.l	☐	☐	☐	☐
Undulat og annen fugl inne	☐	☐	☐	☐
Høner og annet fjærkre	☐	☐	☐	☐
Ku, sau, geit	☐	☐	☐	☐
Hest	☐	☐	☐	☐
Gris	☐	☐	☐	☐
Annet	☐	☐	☐	☐

95. Hvor mange timer sover du vanligvis i døgnet nå når du er gravid?

☐ Over 10 timer
☐ 8–9 timer
☐ 6–7 timer
☐ 4–5 timer
☐ Mindre enn 4 timer

96. Pleier du nå for tiden å sove i vannseng eller bruke elektrisk varmeteppe?

	Ja	Nei
Vannseng	☐	☐
Varmeteppe	☐	☐

97. Har du mulighet for å hvile deg litt på dagen (gjelder både hjemme og på arbeid)?

☐ Nei
☐ Ja

98. Har du vært i sauna (badstue) mens du har vært gravid?

☐ Nei
☐ 1–5 ganger
☐ 6–10 ganger
☐ Mer enn 10 ganger

99. Har du vært i solarium mens du har vært gravid?

☐ Nei
☐ 1–5 ganger
☐ 6 – 10 ganger
☐ Mer enn 10 ganger

100. Er du utsatt for passiv røyking hjemme eller på arbeid? Hvis ja, hvor mange timer pr. dag?

	Nei	Ja	Antall timer
Hjemme	☐	☐	☐☐
Arbeid	☐	☐	☐☐

101. Røyker du nå for tiden? Hvis ja, hvor mange sigaretter?

☐ Nei
☐ Av og til ☐☐ Sigaretter pr. uke
☐ Daglig ☐☐ Sigaretter pr. dag

102. Røyker barnefaren nå for tiden? Hvis ja, hvor mange sigaretter?

☐ Nei
☐ Av og til ☐☐ Sigaretter pr. uke
☐ Daglig ☐☐ Sigaretter pr. dag

103. Dersom en av dere eller begge har sluttet å røyke i svangerskapet, i hvilken svangerskapsuke var dette?

☐ Deg selv ☐☐ Svangerskapsuke
☐ Barnefaren ☐☐ Svangerskapsuke

104. Hvis du eller barnefaren har røykt i svangerskapet, har det vært perioder da du eller barnefaren ikke har røykt? *(Sett kryss som viser i hvilke svangerskapsuker dere ikke har røykt.)*

	Svangerskapsuker da dere *ikke* røykte							
	0–4	5–8	9–12	13–16	17–20	21–24	25–28	29+
Deg selv....	☐	☐	☐	☐	☐	☐	☐	☐
Barnefaren	☐	☐	☐	☐	☐	☐	☐	☐

105. Har du brukt andre former for nikotin etter 13. svangerskapsuke?

	Nei	Ja
Nikotintyggegummi	☐	☐
Nikotinplaster	☐	☐
Nikotininnhalator	☐	☐
Skrå/tyggetobakk/snus	☐	☐

106. Har du brukt noen av følgende rusmidler etter 13. svangerskapsuke?

	Nei	Ja
Hasj	☐	☐
Amfetamin	☐	☐
Ecstasy	☐	☐
Kokain	☐	☐
Heroin	☐	☐

107. Har du noen gang brukt noen av de følgende stoffene? *(Kryss av for hver linje.)*

	Aldri	Tidligere	Det siste halvåret før du ble gravid	I dette svangerskapet
Anabole steroider	☐	☐	☐	☐
Testosteronpreparater	☐	☐	☐	☐
Veksthormon *(eks. Genotropin/Somatropin)*	☐	☐	☐	☐

Mat og drikke

108. Hvor ofte spiser du følgende matvarer? *(Kryss av for hver linje.)*

	Før svangerskapet				I svangerskapet		
	Aldri	Noen få ganger i året	1–3 ganger i måneden	En gang i uken eller oftere	Aldri	1–3 ganger i måneden	En gang i uken eller oftere
1 Krabbe	☐	☐	☐	☐	☐	☐	☐
2 Reker	☐	☐	☐	☐	☐	☐	☐
3 Skjell (f.eks. blåskjell, o-skjell)	☐	☐	☐	☐	☐	☐	☐
4 Fiskelever	☐	☐	☐	☐	☐	☐	☐
5 Tunfisk eller kveite (hellefisk)	☐	☐	☐	☐	☐	☐	☐
6 Flyndre/annen flatfisk	☐	☐	☐	☐	☐	☐	☐
7 Gjedde eller abbor	☐	☐	☐	☐	☐	☐	☐
8 Annen ferskvannsfisk	☐	☐	☐	☐	☐	☐	☐
9 Reinsdyr	☐	☐	☐	☐	☐	☐	☐
10 Sauekjøtt	☐	☐	☐	☐	☐	☐	☐
11 Lever eller nyre fra vilt	☐	☐	☐	☐	☐	☐	☐
12 Viltvoksende sopp	☐	☐	☐	☐	☐	☐	☐

109. Hvor ofte spiser du følgende type mat i dette svangerskapet? *(Kryss av for hver linje.)*

	Aldri	Noen få ganger i året	1–3 ganger i måneden	En gang i uken eller oftere
Mat fra restaurant, gatekjøkken, kantine eller lignende	☐	☐	☐	☐
Kjøttvarer (unntatt hermetikk) kjøpt i utlandet	☐	☐	☐	☐
Kjøtt (inkludert fjærkre) som er rått eller ufullstendig stekt/kokt (rosa ved beinet)	☐	☐	☐	☐
Rå kjøttdeig, farse, kjøtt (eventuelt smaker på)	☐	☐	☐	☐
Røket/gravet laks eller ørret	☐	☐	☐	☐
Bløte oster (f.eks. kremost, camembert, blåost o.l.)	☐	☐	☐	☐
Uvaskete rå grønnsaker, uvasket frukt	☐	☐	☐	☐

110. Unngår du å spise følgende type mat i dette svangerskapet?

	Nei	Ja
Fisk	☐	☐
Egg	☐	☐
Nøtter	☐	☐
Appelsiner/sitroner	☐	☐
Jordbær	☐	☐

Annet, hva? ____________________

111. Hva slags drikkevann er det der du bor?

☐ Egen vannforsyning (f.eks. egen brønn)
☐ Vann fra vannverk (offentlig eller privat)
☐ Andre kilder

Navn på vannverk ____________________

☐ Vet ikke navn på vannverk

112. Er drikkevannet behandlet (klorert eller UV-bestrålt)?

☐ Nei
☐ Ja, UV-bestrålt
☐ Ja, klorert
☐ Vet ikke

113. Oppgi drikkemengde (antall kopper/glass) hver dag etter 13. svangerskapsuke.
(1 krus = 2 kopper, 1 liten plastflaske (0,5 l) = 4 kopper, 1 stor plastflaske (1,5 l) = 12 kopper)

	Ant. kopper/glass	Koffeinfritt *(kryss av)*
1. Filterkaffe	☐☐	☐
2. Pulverkaffe	☐☐	☐
3. Kokekaffe	☐☐	☐
4. Annen kaffe	☐☐	☐
5. Te	☐☐	☐
6. Coca Cola, Pepsi e.l.	☐☐	☐
7. Annen brus	☐☐	☐
8. Coca Cola-/Pepsi light	☐☐	☐
9. Annen light-brus	☐☐	☐
10. Springvann	☐☐	
11. Flaskevann	☐☐	

	Ant. kopper/ glass	Økologisk (kryss av)
12. Saft/juice		☐
13. Saft/juice light		☐
14. Søtmelk (skummet, lett, hel)		☐
15. Cultura, alle typer		☐
16. Biola, alle typer		☐
17. Annen surmelk (kefir, yoghurt e.l.)		☐
18. Annet		☐

114. Hvor ofte drakk du alkohol før, og hvor ofte drikker du nå?

	Siste 3 måneder før siste mens	I dette svangerskapet Svangerskapsuke 0–12	13–24	25+
Omtrent 6–7 ganger pr. uke	☐	☐	☐	☐
Omtrent 4–5 ganger pr.uke	☐	☐	☐	☐
Omtrent 2–3 ganger pr.uke	☐	☐	☐	☐
Omtrent 1 gang pr.uke	☐	☐	☐	☐
Omtrent 1–3 ganger pr.måned	☐	☐	☐	☐
Sjeldnere enn 1 gang pr.måned	☐	☐	☐	☐
Aldri	☐	☐	☐	☐

Enheter alkohol

For å sammenligne ulike typer alkohol, spør vi etter det vi kaller alkoholenheter (= 1,5cl ren alkohol). I praksis betyr dette følgende:

1 glass (1/3 liter) øl	= 1 enhet
1 vinglass rød eller hvit vin	= 1 enhet
1 hetvinsglass, sherry eller annen hetvin	= 1 enhet
1 drammeglass brennevin eller likør	= 1 enhet
1 flaske rusbrus/cider	= 1 enhet

115. I perioden rett før du ble gravid og i dette svangerskapet, hvor mange ganger har du drukket 5 eller flere enheter? *(Se forklaring om enheter alkohol.)*

	Siste 3 måneder før siste mens	I dette svangerskapet Svangerskapsuke 0–12	13–24	25+
Flere ganger i uken	☐	☐	☐	☐
1 gang i uken	☐	☐	☐	☐
1–3 ganger pr. måned	☐	☐	☐	☐
Under 1 gang pr. måned	☐	☐	☐	☐
Aldri	☐	☐	☐	☐

116. Hvor mange enheter drikker du vanligvis når du nyter alkohol? *(Se forklaring ovenfor om enheter alkohol.)*

	Siste 3 måneder før siste mens	I dette svangerskapet Svangerskapsuke 0–12	13–24	25+
10 eller flere	☐	☐	☐	☐
7–9	☐	☐	☐	☐
5–6	☐	☐	☐	☐
3–4	☐	☐	☐	☐
1–2	☐	☐	☐	☐
Færre enn 1	☐	☐	☐	☐

117. Dersom du har endret ditt alkoholbruk før eller under dette svangerskapet, når skjedde endringen? *(Du kan sette flere kryss.)*

	Endring til mindre mengder	Endring til større mengder
Siste 3 måneder før siste menstruasjon	☐	☐
I svangerskapsuker 0–6	☐	☐
I svangerskapsuker 7–12	☐	☐
I svangerskapsuker 13–24	☐	☐
Etter svangerskapsuke 25	☐	☐

118. Hvis du har endret ditt alkoholbruk, hvor viktig var de følgende faktorene? *(Du kan sette flere kryss.)*

	Ikke aktuelt	Lite viktig	Ganske viktig	Viktig	Svært viktig
Kvalme/ubehag	☐	☐	☐	☐	☐
Endret smak	☐	☐	☐	☐	☐
For fosterets skyld	☐	☐	☐	☐	☐
Depresjon/vansker	☐	☐	☐	☐	☐
Andre grunner	☐	☐	☐	☐	☐

Litt mer om deg selv og hvordan du har det nå

119. Hvilken sivilstand har du nå?

- ☐ Gift
- ☐ Samboer
- ☐ Enslig
- ☐ Skilt/separert
- ☐ Enke
- ☐ Annet

120. Har du noen utenom ektefelle/ samboer/ partner som du kan søke råd hos i en vanskelig situasjon?

- ☐ Nei
- ☐ Ja, 1–2 personer
- ☐ Ja, flere enn to personer

121. Hvor ofte treffer du, eller snakker i telefonen med familie (utenom husholdningen) eller nære venner?

- ☐ En gang i måneden eller sjeldnere
- ☐ 2–8 ganger i måneden
- ☐ Mer enn 2 ganger i uken

122. Føler du deg ofte ensom?

- ☐ Nesten aldri
- ☐ Sjelden
- ☐ Av og til
- ☐ Som regel
- ☐ Nesten alltid

123. Dersom du har født tidligere, hvordan var din opplevelse av selve fødselen i det store og hele?

- ☐ Veldig god
- ☐ God
- ☐ Sånn passe
- ☐ Dårlig
- ☐ Veldig dårlig

124. Hvor enig er du i disse beskrivelsene av dine tanker om forestående fødsel? *(Sett kun ett kryss for hver linje.)*

	Svært enig	Enig	Litt enig	Litt uenig	Uenig	Svært uenig
Jeg vil gjerne ha en så naturlig fødsel som mulig uten smertestillende og uten inngrep						
Jeg gruer meg veldig til fødselen						
Jeg vil gjerne ha så mye bedøvelse at fødselen blir helt smertefri						
Jeg vil helst ha epidural (ryggmargsbedøvelse) uansett						
Jeg vil helst ha epidural (ryggmargsbedøvelse) hvis jordmoren også er enig						
Hvis jeg fikk velge ville jeg helst ta keisersnitt						
Jeg mener kvinnen selv må bestemme om det skal gjøres keisersnitt						
Jeg bekymrer meg hele tiden for at barnet ikke skal være friskt og velskapt						
Jeg gleder meg til barnet kommer						

125. Hvor enig er du i disse beskrivelsene av ditt parforhold? *(Besvares bare dersom du er i et parforhold.) (Sett kun ett kryss for hver linje.)*

	Svært enig	Enig	Litt enig	Litt uenig	Uenig	Svært uenig
Det er et nært samhold mellom meg og min ektefelle/samboer/partner						
Min partner og jeg har problemer i parforholdet						
Jeg er svært lykkelig i mitt parforhold						
Min partner er generelt forståelsesfull						
Jeg tenker ofte på å avslutte vårt parforhold						
Jeg er fornøyd med forholdet til min partner						
Vi er ofte uenige om viktige avgjørelser						
Jeg har vært heldig med valg av partner						
Vi er enige om hvordan barn bør oppdras						
Jeg tror min partner er fornøyd med forholdet						

126. Har du i løpet av de 2 siste ukene vært plaget med noe av det følgende? *(Sett kun ett kryss for hver linje.)*

	Ikke plaget	Litt plaget	Ganske mye plaget	Veldig mye plaget
Stadig redd eller engstelig				
Nervøsitet, indre uro				
Følelse av håpløshet med hensyn til fremtiden				
Nedtrykt, tungsindig				
Mye bekymret eller urolig				
Følelse av at alt er et slit				
Føler deg anspent eller oppjaget				
Plutselig frykt uten grunn				

127. Hvor ofte opplever du følgende i ditt daglige liv? *(Sett kun ett kryss for hver linje.)*

	Sjelden/ aldri	Nokså sjelden	Noen ganger	Ofte	Veldig ofte
Føler deg glad for noe					
Føler deg lykkelig					
Føler deg oppstemt, som om alt legger seg til rette for deg					
Føler at du vil skrike til noen eller slå løs på ting					
Føler deg sint, irritert eller ergerlig					
Føler deg rasende på noen					

128. Hvor riktige er disse utsagnene for deg? *(Sett kun ett kryss for hver linje.)*

	Ikke riktig	Litt riktig	Nesten riktig	Helt riktig
Jeg klarer alltid å løse vanskelige problemer hvis jeg prøver hardt nok				
Hvis noen motarbeider meg, finner jeg en måte å oppnå det jeg vil på				
Jeg er sikker på at jeg kan mestre uventede hendelser				
Jeg er rolig når jeg møter vanskeligheter, fordi jeg stoler min evne til å klare meg				
Dersom jeg er i en knipe, finner jeg vanligvis en løsning				

129. Kryss av om du er enig eller uenig i de følgende påstandene. *(Sett kun ett kryss for hver linje.)*

	Helt uenig	Uenig	Litt uenig	Verken eller	Litt enig	Enig	Helt enig
På de fleste måter er livet mitt nær idealet mitt	☐	☐	☐	☐	☐	☐	☐
Livsbetingelsene mine er svært gode	☐	☐	☐	☐	☐	☐	☐
Jeg er fornøyd med livet mitt	☐	☐	☐	☐	☐	☐	☐
Så langt har jeg oppnådd det som er viktig for meg i livet	☐	☐	☐	☐	☐	☐	☐
Hadde jeg kunnet leve livet på nytt, ville jeg nesten ikke forandret noe	☐	☐	☐	☐	☐	☐	☐

130. Hva slags oppfatning har du av deg selv? *(Sett kun ett kryss for hver linje.)*

	Svært enig	Enig	Uenig	Svært uenig
Jeg har en positiv holdning til meg selv	☐	☐	☐	☐
Jeg føler meg virkelig ubrukelig til tider	☐	☐	☐	☐
Jeg føler at jeg ikke har mye å være stolt av	☐	☐	☐	☐
Jeg føler at jeg er en verdifull person, iallfall på lik linje med andre	☐	☐	☐	☐

131. Har du i løpet av de siste 12 månedene opplevd noe av det følgende? Hvis ja, hvor vondt eller vanskelig var dette for deg? *(Kryss av for hver linje.)*

	Nei	Ja	Hvis ja: Ikke så ille	Hvis ja: Vondt/ vanskelig	Hvis ja: Veldig vondt/ vanskelig
Har du hatt problemer på arbeidsplassen eller der du utdanner deg	☐	☐	☐	☐	☐
Har du hatt økonomiske problemer	☐	☐	☐	☐	☐
Ble du skilt, separert eller avbrøt du samlivet	☐	☐	☐	☐	☐
Har du hatt problemer eller konflikter med familie, venner eller naboer	☐	☐	☐	☐	☐
Har du vært alvorlig syk eller skadet	☐	☐	☐	☐	☐
Har en av dine nærmeste vært alvorlig syk eller skadet	☐	☐	☐	☐	☐
Har du vært utsatt for alvorlig trafikkulykke, boligbrann eller grovt tyveri	☐	☐	☐	☐	☐
Har du mistet en som sto deg nær	☐	☐	☐	☐	☐
Annet	☐	☐	☐	☐	☐

132. Har du noen gang opplevd noe av det følgende: *(Kryss av for hver linje.)*

	Nei, aldri	Ja, som barn *(under 18 år)*	Ja, som voksen *(over 18 år)*	Hvem eller hvilke personer var det som utsatte deg for det? Fremmed person	Familie eller slektning	Annen kjent person	Har dette skjedd deg det siste året? Nei	Ja
At noen systematisk og over lengre tid har forsøkt å kue, fornedre eller ydmyke deg?	☐	☐	☐	☐	☐	☐	☐	☐
At noen har truet med å skade deg eller noen som står deg nær?	☐	☐	☐	☐	☐	☐	☐	☐
Å bli utsatt for fysiske overgrep?	☐	☐	☐	☐	☐	☐	☐	☐
Å bli presset til seksuelle handlinger?	☐	☐	☐	☐	☐	☐	☐	☐

Annet

133. Har noen du bor sammen med hatt noen av de følgende sykdommer i løpet av dette svangerskapet? *(Kryss av og angi tidspunkt.)*

	I hvilke svangerskapsuker? 0–9	10–19	20–29	30+
☐ Influensa	☐	☐	☐	☐
☐ Barnesykdom (feber med utslett)	☐	☐	☐	☐
☐ Langvarig hoste	☐	☐	☐	☐
☐ Annen infeksjonssykdom	☐	☐	☐	☐

134. Har det vært noen tilfeller av krybbedød i din familie eller i barnefarens familie?

☐ Nei
☐ Vet ikke
☐ Ja, i min familie, *(se spørsmål 132)*
☐ Ja, i barnefarens familie, *(se spørsmål 133)*

135. Barnet som døde i krybbedød i min familie var:

☐ Min søster
☐ Min bror
☐ Min søsters barn ☐ Gutt ☐ Pike
☐ Min brors barn ☐ Gutt ☐ Pike
☐ Min mors søsken ☐ Gutt ☐ Pike ☐ Kjønn ukjent
☐ Min fars søsken ☐ Gutt ☐ Pike ☐ Kjønn ukjent
☐ Andre

136. Barnet som døde i krybbedød i barnefarens familie var:

- ☐ Barnefarens søster
- ☐ Barnefarens bror
- ☐ Barnefarens søsters barn ☐ Gutt ☐ Pike
- ☐ Barnefarens brors barn ☐ Gutt ☐ Pike
- ☐ Barnefarens mors søsken ☐ Gutt ☐ Pike ☐ Kjønn ukjent
- ☐ Barnefarens fars søsken ☐ Gutt ☐ Pike ☐ Kjønn ukjent
- ☐ Andre

137. Har du noen gang mistet et barn?

- ☐ Nei *(hvis nei, er du ferdig med spørsmålene)*
- ☐ Ja

138. Hvis ja, hva var dødsårsaken og når skjedde det?

- ☐ Dødfødsel *(Fødsel etter 16. svangerskapsuke.)*
- ☐ Krybbedød
- ☐ Ulykke
- ☐ Sykdom/misdannelse

Hvilken sykdom/misdannelse: ______________________

- ☐ Annet

	Årstall	Barnets alder hvis barnet døde etter fødselen: år	måneder
Barn 1	☐☐☐☐	☐☐	☐☐
Barn 2	☐☐☐☐	☐☐	☐☐

139. Dersom du/dere fikk støtte fra helsepersonell eller andre i tiden etter dødsfallet vil vi gjerne vite mer om dette. Hvor mange samtaler hadde dere med helsepersonell og/eller foreldreforening, familie og venner og i hvor mange uker varte oppfølgningen?

	Helse-personell	Foreldreforening, familie, venner
Antall samtaler ved møter (ca.):	☐☐	☐☐
Antall samtaler pr. telefon (ca.):	☐☐	☐☐
Uker med oppfølgning (ca.):	☐☐	☐☐

140. Synes du alt i alt at du/dere fikk god nok oppfølging etter barnets død?

- ☐ Ingen tilbud om oppfølging
- ☐ Svært god
- ☐ God nok
- ☐ Burde vært bedre
- ☐ Dårlig

141. Har dødsfallet gjort deg mer engstelig i dette svangerskapet?

- ☐ Nei, ikke i det hele tatt
- ☐ Nei, ikke vesentlig
- ☐ Ja, noe mer
- ☐ Ja, i stor grad

142. Opplever du at personalet på svangerskapskontrollen har tatt hensyn til denne vonde opplevelsen i sin kontakt med deg?

- ☐ Ja, i stor grad
- ☐ Ja, noe
- ☐ Nei, ikke i det hele tatt

Kommentarer

____

____

____

____

____

Har du husket å fylle ut dato for utfylling av skjema på side 1?

Tusen takk for innsatsen!
