# Supplementary material for: Does progestin-only contraceptive use after pregnancy affect recovery from pelvic girdle pain? A prospective population study
Source: PLoS One. 2017 Sep 11;12(9):e0184071. doi: 10.1371/journal.pone.0184071 (PMC5593199; doi:10.1371/journal.pone.0184071)
Supplement: S6 Questionnaire — (PDF) [file pone.0184071.s006.pdf]

den norske **Mor & barn undersøkelsen**

Spørreskjema 5 – Når barnet er 18 måneder

I dette spørreskjemaet stiller vi en del spørsmål som du kanskje vil kjenne igjen fra tidligere spørreskjemaer. Vi gjør dette fordi vi ønsker å følge din og barnets utvikling videre. Det er fint om du finner frem barnets Helsekort, slik at du kan benytte opplysningene som står der.

Hvis du synes at et spørsmål er for ubehagelig eller vanskelig å svare på kan du la være å svare på det spørsmålet og gå videre til det neste.

Skjemaet skal leses av en maskin. ***Det er derfor viktig at du legger vekt på følgende ved utfyllingen:***

- Bruk blå eller sort kulepenn.
- I de små avkrysningsboksene setter du *et kryss* for det svaret som du mener passer best, slik: ☒
- Hvis du mener at du har satt kryss i feil boks, kan du rette det ved å fylle boksen helt, slik: ■
- I de store, grønne boksene skriver du *tall.*

Det er viktig at du bare skriver i det hvite feltet i boksene, slik:

Tall: 1 2 3 4 5 6 7 8 9 0

- Tallboksene har to eller flere ruter. Når du skriver et ett-sifret tall bruker du den høyre ruten. *Eksempel: 5 skrives slik* ☐ 5
- Spesielle opplysninger som *f.eks. medikamenter* skriver du fritt på de åpne linjene.
  Vennligst skriv tydelig med STORE BOKSTAVER.
- Husk å fylle ut dato for utfylling av skjemaet

Så snart du har fylt ut dette skjemaet, ber vi om at du sender det tilbake til oss i den vedlagte frankerte svarkonvolutten.

Oppgi dag, måned og år for utfylling av skjemaet ☐☐ dag ☐☐ måned ☐☐☐☐ år *(skriv årstall med 4 tall, f.eks. 2001)*

OM BARNET

Mat og drikke

1. Hvilken type melk har barnet fått siden hun/han var 6 måneder gammel?
(Du kan sette flere kryss.)

	Barnets alder i måneder			
**Melketype**	6 - 8	9 - 11	12 - 14	15 - 18
1. Brystmelk	☐	☐	☐	☐
2. Morsmelkerstatning	☐	☐	☐	☐
3. Melkeerstatning ved melkeallergi/intoleranse	☐	☐	☐	☐
4. Helmelk (søt)	☐	☐	☐	☐
5. Lettmelk vanlig (søt)	☐	☐	☐	☐
6. Ekstra lett melk (søt)	☐	☐	☐	☐
7. Skummet melk (søt)	☐	☐	☐	☐
8. Biola – alle typer	☐	☐	☐	☐
9. Annen yoghurt	☐	☐	☐	☐
10. Andre surmelk typer	☐	☐	☐	☐

Spørreskjema 5 M&B. 7.000. 0902

2. Hvor ofte får barnet følgende drikke nå ved ca. 18 måneders alder? (*Kryss av for hver linje.*)

	Aldri/ sjelden	1-3 ganger pr. uke	4-6 ganger pr. uke	Minst en gang daglig
1. Brystmelk	☐	☐	☐	☐
2. Morsmelkerstatning	☐	☐	☐	☐
3. H-melk	☐	☐	☐	☐
4. Lett skummet melk (søt)	☐	☐	☐	☐
5. Biola – alle typer	☐	☐	☐	☐
6. Annen yoghurt	☐	☐	☐	☐
7. Andre surmelk typer	☐	☐	☐	☐
8. Vann fra springen	☐	☐	☐	☐
9. Vann kjøpt på flaske	☐	☐	☐	☐
10. Juice	☐	☐	☐	☐
11. Saft, sukret	☐	☐	☐	☐
12. Saft, kunstig søtet (lettprodukt)	☐	☐	☐	☐
13. Brus	☐	☐	☐	☐
14. Lettbrus	☐	☐	☐	☐
15. Annet: ___________________	☐	☐	☐	☐

3. Får barnet følgende drikke om natten nå ved ca. 18 måneders alder? (*Kryss av for hver linje.*)

	Aldri/ sjelden	Av og til	Ja, de fleste netter
1. Vann	☐	☐	☐
2. Melk eller saft fra kopp	☐	☐	☐
3. Melk eller saft fra flaske	☐	☐	☐
4. Ammes	☐	☐	☐

4. Hvor ofte får barnet følgende mat nå ved ca. 18 måneders alder? (*Kryss av for hver linje.*)

	Aldri/ sjelden	1-3 ganger pr. uke	4-6 ganger pr. uke	Minst en gang daglig
1. Brødmat med leverpostei	☐	☐	☐	☐
2. Brødmat med kjøttpålegg	☐	☐	☐	☐
3. Brødmat med fisk (f.eks. sardin, makrell)	☐	☐	☐	☐
4. Brødmat med ost	☐	☐	☐	☐
5. Brødmat med syltetøy/honning	☐	☐	☐	☐
6. Brødmat med annet pålegg	☐	☐	☐	☐
7. Barnegrøt med jern	☐	☐	☐	☐
8. Annen grøt	☐	☐	☐	☐
9. Kjøtt, pølse, kjøttboller o.l.	☐	☐	☐	☐
10. Fisk, fiskeboller, fiskepudding o.l.	☐	☐	☐	☐
11. Poteter	☐	☐	☐	☐
12. Pasta	☐	☐	☐	☐
13. Ris	☐	☐	☐	☐
14. Erter, bønner	☐	☐	☐	☐
15. Andre kokte grønnsaker	☐	☐	☐	☐
16. Rå grønnsaker.	☐	☐	☐	☐
17. Frukt.	☐	☐	☐	☐
18. Yoghurt/annen surmelk med frukt/bær	☐	☐	☐	☐
19. Kake/dessert	☐	☐	☐	☐
20. Sjokolade/godterier	☐	☐	☐	☐

5. Får barnet hjemmelaget middagsmat eller ferdiglaget (indutrifremstilt) barnemat på glass?

- ☐ Bare hjemmelaget
- ☐ Mest hjemmelaget
- ☐ Omtrent halvparten av hvert
- ☐ Mest ferdiglaget

6. Får barnet økologisk dyrket mat/drikke?

- ☐ Nei
- ☐ Ja
- ☐ Vet ikke

7. Hvis barnet får økologisk mat/drikke, hva slags og hvor ofte? *(Kryss av for hver linje.)*

	Aldri/ sjelden	Av og til	Ofte	Nesten alltid
Søt melk	☐	☐	☐	☐
Sur melk/yoghurt . .	☐	☐	☐	☐
Grønnsaker/frukt . .	☐	☐	☐	☐
Grøt/mel/brød	☐	☐	☐	☐
Kjøtt	☐	☐	☐	☐

8. Reagerer barnet på visse matvarer ?

- ☐ Nei
- ☐ Ja
- ☐ Vet ikke

9. Hvis ja, hvilken type mat reagerer barnet på? *(Du kan sette flere kryss.)*

1. ☐ H-melk
2. ☐ Skummet melk/lettmelk
3. ☐ Fløte
4. ☐ Yoghurt/sur melk
5. ☐ Iskrem
6. ☐ Ost
7. ☐ Rått egg (f.eks eggedosis)
8. ☐ Egg, kokt eller stekt
9. ☐ Fisk/fiskeprodukter
10. ☐ Tilsettingsstoffer
11. ☐ Hvete
12. ☐ Nøtter
13. ☐ Soya
14. ☐ Frukt, bær
15. ☐ Grønnsaker/poteter
16. ☐ Sjokolade
17. ☐ Andre godterier
18. ☐ Sukker
19. ☐ Annet: ________________________________

10. Er det noen matvarer du bevisst unngår å gi barnet ditt?

- ☐ Nei
- ☐ Ja

11. Hvis ja, hvilke matvarer prøver du å unngå, og hvor streng er dietten?

	Noe redusert bruk i forhold til vanlig kosthold	Bruker ikke i ublandet form, men tillater litt i forskjellige matretter	Unngår all bruk (også "skjult" i matretter)
1. Melk	☐	☐	☐
2. Egg	☐	☐	☐
3. Fisk/fiskeprodukter	☐	☐	☐
4. Kjøtt/kjøttprodukter	☐	☐	☐
5. Hvete	☐	☐	☐
6. Sukker	☐	☐	☐
7. Annet: ____	☐	☐	☐

12. Får barnet tran, vitaminer, jern eller annet kosttilskudd?

- ☐ Nei
- ☐ Ja

13. Hvis ja, kryss av for hvilke(t) preparat og hvor ofte barnet får det. Hvor gammelt var barnet da dere startet med preparatet for første gang?

	Hvor ofte får barnet dette?		Hvor gammelt var barnet da dere startet med preparatet?
	Daglig	Av og til	Antall måneder
1. Tran	☐	☐	
2. Biovit	☐	☐	
3. Sanasol	☐	☐	
4. Collett spedbarnsvitaminer	☐	☐	
5. Fluortabletter	☐	☐	
6. Jerntilskudd, hvilket: ______________________________	☐	☐	
7. Annet kosttilskudd, hvilket:_________________________	☐	☐	

Vekst, helse og sykdom

Vennligst finn frem barnets helsekort og bruk opplysninger derfra til å fylle ut de følgende spørsmålene.

14. Hvor mange ganger har du/dere vært på helsestasjonen med barnet siden fødselen?

- ☐ 0 - 4
- ☐ 5 - 10
- ☐ 11 - 15
- ☐ 16 eller flere

15. Ønsker du at barnet skal få de vaksinene som anbefales til barn i Norge?

- ☐ Ja, alle vaksiner
- ☐ Ja, noen vaksiner
- ☐ Nei, ingen vaksiner

16. Kryss av for hvilke vaksiner barnet har fått? Kryss også av for hvor mange ganger barnet har fått vaksinen samt om det har vært noen bivirkning som medførte kontakt med lege eller sykehus.

	Hvor mange ganger?			Bivirkning som medførte ekstra kontakt med lege		Bivirkning som medførte undersøkelse/ innleggelse på sykehus	
**Vaksiner**	1	2	3	Nei	Ja	Nei	Ja
1. DTP (Infanrix)	☐	☐	☐	☐	☐	☐	☐
2. DT (Difteri/tetanus)	☐	☐	☐	☐	☐	☐	☐
3. Polio – Hib (Act-Hib polio)	☐	☐	☐	☐	☐	☐	☐
4. MMR (Meslinger, kusma, røde hunder)	☐	☐	☐	☐	☐	☐	☐
5. Hepatitt B (Engerix-B)	☐	☐	☐	☐	☐	☐	☐
6. BCG (tuberkulose)	☐	☐	☐	☐	☐	☐	☐
7. Annen vaksine:_________________________	☐	☐	☐	☐	☐	☐	☐

17. Hvor enig er du i følgende påstander om helsestasjonen? *(Kryss av for hver linje.)*

	Helt enig	Litt enig	Litt uenig	Helt uenig
Jeg får god informasjon på helsestasjonen.	☐	☐	☐	☐
Jeg kan snakke om det jeg har behov for.	☐	☐	☐	☐
Jeg føler meg trygg på at helsestasjonen kan vurdere mitt barns helse og utvikling.	☐	☐	☐	☐

18. Har eller har barnet hatt noen av følgende helseproblemer. Hvis ja, har helsestasjonen eller andre henvist barnet til videre spesialistundersøkelse? *(Kryss av for hver linje.)*

Helseproblem	Nei	Ja	Hvis ja, er barnet henvist til spesialistundersøkelse? Nei	Ja, fra helsestasjonen	Ja, fra andre
1. Hofter	☐	☐	☐	☐	☐
2. Hørsel	☐	☐	☐	☐	☐
3. Syn	☐	☐	☐	☐	☐
4. Forsinket motorisk utvikling (f.eks sitter/går sent)	☐	☐	☐	☐	☐
5. For liten vektøkning	☐	☐	☐	☐	☐
6. For stor vektøkning	☐	☐	☐	☐	☐
7. Avvikende hodeomkrets	☐	☐	☐	☐	☐
8. Hjertefeil	☐	☐	☐	☐	☐
9. Testiklene ikke kommet ned i pungen	☐	☐	☐	☐	☐
10. Astma	☐	☐	☐	☐	☐
11. Atopisk (barne)eksem	☐	☐	☐	☐	☐
12. Elveblest	☐	☐	☐	☐	☐
13. Matallergi/intoleranse	☐	☐	☐	☐	☐
14. Annen misdannelse: ________________________	☐	☐	☐	☐	☐
15. Annet: ________________________________	☐	☐	☐	☐	☐

19. Hvis barnet ble undersøkt videre, hva viste denne undersøkelsen?

☐ Alt var i orden
☐ Fortsatt tvil/utredes videre
☐ Har ikke vært til undersøkelse ennå
☐ Fått følgende diagnose:___________________________

20. Har barnet vært behandlet med "pute" for hoftelidelse?

☐ Nei
☐ Ja Hvor lenge? ☐☐ måneder

21. Har barnet hatt noen av følgende sykdom/helseproblem de siste 12 måneder? Kryss av om barnet har hatt problemet ved 6-11 måneders alder og/eller ved 12-18 måneders alder. Oppgi også hvor mange ganger og om barnet ble innlagt på sykehus for problemet? *(Kryss av for hver linje.)*

Sykdom/helseproblem	Ved 6 –11 måneders alder: Nei	Ja	Antall ganger	Ved 12–18 måneders alder: Nei	Ja	Antall ganger	Vært innlagt på sykehus for dette: Nei	Ja
1. Forkjølelse	☐	☐	☐☐	☐	☐	☐☐	☐	☐
2. Halsbetennelse	☐	☐	☐☐	☐	☐	☐☐	☐	☐
3. Ørebetennelse	☐	☐	☐☐	☐	☐	☐☐	☐	☐
4. Falsk krupp	☐	☐	☐☐	☐	☐	☐☐	☐	☐
5. Bronkitt/RS-virus/lungebetennelse	☐	☐	☐☐	☐	☐	☐☐	☐	☐
6. Omgangssyke/diare	☐	☐	☐☐	☐	☐	☐☐	☐	☐
7. Urinveisinfeksjon	☐	☐	☐☐	☐	☐	☐☐	☐	☐
8. Øyekatarr	☐	☐	☐☐	☐	☐	☐☐	☐	☐
9. Feberkramper	☐	☐	☐☐	☐	☐	☐☐	☐	☐
10. Andre kramper (uten feber)	☐	☐	☐☐	☐	☐	☐☐	☐	☐
11. Skade eller ulykke	☐	☐	☐☐	☐	☐	☐☐	☐	☐
12. Annet helseproblem:____________	☐	☐	☐☐	☐	☐	☐☐	☐	☐

22. Har barnet vært til lege eller på sykehus ved 6–11 måneders alder og/eller ved 12 – 18 måneders alder?
Hvis ja, oppgi hvor mange ganger. *(Kryss av for hver linje.)*

	Ved 6 – 11 måneders alder			Ved 12 – 18 måneders alder		
	Nei	Ja	Antall ganger	Nei	Ja	Antall ganger
Allmennlege (utenom helsestasjonen)	☐	☐	☐☐	☐	☐	☐☐
Legevaktlege	☐	☐	☐☐	☐	☐	☐☐
Privatpraktiserende spesialist	☐	☐	☐☐	☐	☐	☐☐
Poliklinikk på sykehus	☐	☐	☐☐	☐	☐	☐☐
Innlagt på sykehus	☐	☐	☐☐	☐	☐	☐☐

23. Hvis barnet har vært undersøkt eller innlagt på sykehus, oppgi navn på sykehus.

Navn på sykehus: ______________________________

Navn på sykehus: ______________________________

Navn på sykehus: ______________________________

24. Har barnet hatt noen av følgende symptomer de siste 12 månedene? Hvis ja, ved hvilken alder? *(Kryss av for hver linje.)*

	Hatt symptomene?		Hvis ja, ved hvilken alder?			
	Nei	Ja	6–8 mnd	9–11 mnd	12–14 mnd	15 mnd eller mer
1. Piping/hvesing i brystet	☐	☐	☐	☐	☐	☐
2. Tetthet i brystet	☐	☐	☐	☐	☐	☐
3. Nattlig hoste	☐	☐	☐	☐	☐	☐
4. Rennende nese uten forkjølelse	☐	☐	☐	☐	☐	☐
5. Forstoppelse	☐	☐	☐	☐	☐	☐
6. Diare	☐	☐	☐	☐	☐	☐
7. Kløende utslett som kommer og går	☐	☐	☐	☐	☐	☐

25. Har barnet vært allergitestet?

☐ Nei
☐ Ja

26. Hvis ja, hva ble barnet testet på og slo testen ut?
(Du kan sette flere kryss.)

Test:		Slo testen ut? Nei	Ja	Vet ikke
1.	☐ Melk	☐	☐	☐
2.	☐ Egg	☐	☐	☐
3.	☐ Fisk	☐	☐	☐
4.	☐ Muggsopp	☐	☐	☐
5.	☐ Midd	☐	☐	☐
6.	☐ Dyr	☐	☐	☐
7.	☐ Pollen	☐	☐	☐
8.	☐ Annet: ________________	☐	☐	☐

27. Har dere oppsøkt såkalt alternativ medisin for barnet de siste 12 månedene?

☐ Nei
☐ Ja ☐☐ ganger

28. Hvis ja, hva slags alternativ medisin?

____

____

____

____

____

29. Har barnet fått medisiner de siste 12 måneder?

☐ Nei
☐ Ja

30. Hvis ja, oppgi navn på medisinene og barnets alder ved medisinbruk. *(Ta med alle typer medisiner også naturmedisiner)*

Navn på medisinen (*f.eks. Apocillin, Paracet*)	Hvor gammelt var barnet ved medisinbruk			
	6-8 mnd	9-11 mnd	12-14 mnd	15–18 mnd
______________________	☐	☐	☐	☐
______________________	☐	☐	☐	☐
______________________	☐	☐	☐	☐
______________________	☐	☐	☐	☐

31. Hva var barnets lengde, vekt og hodeomkrets når barnet var omtrent 8 måneder, ca 1 år og ved siste måling (15 – 18 måneder)?

	Dato for måling					
	dag	måned	år	Vekt	Lengde	Hodeomkrets
Ca 8 mnd				*g*	*cm*	*cm*
Ca. 1 år				*g*	*cm*	*cm*
15 – 18 mnd				*g*	*cm*	

Utvikling og væremåte

32. Hvor mange måneder var barnet da det tok sitt første skritt uten støtte?

Antall: ☐☐ måneder

☐ Går ikke uten støtte ennå.

33. Nedenfor følger spørsmål om barnets utvikling ved ca. 18 måneders alder. *(Kryss av for hver linje.)*

	Ja	Av og til	Ikke ennå
1. Når barnet vil ha noe, forteller barnet det til deg ved å peke mot tingen?	☐	☐	☐
2. Dersom du ber om det, går barnet ditt inn i et annet rom og henter en kjent gjenstand? (Når du for eksempel spør: «Hvor er ballen din?», «Hent jakka di» eller «Gå og hent teppet ditt.»)	☐	☐	☐
3. Sier barnet åtte eller flere ord i tillegg til ”mamma” og ”pappa”?	☐	☐	☐
4. Peker barnet på riktig bilde når du sier for eksempel «Hvor er katten/pusen» eller «Hvor er hunden/vovven»?	☐	☐	☐
5. Beveger barnet seg omkring ved å gå heller enn å krabbe?	☐	☐	☐
6. Går barnet støtt, uten å falle ofte?	☐	☐	☐
7. Kan barnet gå ned trapper dersom du holder det i én hånd?	☐	☐	☐
8. Kan barnet kaste en liten ball med armbevegelse framover? (Dersom barnet bare slipper ballen, kryss av for ”ikke ennå”)	☐	☐	☐
9. Stabler barnet en liten kloss eller leke oppå en annen? (For eksempel små esker eller leker som er cirka 3 cm store)	☐	☐	☐
10. Blar barnet selv om sidene i en bok? (Det kan bla mer enn en side om gangen.)	☐	☐	☐
11. Gir barnet ditt dukker og kosedyr klemmer når det leker?	☐	☐	☐
12. Forsøker barnet ditt å fange oppmerksomheten din eller vise deg noe ved å dra deg i hånden eller i klærne?	☐	☐	☐
13. Kommer barnet til deg når det trenger hjelp, for eksempel for å få hjelp til å trekke opp en leke?	☐	☐	☐
14. Tar barnet ditt etter handlinger du gjør, for eksempel tørker opp søl, feier, barberer seg eller grer håret?	☐	☐	☐

34. Hvor typisk er følgende atferd for ditt barn? *(Kryss av for hver linje.)*

	Svært typisk	Ganske typisk	Både og	Lite typisk	Ikke typisk
1. Det skal lite til før barnet gråter	☐	☐	☐	☐	☐
2. Barnet er alltid på farten	☐	☐	☐	☐	☐
3. Barnet vil heller leke med andre enn å leke for seg selv	☐	☐	☐	☐	☐
4. Barnet er i aktivitet og løper omkring med en gang det våkner om morgenen	☐	☐	☐	☐	☐
5. Barnet er svært sosialt	☐	☐	☐	☐	☐
6. Det tar lang tid før barnet blir vant til fremmede	☐	☐	☐	☐	☐
7. Det skal lite til før barnet hisser seg opp eller blir lei seg	☐	☐	☐	☐	☐
8. Barnet foretrekker rolige stillesittende leker framfor mer aktive	☐	☐	☐	☐	☐
9. Barnet liker å være sammen med andre mennesker	☐	☐	☐	☐	☐
10. Barnet reagerer intenst når det blir opphisset	☐	☐	☐	☐	☐
11. Barnet er vennlig og tillitsfull mot fremmede	☐	☐	☐	☐	☐

35. Om hvordan barnet leker. *(Kryss av for hver linje.)*

	Ja	Nei
1. Liker barnet å bli svinget rundt, husket på kneet, eller andre slike leker?	☐	☐
2. Hender det at barnet kommer bort til deg med leker eller andre ting for å vise deg noe?	☐	☐
3. Liker barnet å klatre på ting, for eksempel oppover trapper?	☐	☐
4. Liker barnet å leke “borte-titt-titt” / gjemsel?	☐	☐
5. Leker barnet noen ganger “late-som-“-leker, for eksempel skjenker kaffe på liksom?	☐	☐
6. Hender det at barnet bruker pekefingeren for å vise at han/hun er interessert i noe?	☐	☐
7. Leker barnet med småleker, for eksempel lekebiler eller klosser (virkelig leker med dem, ikke bare putter dem i munnen, fingrer med dem eller slipper dem ned?	☐	☐
8. Er barnet interessert i andre barn?	☐	☐

36. Mer om barnets væremåte. Hvordan passer følgende utsagn? *(Kryss av for hver linje.)*

	Passer ikke	Passer litt	Passer godt
1. Barnet kan ikke være opptatt av noe mer enn noen minutter av gangen	☐	☐	☐
2. Barnet er lett å avlede	☐	☐	☐
3. Barnet mister fort interessen for noe	☐	☐	☐
4. Barnet klynger seg til voksne/er for avhengig	☐	☐	☐
5. Barnet blir ute av seg når mor/far ikke er der	☐	☐	☐
6. Barnet vil ikke sove alene	☐	☐	☐
7. Barnet slåss med andre barn	☐	☐	☐
8. Barnet sparker, slår, eller biter andre barn	☐	☐	☐
9. Barnet plager andre barn	☐	☐	☐
10. Barnet trøster hvis andre gråter og er lei seg	☐	☐	☐
11. Barnet prøver å hjelpe barn som har slått seg	☐	☐	☐
12. Barnet er trassig	☐	☐	☐
13. Det merkes ikke på barnet hvis hun/han har gjort noe som ikke er lov	☐	☐	☐
14. Irettesettelse har ikke effekt på barnets atferd	☐	☐	☐
15. Barnet har vanligvis god matlyst	☐	☐	☐
16. Barnet er svært kresen i matveien	☐	☐	☐
17. Barnet er lett å legge, og sovner vanligvis greit	☐	☐	☐
18. Barnet vil ikke sovne hvis ikke mor/far er der til det sovner	☐	☐	☐

37. Hvor ofte hender det at barnet våkner om natten, nå ved 18 måneders alder?

- ☐ 3 eller flere ganger hver natt
- ☐ 1-2 ganger hver natt
- ☐ Noen ganger i uken
- ☐ Sjelden eller aldri

38. Hvor mange timer sover barnet til sammen pr. døgn, nå ved 18 måneders alder??

- ☐ 10 timer eller mindre
- ☐ 11 – 12 timer
- ☐ 13 – 14 timer
- ☐ 15 timer eller mer

39. Hvis det er noe med barnets væremåte eller oppdragelse som bekymrer deg, hvem finner du det mest naturlig å snakke med? *(Du kan sette flere kryss.)*

- ☐ Ektefelle/partner
- ☐ Venner
- ☐ Slektninger
- ☐ Naboer/arbeidskolleger
- ☐ Barnehage/dagmamma
- ☐ Helsesøster
- ☐ Leger
- ☐ Andre

Barnets hverdag

40. Hvor har barnet vært passet på dagtid? Kryss av for ulike alderstrinn. *(Kryss av for hver linje.)*

	Hjemme med mor/far	Hjemme med dag-mamma/praktikant	Hos dagmamma/familiebarnehage	Barnehage
1. 0–6 måneder	☐	☐	☐	☐
2. 7–9 måneder	☐	☐	☐	☐
3. 10–12 måneder	☐	☐	☐	☐
4. 13–15 måneder	☐	☐	☐	☐
5. 16–18 måneder	☐	☐	☐	☐

41. Hvor mange timer pr. uke er barnet i nåværende barnepass-ordning (annet enn pass av mor og far)?

☐☐ timer

42. Hvor mange barn passes sammen med barnet i denne barnepass-ordningen?

☐☐ barn

43. Bor du og barnet sammen med barnets far?

- ☐ Ja
- ☐ Nei

44. Hvis barnet ikke bor sammen med sin far, hvor mye er barnet sammen med ham?

- ☐ Minst halvparten av tiden
- ☐ Minst en gang i uken
- ☐ Minst en gang i måneden
- ☐ Sjeldnere enn en gang i måneden
- ☐ Aldri

45. Hvor mange ganger har dere flyttet siden barnet ble født?

☐☐ ganger

46. Hvor stort boareal har den nåværende bolig?

☐☐☐ m^2

47. Har det vært fuktskader, synlig sopp/muggvekst eller mugg-lukt i boligen i løpet av siste år? *(Du kan sette flere kryss.)*

- ☐ Nei
- ☐ Ja, fuktskader
- ☐ Ja, synlig sopp-/muggvekst
- ☐ Ja, mugglukt

48. Hva slags drikkevann er det der dere bor?

- ☐ Vann fra offentlig eller privat vannverk
- ☐ Vann fra egen vannforsyning (f.eks egen brønn)
- ☐ Vet ikke

49. Bor dere i nærheten av en høyspentledning?

- ☐ Nei
- ☐ Ja, nærmere enn 50 meter
- ☐ Ja. 50–100 meter unna
- ☐ Ja, men mer enn 100 meter fra

50. Er det dyr i barnets hjem?

- ☐ Nei
- ☐ Ja

51. Hvis ja, hva slags dyr? *(Sett eventuelt flere kryss.)*

- ☐ Hund
- ☐ Katt
- ☐ Marsvin, kanin, mus, rotte eller lignende
- ☐ Undulat, annen fugl
- ☐ Annet dyr:____________________________________

52. Oppholder barnet seg i rom hvor noen røyker?

- ☐ Ja, daglig. Antall timer per dag ☐☐
- ☐ Ja, flere ganger i uken
- ☐ Ja, av og til
- ☐ Usikker
- ☐ Nei

53. Hvor ofte børstes barnets tenner?

- ☐ 2 ganger pr. dag eller oftere
- ☐ En gang pr. dag
- ☐ Av og til
- ☐ Aldri

54. Brukes fluortannkrem på børsten?

- ☐ Nei
- ☐ Av og til
- ☐ Ja, vanligvis

55. Hvor mye er barnet utendørs?

- ☐ Sjelden
- ☐ Ofte, men gjennomsnittlig mindre enn en time daglig
- ☐ Gjennomsnittlig mer enn en time daglig

56. Hvor mye sitter barnet gjennomsnittlig foran TV/video?

- ☐ 4 timer eller mer
- ☐ 3 timer
- ☐ 1–2 timer
- ☐ Mindre enn 1 time
- ☐ Aldri

57. Deltar eller har barnet deltatt i babysvømming?

- ☐ Nei
- ☐ Ja.

Hvis ja, Hvor lenge: ☐☐ måneder?

58. Bruker barnet narresmokk nå ved 18 måneders alder?

- ☐ Sjelden eller aldri
- ☐ Bare når han/hun skal sove
- ☐ Ganske ofte
- ☐ Mesteparten av tiden

KOMMENTARER:

Er det noe du gjerne vil fortelle oss om barnet som ikke er omhandlet i dette spørreskjemaet, setter vi stor pris på om du noterer det nedenfor.

OM DEG SELV

Helse, sykdom og medisinbruk

59. Hvilken sivilstand har du nå?

☐ Gift
☐ Samboer
☐ Enslig
☐ Separert/skilt
☐ Enke
☐ Annet

60. Er du gravid nå?

☐ Nei
☐ Ja

Hvis ja, hvor mange uker? ☐☐

61. Har du en langvarig sykdom som har oppstått de siste 12 månedene?

☐ Nei
☐ Ja, hvilken? ____

62. Har du selv vært innlagt på sykehus de siste 12 månedene?

☐ Nei
☐ Ja, hvilket sykehus? ______________________________

63. Bruker du nå for tiden tran, vitaminer eller annet kosttilskudd?

☐ Nei
☐ Ja, hvilket?

1.____

2. ____

3. ____

4. ____

64. Har du de siste 12 månedene hatt smerter noen av følgende steder? *(Kryss av for hver linje.)*

	Sjelden/aldri	Litt plaget	En del plaget	Sterkt plaget
1. Magen	☐	☐	☐	☐
2. Armer/bein	☐	☐	☐	☐
3. Nakke/skuldre	☐	☐	☐	☐
4. Hodet	☐	☐	☐	☐
5. Ryggen	☐	☐	☐	☐
6. Bekkenet (bekkenløsning)	☐	☐	☐	☐

65. Hvis du har hatt smerter i rygg eller bekken de siste 12 måneder, kryss av for hvor mye du var plaget på ulike steder:

	En del plaget	Sterkt plaget
1. I korsryggen	☐	☐
2. Over det ene bekkenleddet bak	☐	☐
3. Over begge bekkenleddene bak	☐	☐
4. Over halebeinet	☐	☐
5. I seteballene	☐	☐
6. Foran over kjønnsbenet	☐	☐
7. I lysken	☐	☐
8. Andre ryggsmerter	☐	☐
9. Andre smerter	☐	☐

66. Våkner du om natten pga. bekkensmerter nå for tiden?

☐ Nei, aldri
☐ Ja, en sjelden gang
☐ Ja, ofte

67. Har du nå for tiden så store vansker med å gå pga. bekkensmerter at du må bruke stokk eller krykker?

☐ Nei, aldri
☐ Ja, men ikke hver dag - smerter varierer fra dag til dag
☐ Ja, må bruke stokk eller krykker hver dag

68. Har du fått behandling for bekkensmerter etter siste fødsel?

☐ Nei
☐ Ja

69. Hvis ja, hvilken type behandling har du fått?
(Sett eventuelt flere kryss.)

☐ Fysioterapi
☐ Kiropraktikk
☐ Medikamenter
☐ Annet:____

70. Har du nå for tiden noen av følgende plager? *(Kryss av for hver linje.)*

Plager:	Hvor ofte har du plagene? Aldri	1–4 ganger pr. mnd.	1–6 ganger pr. uke	1 gang pr. dag	Mer enn 1 gang pr. dag	Hvor mye om gangen? Dråper	Større mengder
1. Urinlekkasje ved hosting, nysing eller latter .	☐	☐	☐	☐	☐	☐	☐
2. Urinlekkasje ved fysisk aktivitet (løp/hopp) . .	☐	☐	☐	☐	☐	☐	☐
3. Urinlekkasje ved sterk trang til vannlating . . .	☐	☐	☐	☐	☐	☐	☐
4. Problemer med å holde på avføring	☐	☐	☐	☐	☐		
5. Problemer med å holde på luft	☐	☐	☐	☐	☐		

71. Bruker du medisiner jevnlig?

☐ Nei
☐ Ja

72. Hvis ja, oppgi navn på medisinene og hvor ofte du bruker dem. *(Ta med alle typer medisiner, også naturmedisiner.)*

Navn på medisinen (*f.eks. Apocillin, Paracet*)	Hvor ofte bruker du dem? Hver dag	Daglig i perioder	Av og til
________________________________	☐	☐	☐
________________________________	☐	☐	☐
________________________________	☐	☐	☐
________________________________	☐	☐	☐

Økonomi – livsstil

73. Hvor lang permisjon tok du/dere etter fødselen? *(Oppgi enten antall måneder eller uker.)*

	Måneder		Uker
Deg selv	☐☐	eller	☐☐
Barnets far	☐☐	eller	☐☐

74. Har du nå inntektsgivende arbeid?

☐ Nei
☐ Ja

75. Hvis ja, hvor mange timer arbeider du pr. uke?

☐☐ timer

76. Hvis du er i lønnet arbeid, har du vært sykemeldt siden du begynte å arbeide igjen? Hvis ja, oppgi hvor mange dager du var sykemeldt.

	Antall dager
☐ Nei	
☐ Ja, på grunn av egen sykdom.	☐☐
☐ Ja, på grunn av sykdom hos barn.	☐☐

77. Er økonomien slik at du/dere vil ha mulighet til å klare en uforutsett regning på kr. 3000 til f. eks. tannlege eller en reparasjon?

☐ Nei
☐ Ja
☐ Vet ikke

78. Har det i løpet av det siste halve året hendt at du/dere har hatt vansker med å klare løpende utgifter til mat, transport, husleie og lignende?

☐ Nei, aldri
☐ Ja, en sjelden gang
☐ Ja, av og til
☐ Ja, ofte

79. Hvor ofte er du så fysisk aktiv (i fritid eller på arbeid) at du blir andpusten eller svett?

	I fritiden	På arbeid
1. Aldri	☐	☐
2. Mindre enn 1 gang pr. uke .	☐	☐
3. 1 gang pr. uke	☐	☐
4. 2 ganger pr. uke	☐	☐
5. 3–4 ganger per uke	☐	☐
6. 5 ganger eller mer pr. uke . .	☐	☐

80. Hvor ofte driver du fysisk aktivitet nå for tiden? *(Kryss av for hver linje.)*

Aktivitet	Aldri	1–3 ganger pr. mnd	1 gang pr. uke	2 ganger pr. uke	3 ganger eller mer pr. uke
1. Rolig gange/spasertur	☐	☐	☐	☐	☐
2. Rask gange/turgang	☐	☐	☐	☐	☐
3. Løping/jogging/orientering	☐	☐	☐	☐	☐
4. Sykling	☐	☐	☐	☐	☐
5. Helsestudio/styrketrening	☐	☐	☐	☐	☐
6. Aerobics/gymnastikk/dans uten løp og hopp	☐	☐	☐	☐	☐
7. Aerobics/gymnastikk/dans med løp og hopp	☐	☐	☐	☐	☐
8. Dansing (f.eks. swing, rock, folkedans)	☐	☐	☐	☐	☐
9. Skigåing	☐	☐	☐	☐	☐
10. Ballspill/nettballspill	☐	☐	☐	☐	☐
11. Svømming	☐	☐	☐	☐	☐
12. Riding	☐	☐	☐	☐	☐
13. Annet	☐	☐	☐	☐	☐

81. Hvordan er røykevanene i hjemmet nå?

	Deg selv	Din samboer/ ektefelle
1. Røyker ikke	☐	☐
2. Røyker av og til	☐	☐
3. Røyker daglig	☐	☐
4. Hvis daglig, antall sigaretter per dag	☐☐	☐☐

82. Hvor ofte drikker du alkohol nå for tiden?

- ☐ Omtrent 6–7 ganger pr. uke
- ☐ Omtrent 4–5 ganger pr.uke
- ☐ Omtrent 2–3 ganger pr.uke
- ☐ Omtrent 1 gang pr.uke
- ☐ Omtrent 1–3 ganger pr. måned
- ☐ Sjeldnere enn 1 gang pr. måned
- ☐ Aldri

83. Hvor mange alkoholenheter drikker du vanligvis når du nyter alkohol? Kryss av for både i helgen og hverdagen. *(Se forklaring nedenfor.)*

	I helgen	Hverdager
10 eller flere	☐	☐
7-9	☐	☐
5-6	☐	☐
3-4	☐	☐
1-2	☐	☐
Færre enn 1	☐	☐

Enheter alkohol
For å sammenligne ulike typer alkohol, spør vi etter det vi kaller alkoholenheter (= 1,5cl ren alkohol). I praksis betyr dette følgende:

1 glass (1/3 liter) øl	= 1 enhet
1 vinglass rød eller hvit vin	= 1 enhet
1 hetvinsglass, sherry eller annen hetvin	= 1 enhet
1 drammeglass brennevin eller likør	= 1 enhet
1 flaske rusbrus/cider	= 1 enhet

Litt mer om deg selv og hvordan du har det nå

84. Hvis du har en samboer/ektefelle, hvor enig er du i disse beskrivelsene? *(Kryss av for hver linje.)*

	Svært enig	Enig	Litt enig	Litt uenig	Uenig	Svært uenig
1. Det er et nært samhold mellom meg og min ektefelle/samboer/partner	☐	☐	☐	☐	☐	☐
2. Min partner og jeg har problemer i parforholdet	☐	☐	☐	☐	☐	☐
3. Jeg er svært lykkelig i mitt parforhold	☐	☐	☐	☐	☐	☐
4. Min partner er generelt forståelsesfull	☐	☐	☐	☐	☐	☐
5. Jeg tenker ofte på å avslutte vårt parforhold	☐	☐	☐	☐	☐	☐
6. Jeg er fornøyd med forholdet til min partner	☐	☐	☐	☐	☐	☐
7. Vi er ofte uenige om viktige avgjørelser	☐	☐	☐	☐	☐	☐
8. Jeg har vært heldig med valg av partner	☐	☐	☐	☐	☐	☐
9. Vi er enige om hvordan barn bør oppdras	☐	☐	☐	☐	☐	☐
10. Jeg tror min partner er fornøyd med forholdet	☐	☐	☐	☐	☐	☐

85. Har du noen utenom din ektefelle/samboer/partner som du kan søke råd hos i en vanskelig situasjon?

☐ Nei
☐ Ja, 1-2 personer
☐ Ja, flere enn 2 personer

86. Hvor ofte treffer du eller snakker i telefonen med familie (utenom husholdningen) eller nære venner?

☐ 1 gang i måneden eller sjeldnere
☐ 2-8 ganger i måneden
☐ Mer enn 2 ganger i uken

87. Føler du deg ofte ensom?

☐ Nesten aldri
☐ Sjelden
☐ Av og til
☐ Som regel
☐ Nesten alltid

88. Hvor riktig er disse utsagnene for deg? *(Kryss av for hver linje.)*

	Ikke riktig	Litt riktig	Nesten riktig	Helt riktig
1. Jeg klarer alltid å løse vanskelige problemer hvis jeg prøver hardt nok	☐	☐	☐	☐
2. Hvis noen motarbeider meg, finner jeg en måte å oppnå det jeg vil på	☐	☐	☐	☐
3. Jeg er sikker på at jeg kan mestre uventede hendelser	☐	☐	☐	☐
4. Jeg er rolig når jeg møter vanskeligheter fordi jeg stoler på min evne til å klare meg	☐	☐	☐	☐
5. Dersom jeg er i en knipe, finner jeg vanligvis en løsning	☐	☐	☐	☐

89. Hvor ofte opplever du følgende i ditt daglige liv? *(Kryss av for hver linje.)*

	Sjelden/ aldri	Nokså sjelden	Noen ganger	Ofte	Veldig ofte
1. Føler deg glad for noe	☐	☐	☐	☐	☐
2. Føler deg lykkelig	☐	☐	☐	☐	☐
3. Føler deg oppstemt, som om alt legger seg til rette for deg	☐	☐	☐	☐	☐
4. Føler at du vil skrike til noen eller slå løs på ting	☐	☐	☐	☐	☐
5. Føler deg sint, irritert eller ergerlig	☐	☐	☐	☐	☐
6. Føler deg rasende på noen	☐	☐	☐	☐	☐

90. Hva slags oppfatning har du av deg selv? *(Kryss av for hver linje.)*

	Svært enig	Enig	Uenig	Svært uenig
1. Jeg har en positiv holdning til meg selv	☐	☐	☐	☐
2. Jeg føler meg virkelig ubrukelig til tider	☐	☐	☐	☐
3. Jeg føler at jeg ikke har mye å være stolt av	☐	☐	☐	☐
4. Jeg føler at jeg er en verdifull person, iallfall på lik linje med andre	☐	☐	☐	☐

91. Har du i løpet av de 2 siste ukene vært plaget med noe av det følgende? *(Kryss av for hver linje.)*

	Ikke plaget	Litt plaget	Ganske mye plaget	Veldig mye plaget
1. Stadig redd eller engstelig	☐	☐	☐	☐
2. Nervøsitet, indre uro	☐	☐	☐	☐
3. Følelse av håpløshet med hensyn til fremtiden	☐	☐	☐	☐
4. Nedtrykt, tungsindig	☐	☐	☐	☐
5. Mye bekymret eller urolig	☐	☐	☐	☐
6. Følelse av at alt er et slit	☐	☐	☐	☐
7. Føler deg anspent eller oppjaget	☐	☐	☐	☐
8. Plutselig frykt uten grunn	☐	☐	☐	☐

92. Har du opplevd noe av det følgende det siste året (siden forrige spørreskjema)? Hvis ja, hvor vondt eller vanskelig var dette for deg? *(Kryss av for hver linje.)*

	Nei	Ja	Hvis ja: Ikke så ille	Vondt/ vanskelig	Veldig vondt/ vanskelig
1. Problemer på arbeidsplassen eller der du utdanner deg	☐	☐	☐	☐	☐
2. Økonomiske problemer	☐	☐	☐	☐	☐
3. Ble skilt, separert eller avbrøt samlivet	☐	☐	☐	☐	☐
4. Problemer eller konflikter med familie, venner eller naboer	☐	☐	☐	☐	☐
5. Alvorlig bekymring for om det er noe galt med barnet	☐	☐	☐	☐	☐
6. Alvorlig sykdom eller skade (deg selv)	☐	☐	☐	☐	☐
7. En av de nærmeste har vært alvorlig syk eller skadet	☐	☐	☐	☐	☐
8. Alvorlig trafikkulykke, boligbrann eller grovt tyveri	☐	☐	☐	☐	☐
9. Mistet en som sto deg nær	☐	☐	☐	☐	☐
10. Blitt presset til seksuelle handlinger	☐	☐	☐	☐	☐
11. Annet	☐	☐	☐	☐	☐

93. Hvordan vil du vurdere kvaliteten på livet ditt?

- ☐ Svært dårlig
- ☐ Dårlig
- ☐ Verken god eller dårlig
- ☐ God
- ☐ Svært god

94. Hvor tilfreds er du med helsen din?

- ☐ Svært utilfreds
- ☐ Utilfreds
- ☐ Verken tilfreds eller utilfreds
- ☐ Tilfreds
- ☐ Svært tilfreds

95. De følgende spørsmålene spør etter hvor mye du har opplevd av bestemte ting i de siste to ukene. *(Kryss av for hver linje.)*

	Ikke i det hele tatt	Litt	Til en viss grad	I høy grad	I svært høy grad
1. I hvilken grad føler du at smerte hindrer deg i å gjøre det du må?	☐	☐	☐	☐	☐
2. I hvilken grad trenger du medisinsk behandling for å kunne fungere til daglig?	☐	☐	☐	☐	☐
3. Hvor mye gleder du deg over livet?	☐	☐	☐	☐	☐
4. I hvilken grad føler du at livet ditt er meningsfullt?	☐	☐	☐	☐	☐
5. Hvor godt kan du konsentrere deg?	☐	☐	☐	☐	☐
6. Hvor trygg føler du deg til daglig?	☐	☐	☐	☐	☐
7. Hvor sunne er dine fysiske omgivelser?	☐	☐	☐	☐	☐

96. De følgende spørsmålene spør etter hvor fullstendig du opplevde eller kunne utføre bestemte ting i løpet av de siste to ukene. *(Kryss av for hver linje.)*

	Ikke i det hele tatt	Litt	Til en viss grad	I høy grad	Full-stendig
1. Har du nok energi til dine daglige gjøremål?	☐	☐	☐	☐	☐
2. Kan du akseptere utseende ditt?	☐	☐	☐	☐	☐
3. Har du nok penger til å dekke dine behov?	☐	☐	☐	☐	☐
4. Hvor tilgjengelig er den infomasjonen som du trenger i dagliglivet?	☐	☐	☐	☐	☐
5. I hvilken grad har du mulighet for å delta i fritidsaktiviteter?	☐	☐	☐	☐	☐

97. Hvor godt er du i stand til å komme deg dit du vil?

- ☐ Svært dårlig
- ☐ Dårlig
- ☐ Verken godt eller dårlig
- ☐ Godt
- ☐ Svært godt

98. De følgende spørsmålene spør etter hvor glad eller tilfreds du har følt deg over bestemte sider ved livet i de siste to ukene.
(Kryss av for hver linje.)

	Svært utilfreds	Util- freds	Verken tilfreds eller utilfreds	Tilfreds	Svært tilfreds
1. Hvor tilfreds er du med hvordan du sover?	☐	☐	☐	☐	☐
2. Hvor tilfreds er du med din evne til å utføre dine daglige gjøremål?	☐	☐	☐	☐	☐
3. Hvor tilfreds er du med din arbeidskapasitet?	☐	☐	☐	☐	☐
4. Hvor tilfreds er du med deg selv?	☐	☐	☐	☐	☐
5. Hvor tilfreds er du med ditt forhold til andre mennesker?	☐	☐	☐	☐	☐
6. Hvor tilfreds er du med ditt seksualliv?	☐	☐	☐	☐	☐
7. Hvor tilfreds er du med den støtten du får fra dine venner?	☐	☐	☐	☐	☐
8. Hvor tilfreds er du med forholdene der du bor?	☐	☐	☐	☐	☐
9. Hvor tilfreds er du med din tilgang til helsetjenester?	☐	☐	☐	☐	☐
10. Hvor tilfreds er du med transportmulighetene dine?	☐	☐	☐	☐	☐

99. Det følgende spørsmålet refererer seg til hvor ofte du har opplevd eller følt negative følelser i løpet av de siste to ukene?

	Aldri	Sjelden	Ofte	Svært ofte	Alltid
Hvor ofte har du opplevd negative følelser, som f.eks. at du er trist, fortvilet, engstelig eller deprimert?	☐	☐	☐	☐	☐

KOMMENTARER:

Har du husket å fylle ut dato for utfylling av skjema på side 1?

Tusen takk for innsatsen!

Legg det utfylte skjemaet i den frankerte returkonvolutten og send det til:

Den norske Mor og Barn undersøkelsen
Medisinsk fødselsregister
Armauer Hansens Hus
5839 Bergen
